# Supplementary material for: The chemotherapeutic CX-5461 primarily targets TOP2B and exhibits selective activity in high-risk neuroblastoma
Source: Nat Commun. 2021 Nov 9;12:6468. doi: 10.1038/s41467-021-26640-x (PMC8578635; doi:10.1038/s41467-021-26640-x)
Supplement: Supplementary file 1 — Supplementary Information [file 41467_2021_26640_MOESM1_ESM.pdf]

SUPPLEMENTARY FIGURES

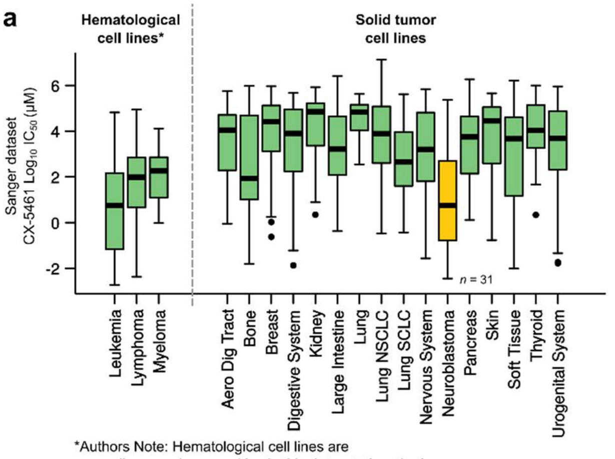

*Authors Note: Hematological cell lines are generally more drug sensitive in this dataset, thus the low IC_{50} values in hematological cell lines do not imply selective activity.

b

Cell line	IC50 (μM) CX-5461	P53	MYCN Copy Number
IMR-5	0.018	WT	14
CHP-134	0.041	WT	12
KELLY	0.589	Mut	14
BE(2)-M17	5.491	Mut	14
SK-N-SH	0.220	Mut	3
SK-N-FI	27.267	Mut	2
RS4-11	0.0684	Sensitive cell control	
H2452	31.5601	Resistant cell control	

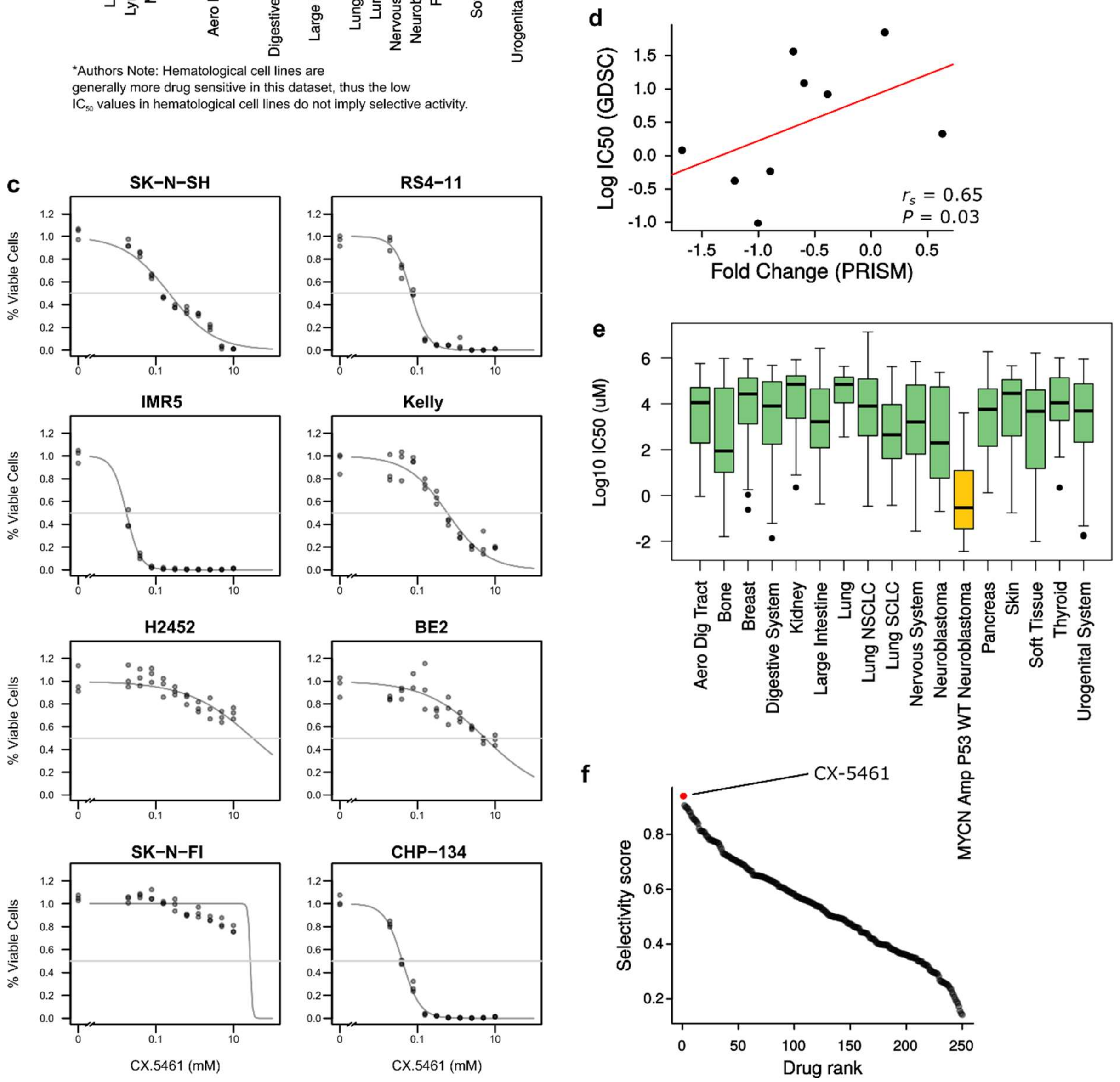

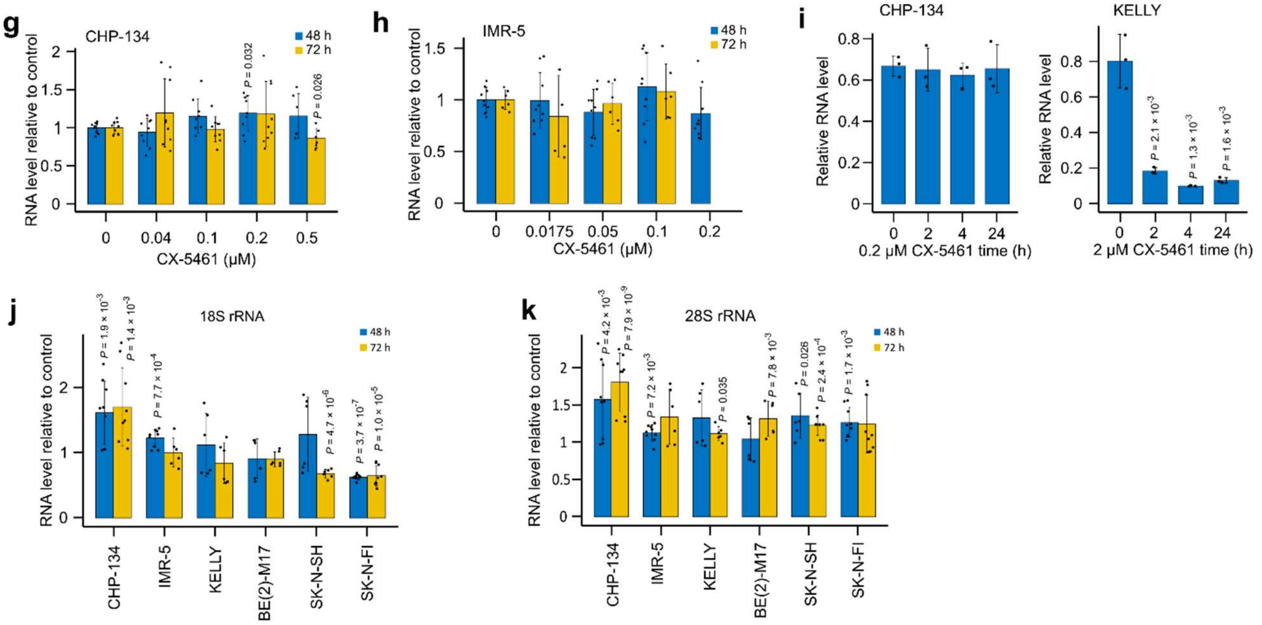

Figure S1

a. Boxplot of all IC_{50} values for CX-5461 from the 2016 release of the GDSC dataset with hematological cancer cell lines (Iorio et al, Cell 2016). Note that hematological cell lines tend to be generally more drug sensitive in these screens and a low IC_{50} compared to other cell lines does not imply selective killing.
b. Table showing IC_{50} values obtained and other relevant details from our re-screen of a subset of 8 GDSC cell lines with CX-5461.
c. Cell viability data for the 8 cell lines, showing the percentage of viable cells (y-axis) against the concentration of CX-5461 (x-axis). Dose-response curves were estimated using a generalized logistic regression model fit to the data using the R package “drc”.
d. Scatterplot of log IC_{50} values from GDSC (y-axis) plotted against fold change values in PRISM (x-axis) for CX-5461 for the 9 neuroblastoma cell lines that were screened in both datasets; the *P*-value was estimated using a one-sided Spearman's Rank Correlation test.
e. Boxplot of $\log_{10}(IC_{50})$ values for CX-5461 from all solid tumor cell lines in GDSC. Hematological cancers are not included. N = 17 biologically independent *MYCN* amplified *TP53* WT neuroblastoma cell lines have been separated from other neuroblastoma cell lines.
f. Waterfall plot showing Selectivity Scores (y-axis) for *MYCN* amplified *TP53* WT neuroblastoma cell lines for 265 compounds screened by GDSC.

g-h. 45S pre-rRNA expression levels (y-axis) in CX-5461 treated CHP-134 and IMR-5 cells were measured by qPCR. Data represent mean ± SD of n = 9 biological replicates.

i. CHP-134 and KELLY cells were treated with 0.2 μM and 2 μM CX-5461, respectively, for the time indicated. 45S pre-rRNA expression levels were measured by qPCR. Data represent mean ± SD of n = 3 biological replicates. j-k. 18S and 28S rRNA expression (y-axis) in CX-5461 treated neuroblastoma cell lines. Data represent mean ± SD of n = 9 biological replicates. CX-5461 concentration: CHP-134, 0.2 μM; IMR-5, 0.05 μM; KELLY, 2 μM; BE(2)-M17, 10 μM; SK-NSH, 2 μM; SK-N-FI, 20 μM.

In all boxplots, the center line represents the median, bound of box is upper and lower quartiles and whiskers are 1.5× the interquartile range. Two-tailed student's t-test was used for Fig. S1g-k. Source data for Fig. S1c, g-k are included in Source Data file.

a

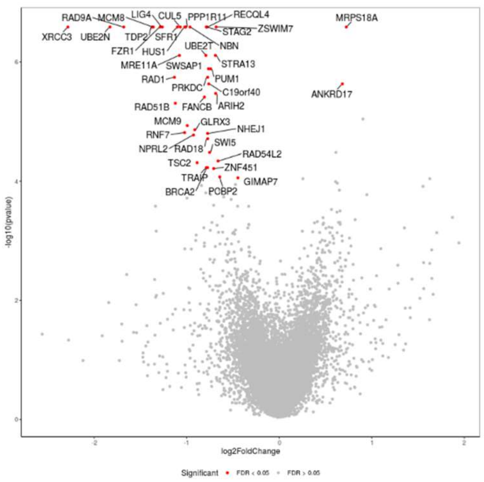

b

Index	Name	P-value	Adjusted p-value	Odds Ratio	Combined score
1	recombinational repair (GO:0000725)	1.067e-18	2.723e-15	77.46	3205.61
2	double-strand break repair via homologous recombination (GO:0000724)	2.371e-18	4.033e-15	72.37	2936.94
3	DNA recombinase assembly (GO:0000730)	4.121e-7	0.0002337	187.50	2756.62
4	DNA repair complex assembly (GO:0090735)	4.121e-7	0.0002103	187.50	2756.62
5	double-strand break repair via synthesis-dependent strand annealing (GO:0045003)	4.121e-7	0.0001912	187.50	2756.62
6	double-strand break repair (GO:0006302)	8.503e-23	4.339e-19	53.19	2703.14
7	strand invasion (GO:0042148)	0.00008138	0.01978	142.86	1345.19
8	response to ionizing radiation (GO:0010212)	1.597e-10	1.164e-7	45.45	1025.36
9	telomeric loop disassembly (GO:0090657)	0.0001737	0.03410	100.00	865.80
10	DNA recombination (GO:0006310)	7.983e-8	0.00005092	45.45	742.88

c

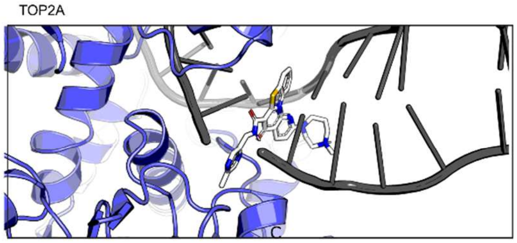

d

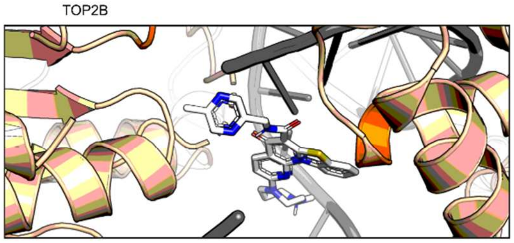

e

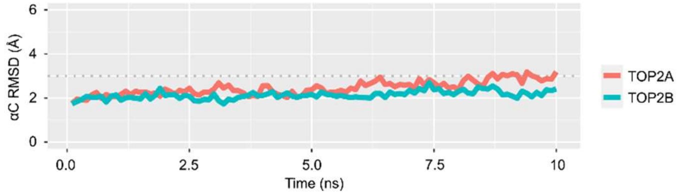

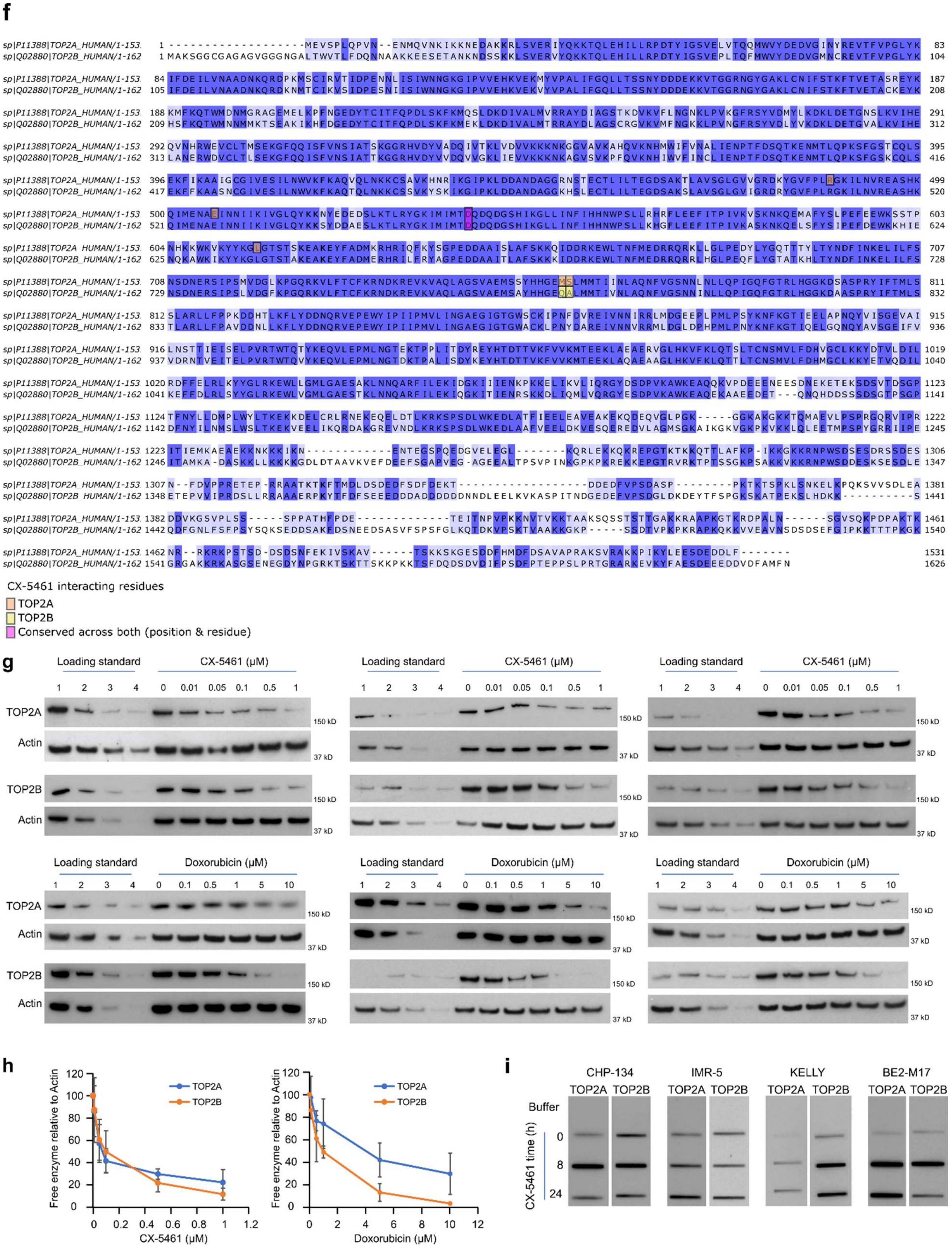

f
sp|P11388|TOP2A_HUMAN/1-153
sp|Q02880|TOP2B_HUMAN/1-162
CX-5461 interacting residues
TOP2A
TOP2B
Conserved across both (position & residue)
g
Loading standard
CX-5461 (µM)
1 2 3 4 0 0.01 0.05 0.1 0.5 1
TOP2A
Actin
TOP2B
150 kD
37 kD
Doxorubicin (µM)
1 2 3 4 0 0.1 0.5 1 5 10
h
Free enzyme relative to Actin
TOP2A
TOP2B
CX-5461 (µM)
Doxorubicin (µM)
i
CHP-134
IMR-5
KELLY
BE2-M17
Buffer
CX-5461 time (h)
0
8
24

Figure S2.

a. Volcano plot showing the results of our genome wide CRISPR knockout screen in CHP-134 cells. The x-axis shows the mean fold change for the normalized abundance of the guide RNAs targeting each gene in CX-5461 compared to DMSO treated control cells. P-values are shown on the y-axis and significant knockouts, at a false discovery rate of <5%, are highlighted in red.
b. Table of the top pathways from our genome wide CRISPR knockout screen in CHP-134 cells as reported by the Enrichr software for genes at an FDR <5%.

c-d. Closeup views of CX-5461 binding to TOP2A (c) or TOP2B (d) resulting from molecular dynamics simulations.

e. The root-mean square deviation (RMSD) from the simulations overlaid for both systems. Calculations are in reference to the alpha-Carbon atoms.
f. Protein sequence alignment of Human TOP2A and TOP2B, colored by conserved (dark blue) or non-conserved (light blue) resides. CX-5461-interacting residues based on molecular dynamics simulations are highlighted in orange (found in TOP2A), yellow (found in TOP2B), or pink (conserved position and residue in both).
g. Band-depletion assay for CX-5461 (top) and doxorubicin (bottom) treated CHP-134 cells. Cells were exposed to agents as indicated for 4 h. Cell lysates were collected for SDS-PAGE. Cell lysates from untreated cells were used as loading standard. For lane 1 - 4, protein equal to 33, 16.5, 8.3, and 4.1 thousand cells were loaded respectively. Protein equal to 33 thousand cells were loaded to each of the rest of the lanes. 3 replicates are shown for each drug. Replicate 1 is also shown in Fig. 2k-l.
h. Quantification of the images in Fig. S2g. Free enzyme relative to Actin was calculated by normalizing the signal to DMSO (0 μM) treated samples. Data represent mean ± SD of n = 3 biological replicates. The source data are included in Source Data file.
i. RADAR assay in neuroblastoma cells. Cells were treated with CX-5461 or DMSO (0 h) for the time indicated. DNA – topoisomerase covalent complexes were extracted and visualized using TOP2A and TOP2B antibodies. Representative results of 3 independent replicates are shown. CX-5461 concentration used for each cell line: CHP-134, 0.2 μM; IMR-5, 0.05 μM; KELLY, 2 μM; BE(2)-M17, 10 μM.

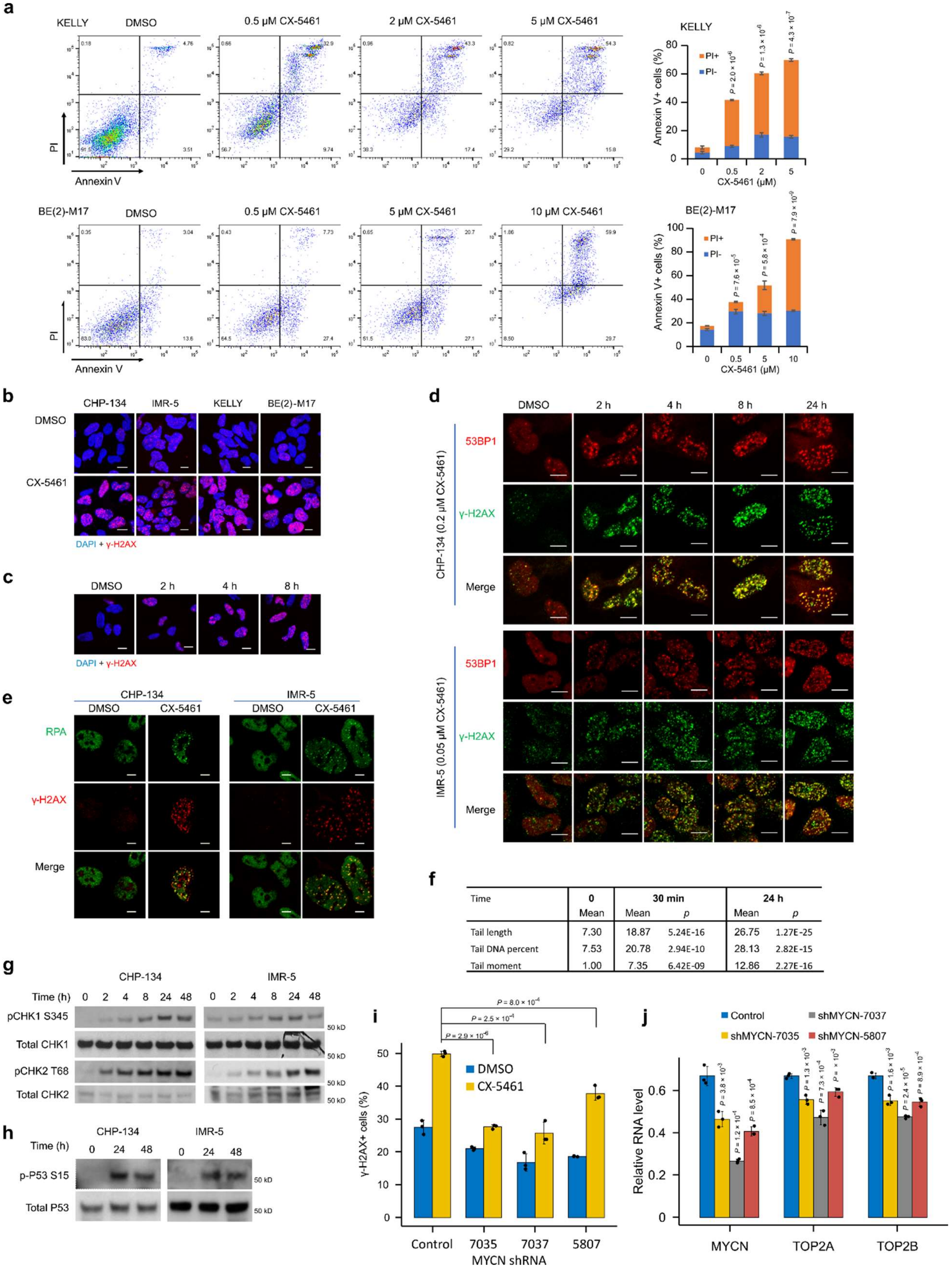

Time	0	30 min		24 h	
	Mean	Mean	p	Mean	p
Tail length	7.30	18.87	5.24E-16	26.75	1.27E-25
Tail DNA percent	7.53	20.78	2.94E-10	28.13	2.82E-15
Tail moment	1.00	7.35	6.42E-09	12.86	2.27E-16

Figure S3.

a. Flow cytometry analysis for apoptosis in KELLY and BE(2)-M17 cells. Cells were treated with CX-5461 for 48 h with indicated concentrations. Induction of apoptosis was measured by annexin V (x-axis) and cell death by propidium iodide (PI) staining (y-axis). Representative cell distribution (left) and quantification of 3 replicates (right, data represent mean ± SD of n = 3 biological replicates) are shown.
b. CX-5461 induced γ-H2AX foci in neuroblastoma cell lines. Cells were treated with DMSO or CX-5461 for 24 h. Nuclei were stained with DAPI (blue) and phosphorylated histone H2AX was stained with γ-H2AX antibody (red). Representative results of 3 independent replicates are shown. CX-5461 concentration: CHP-134, 0.2 µM; IMR-5, 0.05 µM; KELLY, 2 µM; BE(2)-M17, 5 µM. Scale bar = 10 µm.
c. CX-5461 induced γ-H2AX foci in CHP-134 cells. Cells were treated with DMSO or 0.2 µM CX-5461 for the time indicated. Nuclei were stained with DAPI (blue) and phosphorylated histone H2AX was stained with γ-H2AX antibody (red). Representative results of 3 independent replicates are shown. Scale bar = 10 µm.
d. Co-localization of 53BP1 and γ-H2AX in CHP-134 (top) and IMR-5 (bottom) cells. Cells were treated with DMSO or CX-5461 (0.2 µM for CHP-134 and 0.05 µM for IMR-5) for the time indicated, then stained with 53BP1 (red) and γ-H2AX (green) antibodies for immunofluorescence. Representative results of 3 independent replicates are shown. Images at 0 h (DMSO) and 24 h are also shown in Figure 3e. Scale bar = 10 µm.
e. CX-5461 induced RPA and γ-H2AX foci. After treated with DMSO or CX-5461 (0.2 µM for CHP-134 and 0.05 µM for IMR-5) for 24 h, cells were stained with RPA (green) and γ-H2AX (red) antibodies for immunofluorescence. Representative results of 3 independent replicates are shown. Scale bar = 5 µm.
f. Quantification of comet assay in Figure 3f. Tail length, tail DNA percent, and tail moment were calculated with OpenComet image analysis tool. *P*-values are from 2-tailed t-test.
g. Western blots for CHK1, CHK2 and phosphorylated CHK1 and CHK2. Cells were treated with CX-5461 (CHP-134, 0.2 µM; IMR-5, 0.05 µM) for the time indicated. β-Actin was used as a loading control. Representative results of 2 independent replicates are shown.
h. Western blots for p53 and phosphorylated p53. Cells were treated with CX-5461 (CHP-134, 0.2 µM; IMR-5, 0.05 µM) for the time indicated. β-Actin was used as a loading control. Representative results of 2 independent replicates are shown.
i. Quantification of flow cytometry data showing γ-H2AX levels in MYCN knockdown cells. CHP-134 cells were transduced with doxycycline inducible MYCN shRNA. The cells were cultured in medium with 0.5 ug/ml puromycin and 2 ug/ml doxycycline for 5 days, then in medium with 2 ug/ml doxycycline and 0.2 uM CX-5461 (or DMSO) for 24h. Cells were stained with γ-H2AX for flow cytometry. Data represent mean ± SD of n = 3 biological replicates.
j. qPCR for TOP2A and TOP2B expression in MYCN knockdown cells. CHP-134 cells were transduced with doxycycline inducible MYCN shRNA. The cells were cultured in medium with 0.5 ug/ml puromycin and 2 ug/ml doxycycline for 6 days, then RNA was extracted for qPCR. Data represent mean ± SD of n = 3 biological replicates.

Two-tailed student's t-test was used for Fig. S3a, i, j. Source data for these panels are included in Source Data file.

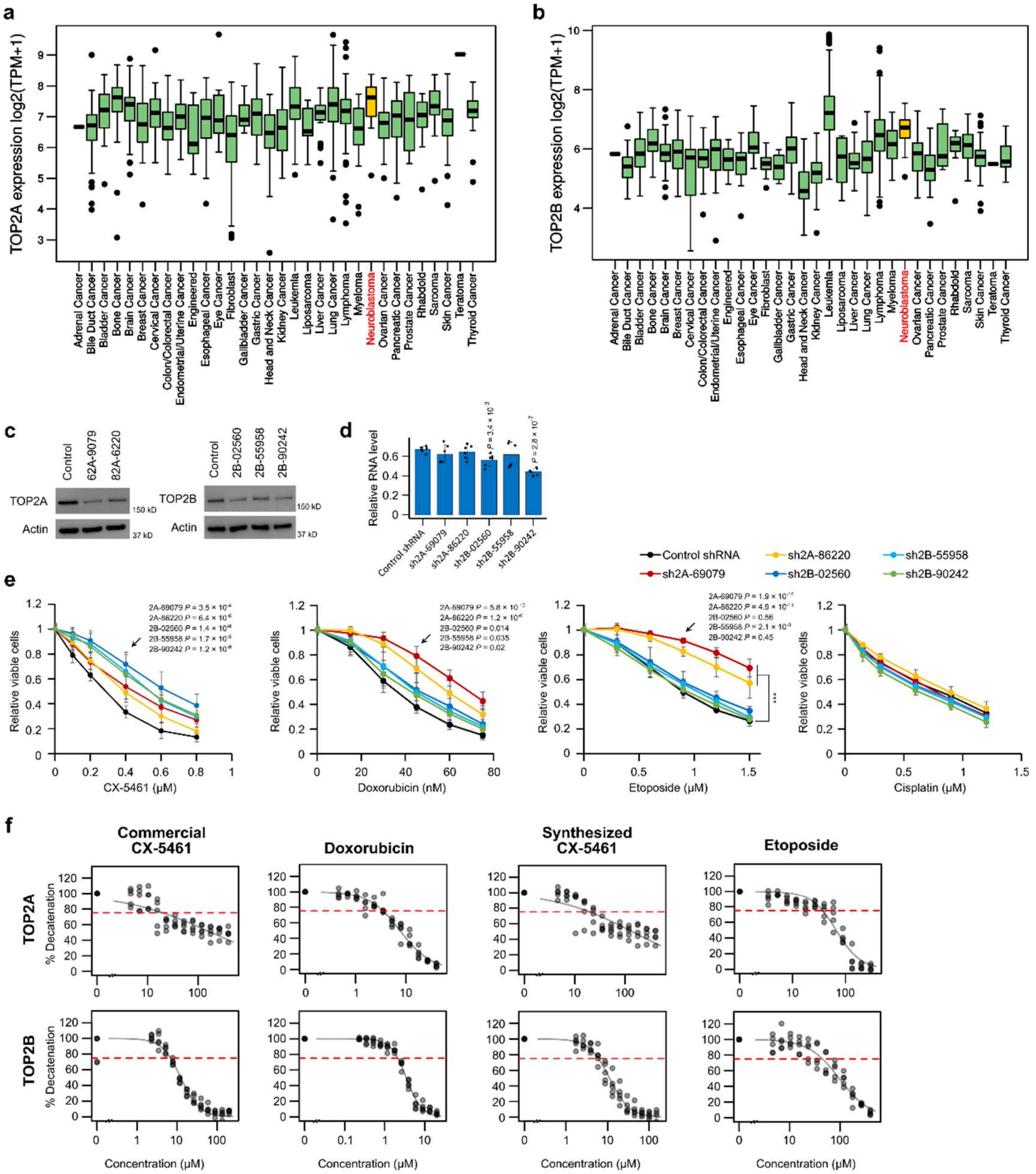
a
TOP2A expression log2(TPM+1)
b
TOP2B expression log2(TPM+1)
Adrenal Cancer
Bile Duct Cancer
Bladder Cancer
Bone Cancer
Brain Cancer
Breast Cancer
Cervical Cancer
Colon/Colorectal Cancer
Endometrial/Uterine Cancer
Engineered
Esophageal Cancer
Eye Cancer
Fibroblast
Gallbladder Cancer
Gastric Cancer
Head and Neck Cancer
Kidney Cancer
Leukemia
Liposarcoma
Liver Cancer
Lung Cancer
Lymphoma
Myeloma
Neuroblastoma
Ovarian Cancer
Pancreatic Cancer
Prostate Cancer
Rhabdoid
Sarcoma
Skin Cancer
Teratoma
Thyroid Cancer
c
Control
TOP2A
TOP2B
Actin
150 kD
37 kD
2B-02560
2B-55958
2B-90242
d
Relative RNA level
Control shRNA
sh2A-69079
sh2A-86220
sh2B-02560
sh2B-55958
sh2B-90242
e
Control shRNA
sh2A-69079
sh2A-86220
sh2B-02560
sh2B-55958
sh2B-90242
Relative viable cells
CX-5461 (μM)
Doxorubicin (nM)
Etoposide (μM)
Cisplatin (μM)
f
Commercial CX-5461
Doxorubicin
Synthesized CX-5461
Etoposide
TOP2A
TOP2B
% Decatenation
Concentration (μM)

g

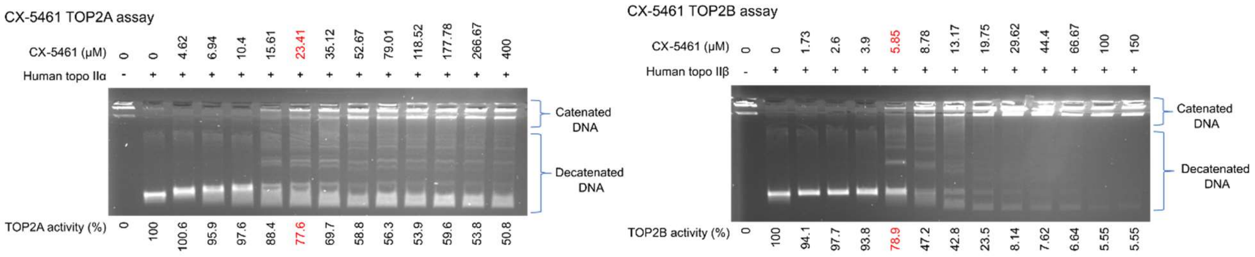
CX-5461 TOP2A assay
CX-5461 (µM) 0 0 4.62 6.94 10.4 15.61 23.41 35.12 52.67 79.01 118.52 177.78 266.67 400
Human topo IIα - + + + + + + + + + + + + +
Catenated DNA
Decatenated DNA
TOP2A activity (%) 0 100 100.6 95.9 97.6 88.4 77.6 69.7 58.8 56.3 53.9 59.6 53.8 50.8
CX-5461 TOP2B assay
CX-5461 (µM) 0 0 1.73 2.6 3.9 5.85 8.78 13.17 19.75 29.62 44.4 66.67 100 150
Human topo IIβ - + + + + + + + + + + + + +
Catenated DNA
Decatenated DNA
TOP2B activity (%) 0 100 94.1 97.7 93.8 78.9 47.2 42.8 23.5 8.14 7.62 6.64 5.55 5.55

h

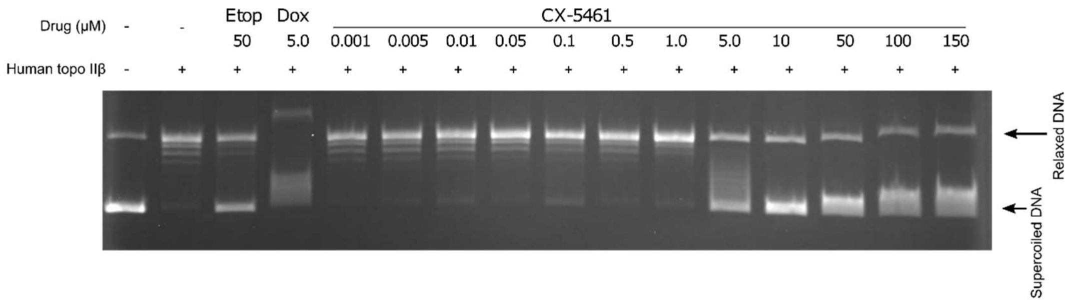
Drug (µM) - - Etop 50 Dox 5.0
CX-5461
0.001 0.005 0.01 0.05 0.1 0.5 1.0 5.0 10 50 100 150
Human topo IIβ - + + + + + + + + + + + + + + +
Relaxed DNA
Supercoiled DNA

i

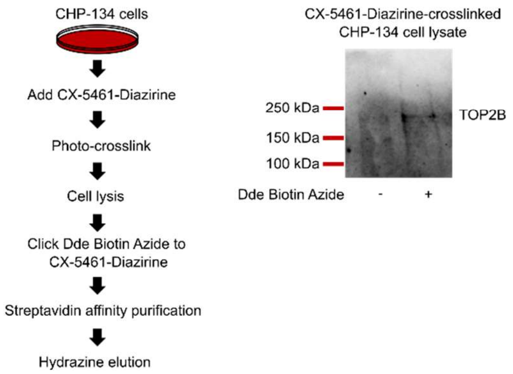
CHP-134 cells
Add CX-5461-Diazirine
Photo-crosslink
Cell lysis
Click Dde Biotin Azide to CX-5461-Diazirine
Streptavidin affinity purification
Hydrazine elution
CX-5461-Diazirine-crosslinked CHP-134 cell lysate
250 kDa
150 kDa
100 kDa
TOP2B
Dde Biotin Azide - +

j

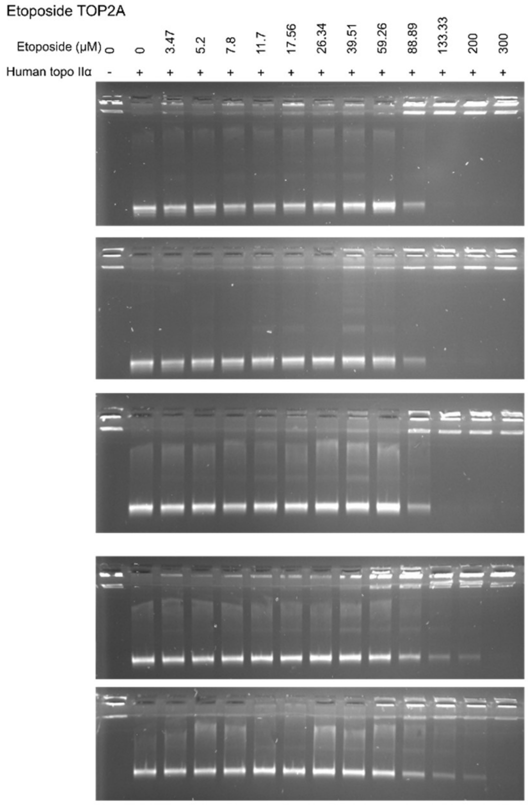
Etoposide TOP2A
Etoposide (μM) 0 0 3.47 5.2 7.8 11.7 17.56 26.34 39.51 59.26 88.89 133.33 200 300
Human topo IIα - + + + + + + + + + + + + +

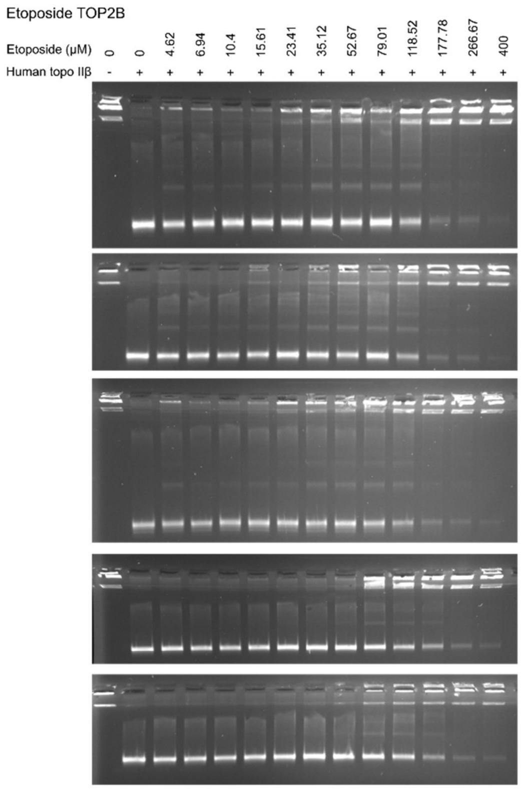
Etoposide TOP2B
Etoposide (μM) 0 0 4.62 6.94 10.4 15.61 23.41 35.12 52.67 79.01 118.52 177.78 266.67 400
Human topo IIβ - + + + + + + + + + + + + +

k

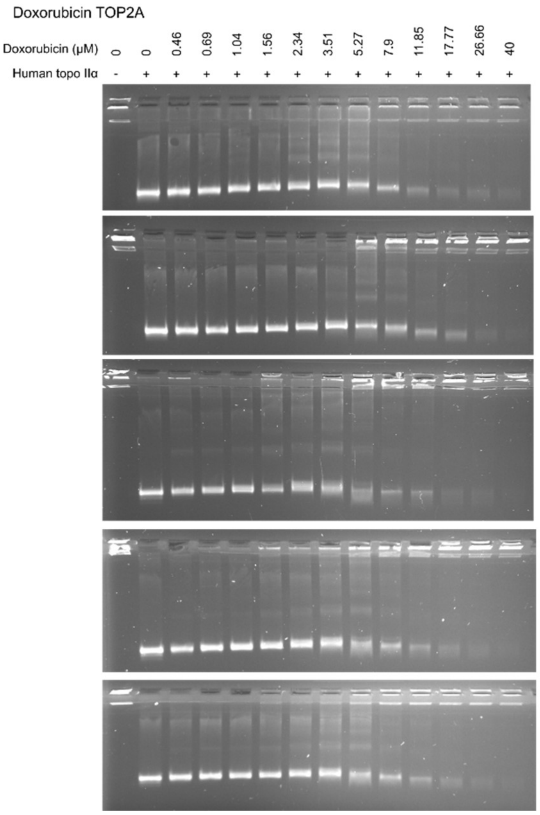
Doxorubicin TOP2A
Doxorubicin (μM) 0 0 0.46 0.69 1.04 1.56 2.34 3.51 5.27 7.9 11.85 17.77 26.66 40
Human topo IIα - + + + + + + + + + + + + +

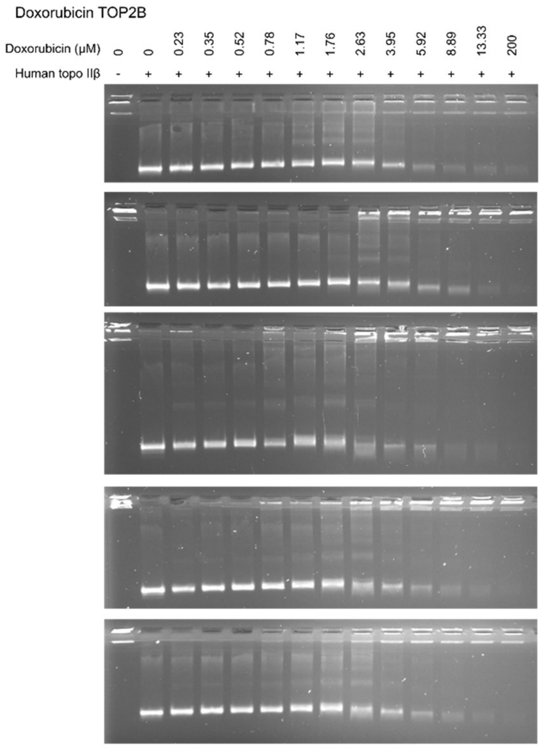
Doxorubicin TOP2B
Doxorubicin (μM) 0 0 0.23 0.35 0.52 0.78 1.17 1.76 2.63 3.95 5.92 8.89 13.33 200
Human topo IIβ - + + + + + + + + + + + + +

l CX-5461 TOP2A

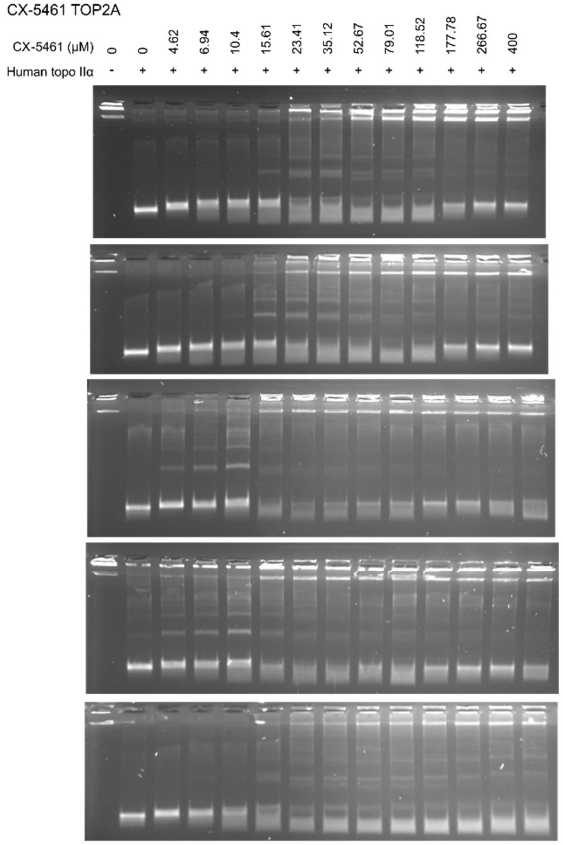

CX-5461 TOP2B

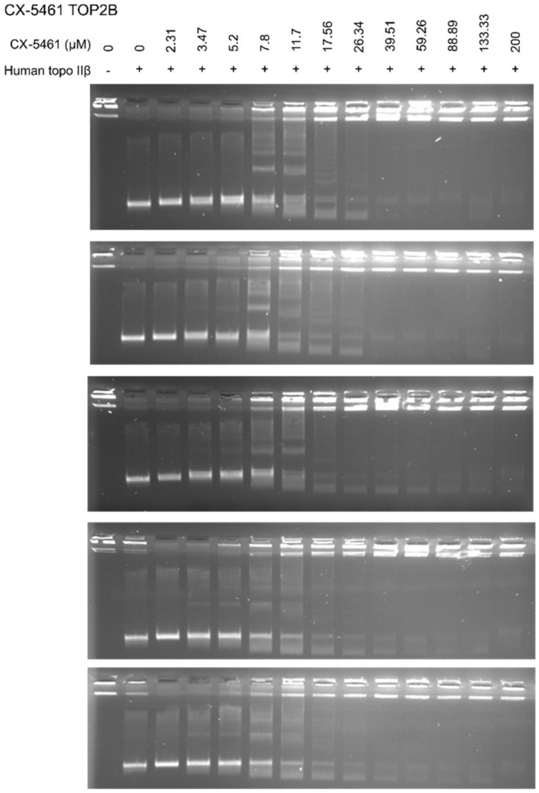

m Synthesized CX-5461 TOP2A

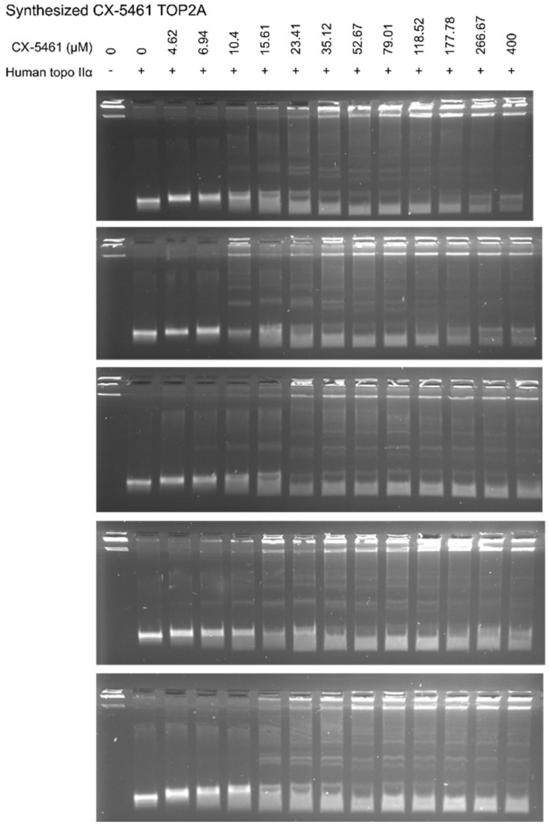

Synthesized CX-5461 TOP2B

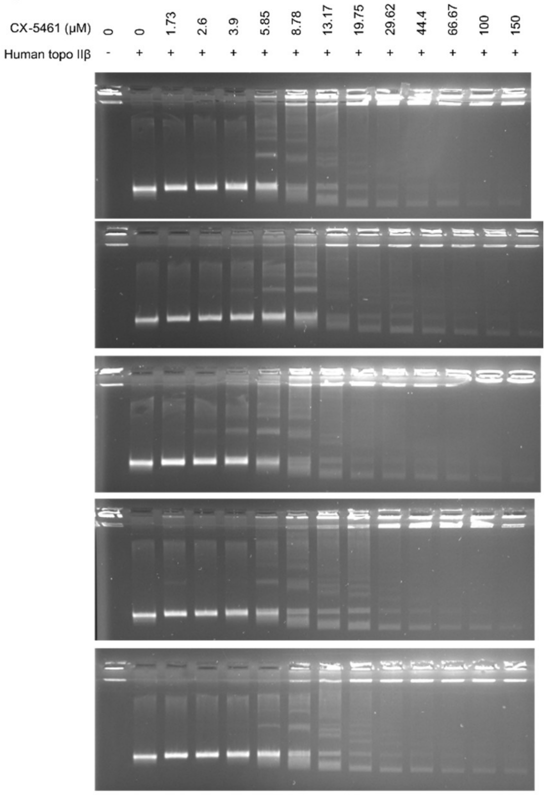

Figure S4

a. Boxplot showing the gene expression level (y-axis) of TOP2A in all n = 1,304 biologically independent DepMap cancer cell lines, 20Q2 release of these RNA-seq data. Data obtained from www.depmap.org.
b. Boxplot showing the gene expression level (y-axis) of TOP2B in all n = 1,304 biologically independent DepMap cancer cell lines, 20Q2 release of these RNA-seq data. Data obtained from http://www.depmap.org.
c. Western blots for TOP2A (left) and TOP2B (right) following shRNA knockdown in KELLY cells. Cells were transduced with TOP2A or TOP2B shRNAs (2 shRNAs for TOP2A and 3 shRNAs for TOP2B). A control shRNA was used with scramble target in human cells. Cells were cultured in medium with 0.5 µg/ml puromycin and 2 µg/ml doxycycline for 3 days, then cell lysates were collected for western blotting. β-Actin was used as a loading control. Representative results of 3 independent replicates are shown.
d. qPCR for 45S pre-rRNA expression levels (y-axis) in the same KELLY cells as used in Figure S4c. Data represent mean ± SD of n = 6 biological replicates.
e. TOP2A and TOP2B knockdown KELLY cells and negative control cells were cultured in medium with 2 µg/ml doxycycline for 3 days, then, the indicated concentration of CX-5461, etoposide, doxorubicin or cisplatin was added to the medium for 72 hours. Cell viability was determined by MTS assay. Data represent mean ± SD of n = 9 biological replicates.
f. Summarized data from all decatenation assays for the four drugs indicated, for both TOP2A (top row) and TOP2B (bottom row) assays, showing the percentage of decatenated kDNA (y-axes) against the drug concentration (x-axes). Dose-response curves were estimated using a generalized logistic regression model fit to the data using the R package “drc”, from which IC_{25}s and 95% confidence intervals were also calculated. Note that IC_{25}s were calculated because a reliable IC_{50} was not achieved within the active drug concentration range for CX-5461 in the TOP2A assays.
g. Representative gel images depicting TOP2A (left) and TOP2B (right) decatenation assay for CX-5461. CX-5461 concentrations close to IC_{25} are highlighted in red. Percentage of enzyme activity (calculated with ImageJ) is shown at the bottom of each gel. Representative results of 5 independent replicates are shown here. More replicates for CX-5461 and gel images for etoposide and doxorubicin are included in Fig. S4j-m.
h. Representative gel image from a single run of the TOP2B relaxation assay for CX-5461.
i. Schematic of CX-5461-Diazirine crosslinking and streptavidin affinity purification (left) and a representative image of TOP2B detected in the purified samples by western blot (right). Representative results of 2 independent replicates are shown.
j. TOP2A (left) and TOP2B (right) decatenation assay for etoposide. Gel images of 5 replicates are shown. For each assay, 1 U of purified human TOP2A or TOP2B protein was incubated with 200 ng of kinetoplast DNA (kDNA) with the indicated drug concentration for 30 minutes at 37°C. The product of this reaction was run on a 1% TAE gel (details in Methods). Gels were scanned (GeneGenius, Syngene, UK) and quantified using ImageJ. All 5 replicates are shown.
k. Gel images of TOP2A (left) and TOP2B (right) decatenation assay for doxorubicin. All 5 replicates are shown.

l. Gel images of TOP2A (left) and TOP2B (right) decatenation assay for commercial CX-5461. All 5 replicates are shown.
m. Gel images of TOP2A (left) and TOP2B (right) decatenation assay for the CX-5461 stock synthesized in the lab. All 5 replicates are shown.

In all boxplots, the center line represents the median, bound of box is upper and lower quartiles and whiskers are 1.5× the interquartile range. Two-tailed student's t-test was used for Fig. S4d and e. Source data for Fig. S4d-f are included in Source Data file.

a

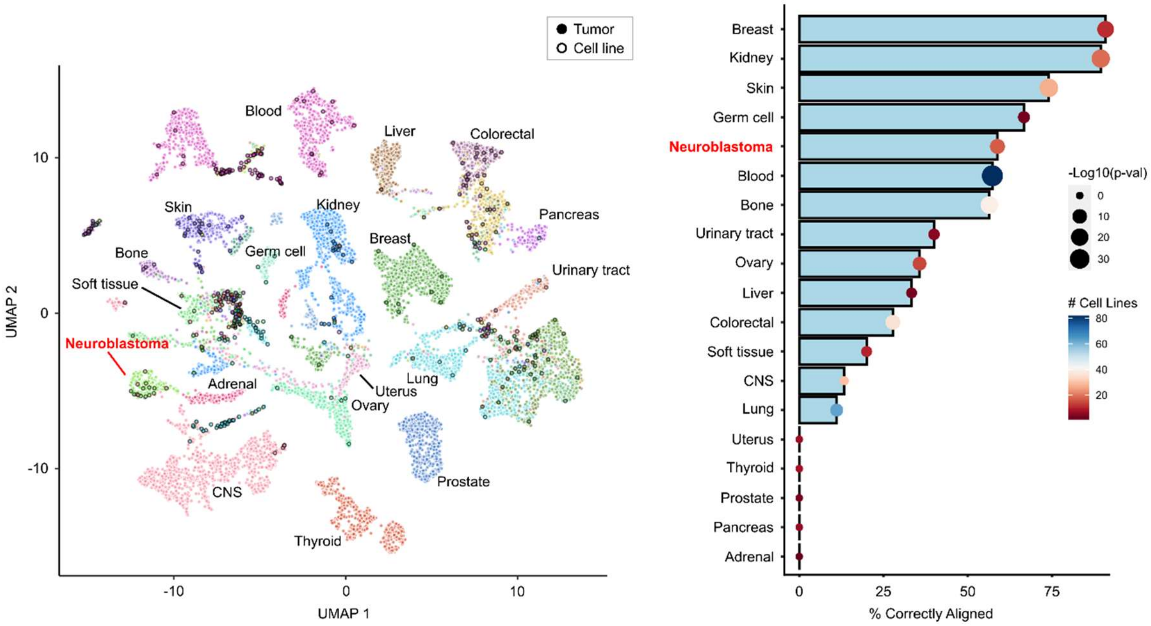

Tumor
Cell line
Blood
Liver
Colorectal
Skin
Kidney
Pancreas
Bone
Germ cell
Breast
Soft tissue
Urinary tract
Neuroblastoma
Adrenal
Uterus
Lung
Ovary
CNS
Prostate
Thyroid
UMAP 1
UMAP 2
-10
0
10
Breast
Kidney
Skin
Germ cell
Neuroblastoma
Blood
Bone
Urinary tract
Ovary
Liver
Colorectal
Soft tissue
CNS
Lung
Uterus
Thyroid
Prostate
Pancreas
Adrenal
-Log10(p-val)
Cell Lines
0
25
50
75
% Correctly Aligned

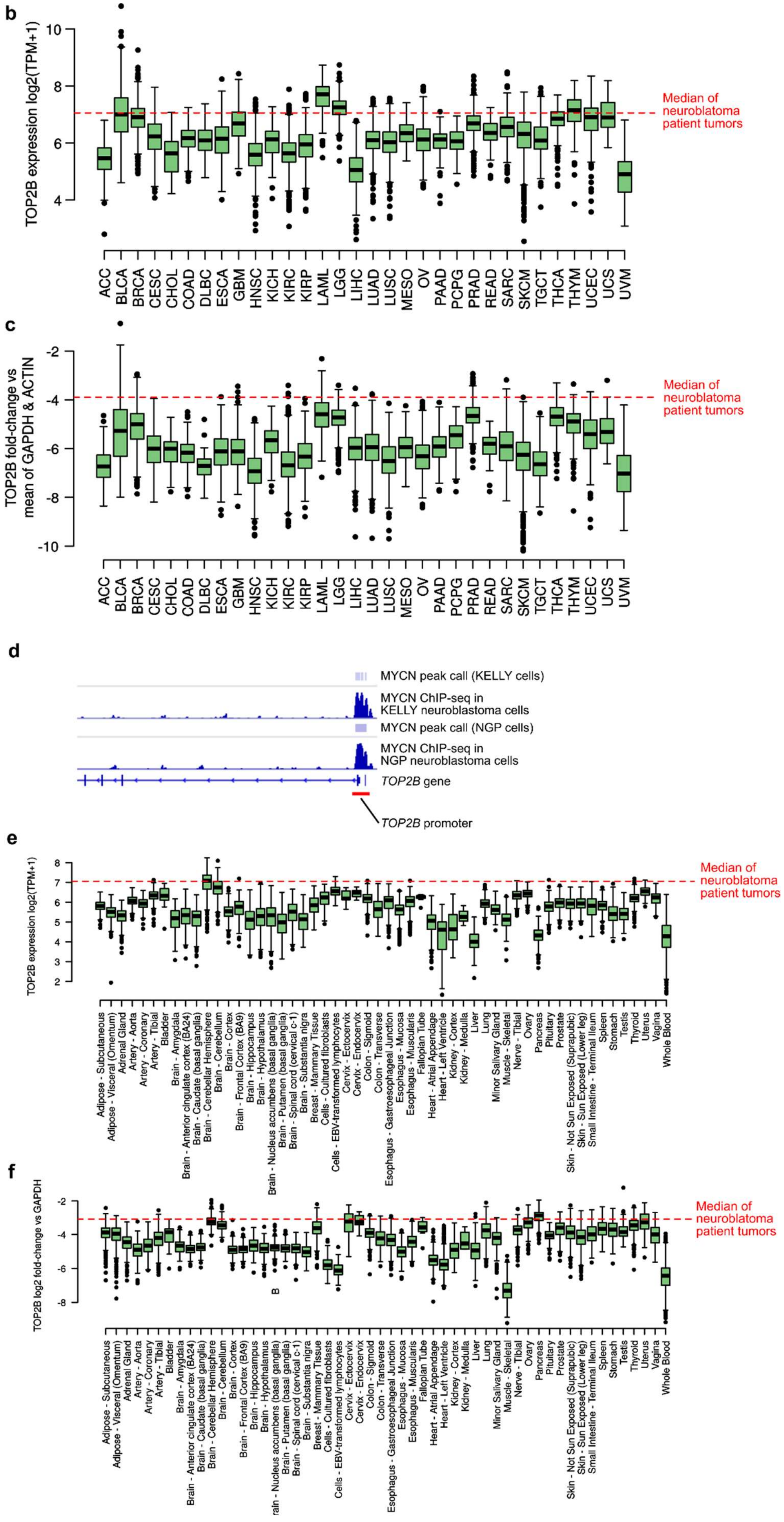
b
TOP2B expression log2(TPM+1)
10
8
6
4
Median of neuroblatoma patient tumors
ACC BLCA BRCA CESC CHOL COAD DLBC ESCA GBM HNSC KICH KIRC KIRP LAML LGG LIHC LUAD LUSC MESO OV PAAD PCPG PRAD READ SARC SKCM TGCT THCA THYM UCEC UCS UVM
c
TOP2B fold-change vs mean of GAPDH & ACTIN
-2
-4
-6
-8
-10
Median of neuroblatoma patient tumors
ACC BLCA BRCA CESC CHOL COAD DLBC ESCA GBM HNSC KICH KIRC KIRP LAML LGG LIHC LUAD LUSC MESO OV PAAD PCPG PRAD READ SARC SKCM TGCT THCA THYM UCEC UCS UVM
d
MYCN peak call (KELLY cells)
MYCN ChIP-seq in KELLY neuroblastoma cells
MYCN peak call (NGP cells)
MYCN ChIP-seq in NGP neuroblastoma cells
TOP2B gene
TOP2B promoter
e
TOP2B expression log2(TPM+1)
8
7
6
5
4
3
2
Median of neuroblatoma patient tumors
Adipose - Subcutaneous
Adipose - Visceral (Omentum)
Adrenal Gland
Artery - Aorta
Artery - Coronary
Artery - Tibial
Bladder
Brain - Amygdala
Brain - Anterior cingulate cortex (BA24)
Brain - Caudate (basal ganglia)
Brain - Cerebellar Hemisphere
Brain - Cerebellum
Brain - Cortex
Brain - Frontal Cortex (BA9)
Brain - Hippocampus
Brain - Hypothalamus
Brain - Nucleus accumbens (basal ganglia)
Brain - Putamen (basal ganglia)
Brain - Spinal cord (cervical c-1)
Brain - Substantia nigra
Breast - Mammary Tissue
Cells - Cultured fibroblasts
Cells - EBV-transformed lymphocytes
Cervix - Ectocervix
Cervix - Endocervix
Colon - Sigmoid
Colon - Transverse
Esophagus - Gastroesophageal Junction
Esophagus - Mucosa
Esophagus - Muscularis
Fallopian Tube
Heart - Atrial Appendage
Heart - Left Ventricle
Kidney - Cortex
Kidney - Medulla
Liver
Lung
Minor Salivary Gland
Muscle - Skeletal
Nerve - Tibial
Ovary
Pancreas
Pituitary
Prostate
Skin - Not Sun Exposed (Suprapubic)
Skin - Sun Exposed (Lower leg)
Small Intestine - Terminal Ileum
Spleen
Stomach
Testis
Thyroid
Uterus
Vagina
Whole Blood
f
TOP2B log2 fold-change vs GAPDH
-2
-4
-6
-8
Median of neuroblatoma patient tumors
B
Adipose - Subcutaneous
Adipose - Visceral (Omentum)
Adrenal Gland
Artery - Aorta
Artery - Coronary
Artery - Tibial
Bladder
Brain - Amygdala
Brain - Anterior cingulate cortex (BA24)
Brain - Caudate (basal ganglia)
Brain - Cerebellar Hemisphere
Brain - Cerebellum
Brain - Cortex
Brain - Frontal Cortex (BA9)
Brain - Hippocampus
Brain - Hypothalamus
rain - Nucleus accumbens (basal ganglia)
Brain - Putamen (basal ganglia)
Brain - Spinal cord (cervical c-1)
Brain - Substantia nigra
Breast - Mammary Tissue
Cells - Cultured fibroblasts
Cells - EBV-transformed lymphocytes
Cervix - Ectocervix
Cervix - Endocervix
Colon - Sigmoid
Colon - Transverse
Esophagus - Gastroesophageal Junction
Esophagus - Mucosa
Esophagus - Muscularis
Fallopian Tube
Heart - Atrial Appendage
Heart - Left Ventricle
Kidney - Cortex
Kidney - Medulla
Liver
Lung
Minor Salivary Gland
Muscle - Skeletal
Nerve - Tibial
Ovary
Pancreas
Pituitary
Prostate
Skin - Not Sun Exposed (Suprapubic)
Skin - Sun Exposed (Lower leg)
Small Intestine - Terminal Ileum
Spleen
Stomach
Testis
Thyroid
Uterus
Vagina
Whole Blood

g

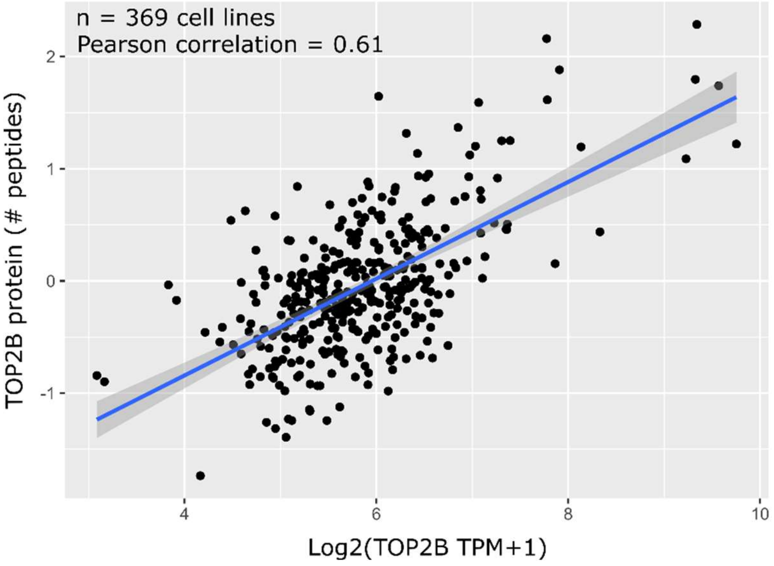

Figure S5

a. UMAP representation showing the genome-wide gene-expression-based alignment of all GDSC (Sanger) cell lines with patient tumor gene expression data from TCGA, Treehouse and TARGET, generated using the Celligner tool. The points representing individual cell lines have a black border. Points have been colored by their lineage and clusters have been labelled by tumor lineage. The bar plot (right panel) shows the proportion of cell lines from a given lineage that correctly align with the appropriate patient tumor cluster (x-axis). The points have been colored by the number of cell lines in the datasets. The size of the points have been scaled by the *P*-value obtained from a Fisher's exact test for cluster membership.
b. The median *TOP2B* $\log_2$(TPM+1) normalized expression from 88 neuroblastoma patient tumors compared to all n = 9,968 biologically independent TCGA lineages.
c. The same data as Fig. S5b but with *TOP2B* normalized to *ACTIN* and *GAPDH* housekeeping genes.
d. ChIP-seq data for MYCN at the TOP2B locus and promoter. Data shown from NGP and KELLY neuroblastoma cell lines. This figure was created from published ChIP-seq data generated by Zeid *et al.* (Nature Genetics 2018).
e. The median *TOP2B* $\log_2$(TPM+1) normalized expression from 88 neuroblastoma patient tumors compared to all GTEx tissue types. GTEx data include n = 57 tissues types obtained from n = 948 biologically independent donors.
f. The same data as Fig. S5e but with *TOP2B* normalized to *GAPDH* housekeeping genes (note that *ACTIN* expression was not used as a negative control here as it has variable expression across GTEx normal tissues).
g. Correlation of TOP2B mRNA and protein levels across 369 cell lines. mRNA expression is measured in Log2 of transcripts per million (TPM) + 1. Protein expression is measured in normalized number of peptides. Cell line data were obtained from https://www.depmap.org.

In all boxplots, the center line represents the median, bound of box is upper and lower quartiles and whiskers are 1.5× the interquartile range.

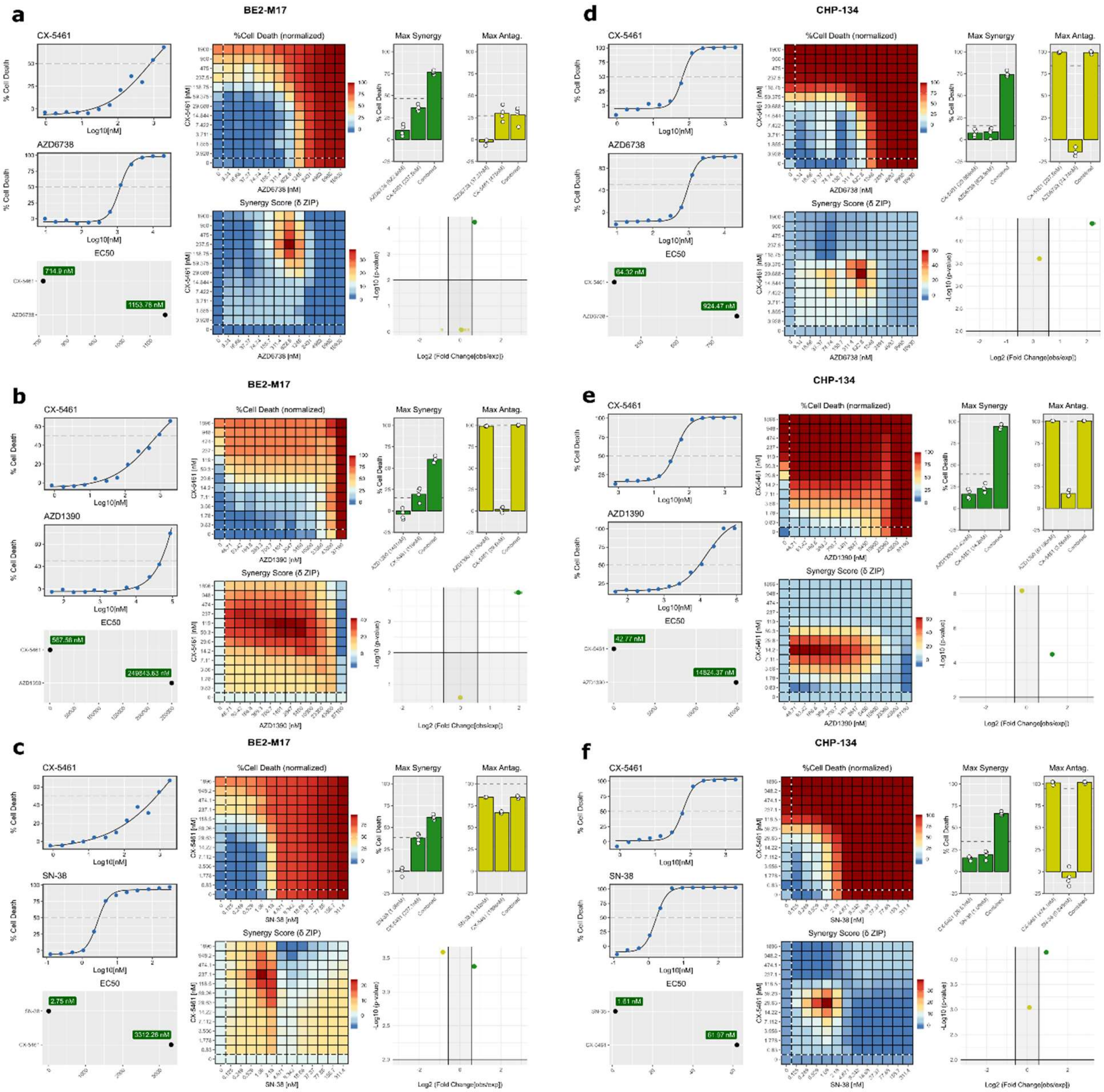
a
BE2-M17
d
CHP-134
b
BE2-M17
e
CHP-134
c
BE2-M17
f
CHP-134
CX-5461
AZD6738
AZD1390
SN-38
EC50
%Cell Death (normalized)
Synergy Score (δ ZIP)
Max Synergy
Max Antag.
% Cell Death
Log10[nM]
-Log10 (p-value)
Log2 (Fold Change[obs/exp])
714.9 nM
1153.78 nM
64.32 nM
924.47 nM
567.56 nM
249843.63 nM
42.77 nM
14824.37 nM
2.75 nM
3312.26 nM
1.61 nM
61.97 nM

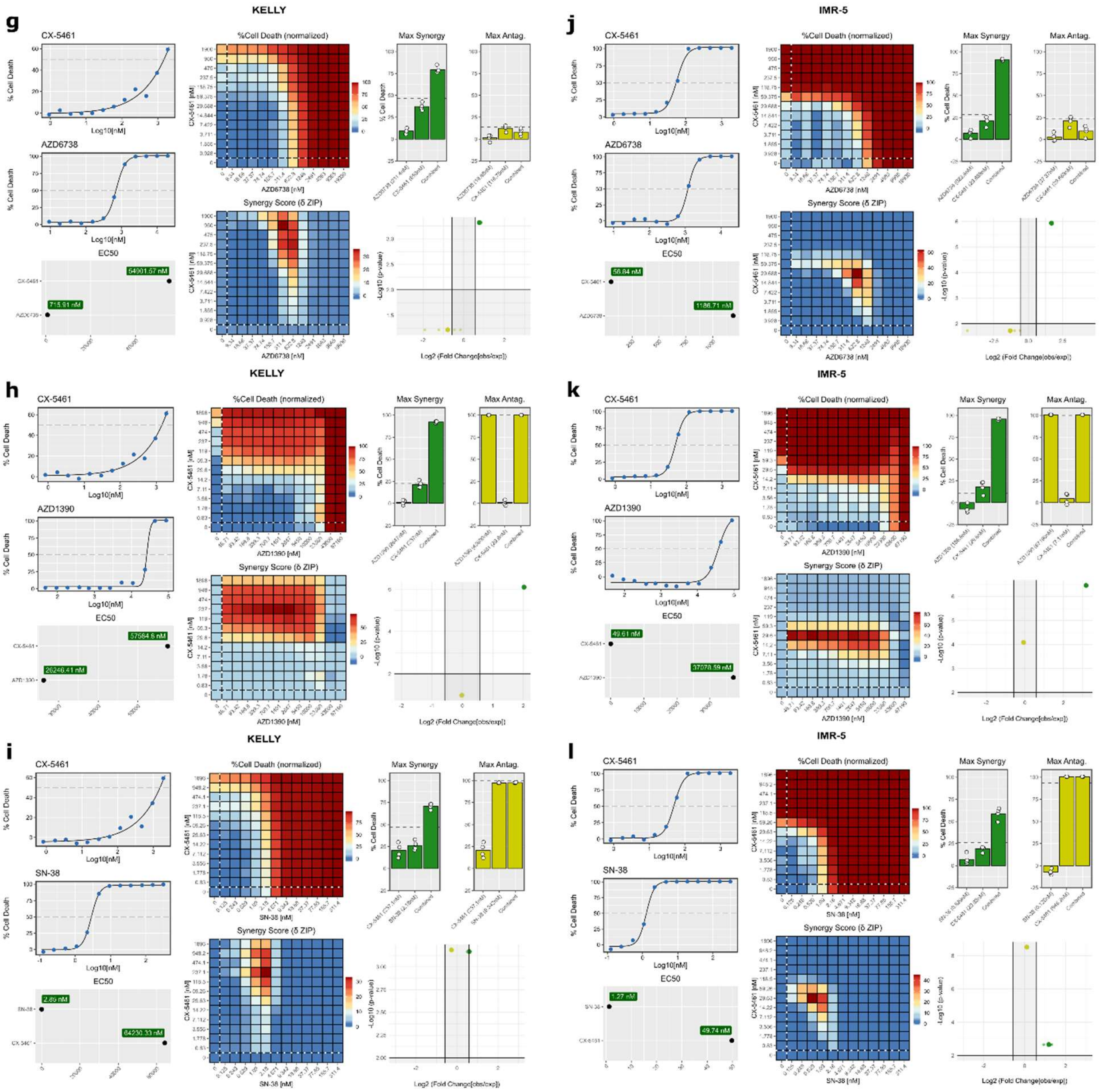
g
KELLY
CX-5461
AZD6738
EC50
715.91 nM
64901.57 nM
%Cell Death (normalized)
Synergy Score (δ ZIP)
Max Synergy
Max Antag.
j
IMR-5
58.84 nM
1186.71 nM
h
AZD1390
57584.6 nM
26246.41 nM
k
49.61 nM
37078.59 nM
i
SN-38
2.89 nM
64230.33 nM
l
1.27 nM
49.74 nM
Log10[nM]
% Cell Death
Log2 (Fold Change[obs/exp])
-Log10 (p-value)

m

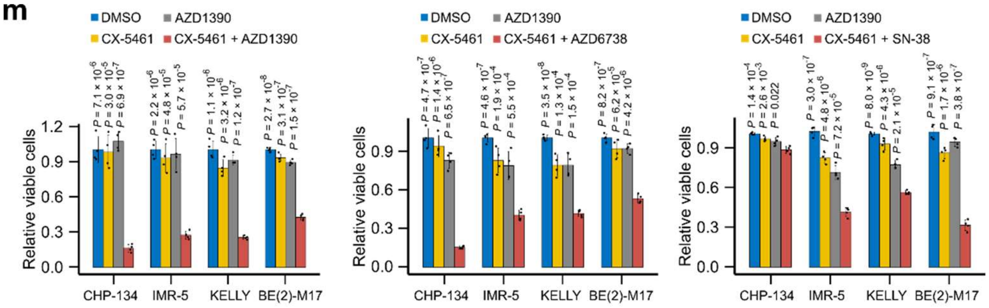

	Concentration (µM) CX-5461	AZD1390	Time (day)
CHP-134	0.01	0.1	3
IMR-5	0.01	0.5	4
KELLY	0.04	0.5	4
BE(2)-M17	0.04	0.5	4

	Concentration (µM) CX-5461	AZD6738	Time (day)
CHP-134	0.02	0.6	4
IMR-5	0.01	0.4	4
KELLY	0.04	0.3	6
BE(2)-M17	0.06	0.8	6

	Concentration CX-5461 (µM)	SN-38 (nM)	Time (day)
CHP-134	0.006	0.2	5
IMR-5	0.01	0.25	4
KELLY	0.02	0.5	5
BE(2)-M17	0.06	0.7	5

n

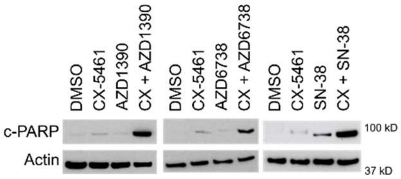

o

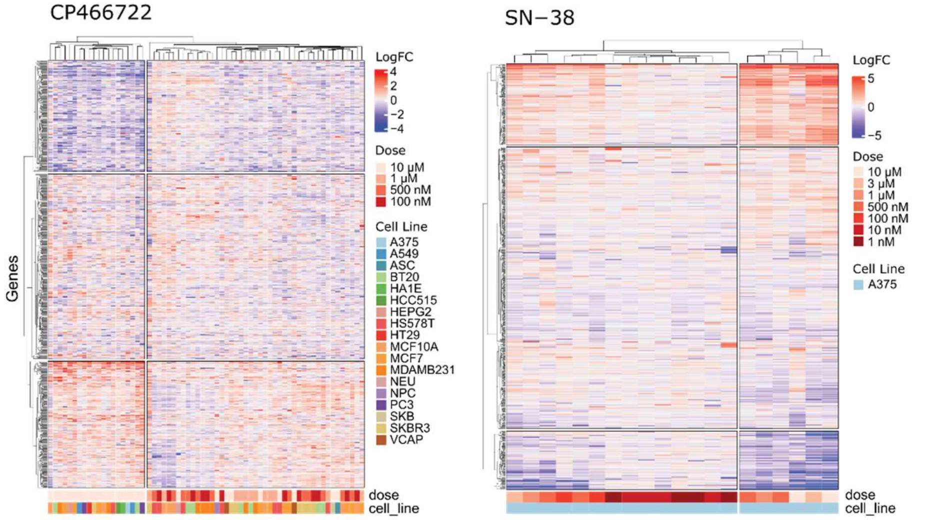

MSigDB Hallmark 2020: CP466722			
Term	Overlap	P.value	Adj. P.value
Apoptosis	21/161	6.90e-12	3.24e-10
Fatty Acid Metabolism	20.158	3.84e-11	9.02e-10
Coagulation	16/138	1.28e-08	2.01e-07
Complement	18/200	8.55e-08	1.00e-06
Allograft Rejection	17/200	4.43e07	4.16e-06

MSigDB Hallmark 2020: SN-38			
Term	Overlap	P.value	Adj. P.value
Apoptosis	16/61	1.01e-10	4.77e-09
Complement	14/200	1.37e-07	3.21e-06
Fatty Acid Metabolism	13/200	8.98e-07	1.41e-05
Xenobiotic Metabolism	12/200	5.45e-06	6.39e-05
Allograft Rejection	9/158	1.23e-06	1.15e-03

Figure S6

a-c. Drug screening with combination of CX-5461 and AZD-6738 (a), AZD-1390 (b), or SN-38 (c) in BE(2)-M17 cells. Dose-response curves and extrapolated EC_{50} are shown on the left. Heatmap matrices (middle) represent percent cell death (upper) and calculated synergy scores (lower) of the combination treatment. Bar plots (upper right) represent the maximum synergy (green) and maximum antagonism (yellow) scores corresponding to the synergy matrix. White dots represent four independent experiments corresponding to these maxima. Volcano plot (lower right) represents the $\log_2$ fold change (x-axis) of the observed vs. expected (based on an additive model) values and

P-values derived from one-sample t-tests (y-axis). Larger opaque dots represent the average of four independent experiments, while the smaller translucent dots correspond to the individual replicates.

d-f. Same as (a), (b), (c) respectively but in CHP-134 cells.

g-i. Same as (a), (b), (c) respectively but in KELLY cells.

j-l. Same as (a), (b), (c) respectively but in IMR-5 cells.

m. Synergy validation with MTS assays for CX-5461 in combination with AZD6738, AZD1390, or SN-38. Four cell lines were tested. Drug concentrations and time of treatment are listed at the bottom. Data represent mean ± SD of n = 4 biological replicates. *P* values are from two-tailed student's t-test. The source data are included in Source Data file.

n. Western blots for cleaved PARP (c-PARP) in CHP-134 cells treated with CX-5461 in combination with AZD6738, AZD1390, or SN-38. The cells were treated for 48 h. Drug concentrations for CX-5461 + AZD1390 and CX-5461 + AZD6738 combinations are the same as in Fig. S6m. For CX-5461 + SN-38 combination, CX-5461 = 0.03 μM; SN-38 = 1 nM. β-Actin was used as a loading control. Representative results of 2 independent replicates are shown.

o. Gene expression analysis of *ATM* and *TOP1* inhibitors in the Connectivity Map (CMAP) dataset. Large scale drug screening data from the CMAP portal was analyzed to determine the transcriptional response of single agent inhibitors of *ATM* (CP466722) and *TOP1* (SN-38). LogFC values, representing relative change to DMSO controls, were retrieved for each gene. The CMAP experimental design consists of the measurement of a panel of genes across cell lines and drug concentrations. The resulting data is then used as input for imputation models to estimate the response across all other genes. The analysis described here was restricted to only those genes directly assayed. Hierarchical clustering of logFC values identified groups of differentially expressed genes that were elicited from these compounds in a dose-dependent manner (CP466722, left column; SN-38, right column). Gene ontology analyses of these genes (bottom tables) queried against the MSigDB Hallmark 2020 gene set database revealed an expected drug-induced apoptotic program that was consistent between the two inhibitors.

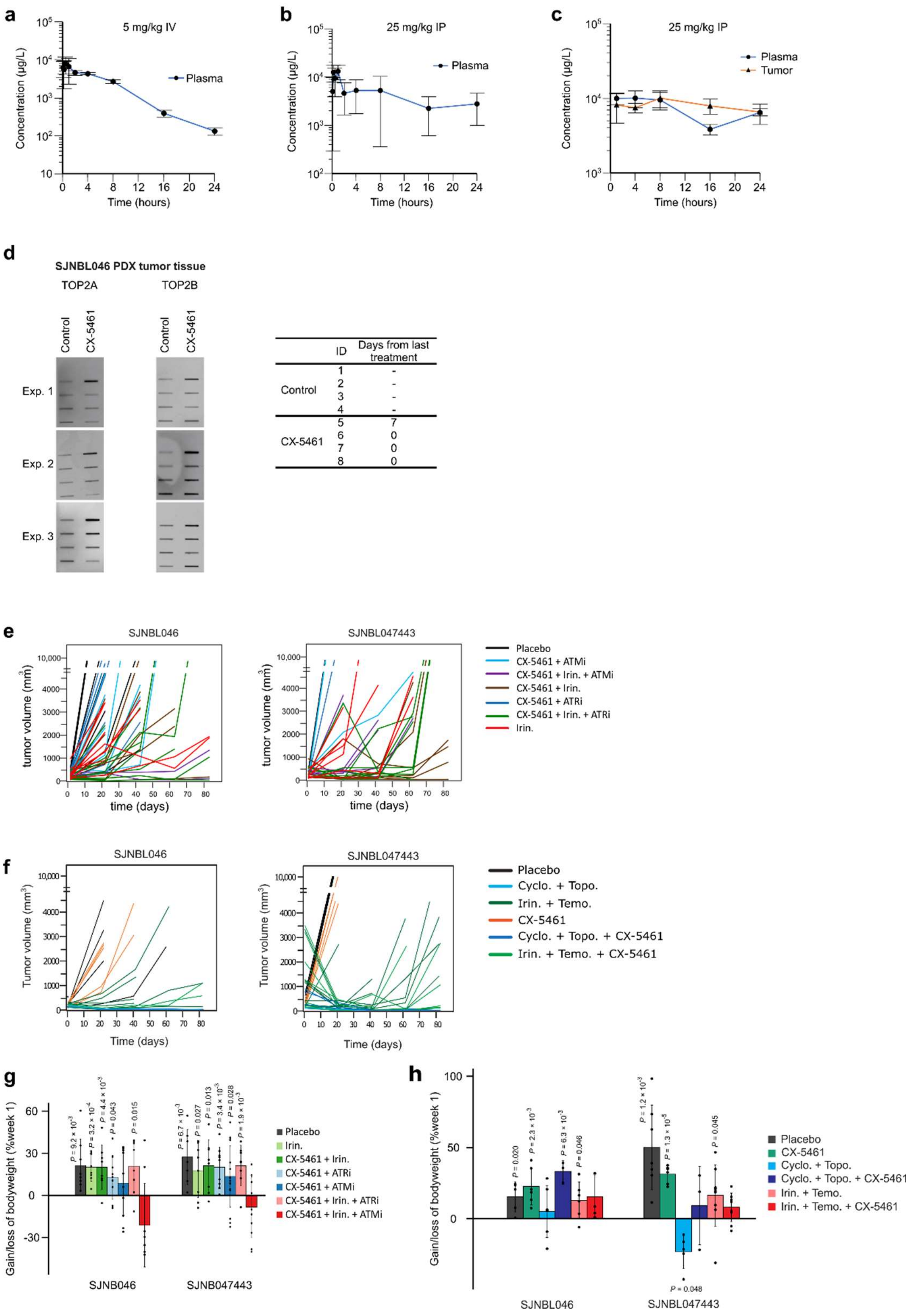
a
5 mg/kg IV
Plasma
Concentration (μg/L)
Time (hours)
b
25 mg/kg IP
Plasma
Concentration (μg/L)
Time (hours)
c
25 mg/kg IP
Plasma
Tumor
Concentration (μg/L)
Time (hours)
d
SJNBL046 PDX tumor tissue
TOP2A
TOP2B
Control
CX-5461
Exp. 1
Exp. 2
Exp. 3
ID
Days from last treatment
Control
1 -
2 -
3 -
4 -
CX-5461
5 7
6 0
7 0
8 0
e
SJNBL046
SJNBL047443
tumor volume (mm3)
time (days)
Placebo
CX-5461 + ATMi
CX-5461 + Irin. + ATMi
CX-5461 + Irin.
CX-5461 + ATRi
CX-5461 + Irin. + ATRi
Irin.
f
SJNBL046
SJNBL047443
Tumor volume (mm3)
Time (days)
Placebo
Cyclo. + Topo.
Irin. + Temo.
CX-5461
Cyclo. + Topo. + CX-5461
Irin. + Temo. + CX-5461
g
Gain/loss of bodyweight (%week 1)
SJNB046
SJNB047443
Placebo
Irin.
CX-5461 + Irin.
CX-5461 + ATRi
CX-5461 + ATMi
CX-5461 + Irin. + ATRi
CX-5461 + Irin. + ATMi
h
Gain/loss of bodyweight (%week 1)
SJNBL046
SJNBL047443
Placebo
CX-5461
Cyclo. + Topo.
Cyclo. + Topo. + CX-5461
Irin. + Temo.
Irin. + Temo. + CX-5461

Figure S7

a. Plasma concentration (y-axis) of CX-5461 achieved in athymic nude mice over 24 hours (x-axis) following a 5 mg/kg intravenous (IV) injection of CX-5461 (n = 3 mice at each time point). Data represent mean ± SD.
b. Same as (A) but mice were treated with 25 mg/kg CX-5461 via intraperitoneal (IP) injection.
c. Plasma and tumor concentrations (y-axis) of CX-5461 achieved in athymic nude mice over 24 hours (x-axis) following a 25 mg/kg IP injection of CX-5461 (n = 3 mice at each time point). Data represent mean ± SD.
d. Raw images for data in Figure 7A. The table (right) shows the duration between the harvesting of each tumor and the previous drug treatment for each mouse.
e. Tumor volume (y-axis) measured by ultrasound in SJNBL046 (left) and SJNBL047443 (right) PDX mice in study 1. Curves are colored by treatment group.
f. Tumor volume (y-axis) measured by ultrasound in SJNBL046 (left) and SJNBL047443 (right) PDX mice in study 2. Curves are colored by treatment group.

g-h. Bar plot showing % change of bodyweight at death for all mice in each group (after completion of therapy, body weight of all mice survived were monitored till death) in study 1 (g) and study 2 (h). Data represent the mean ± SD of the bodyweight in each group. Number of mice in each group can be different. These numbers are provided in Source Data file. *P* values are from two-tailed student's t-test.Source data for Fig. S7a-c, g and h are included in Source Data file.

a

Gating strage for Figure 3a-c and Figure S3a

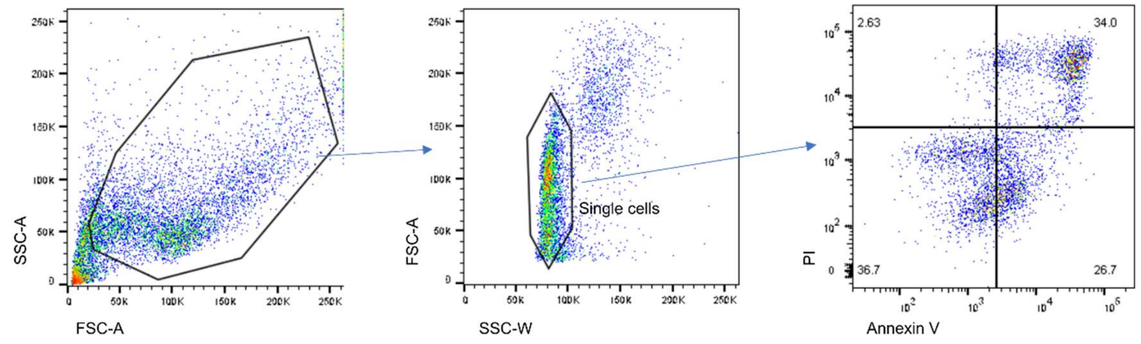

b

Gating strage for Figure 3g

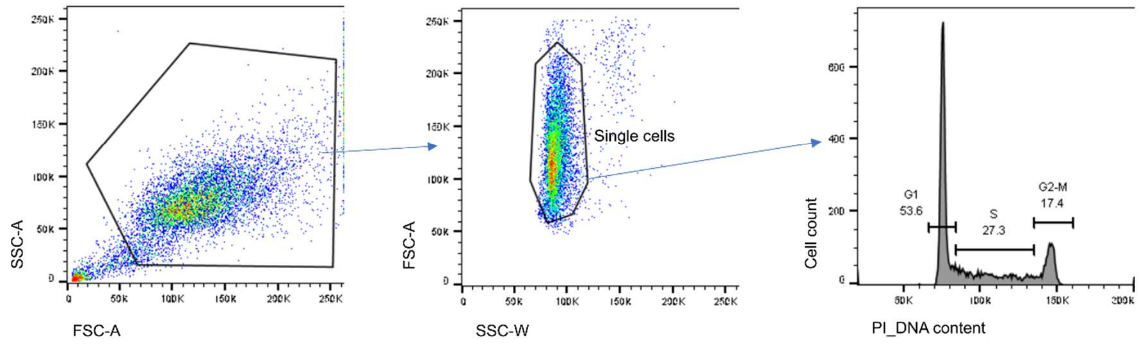

c

Gating strage for Figure S3i

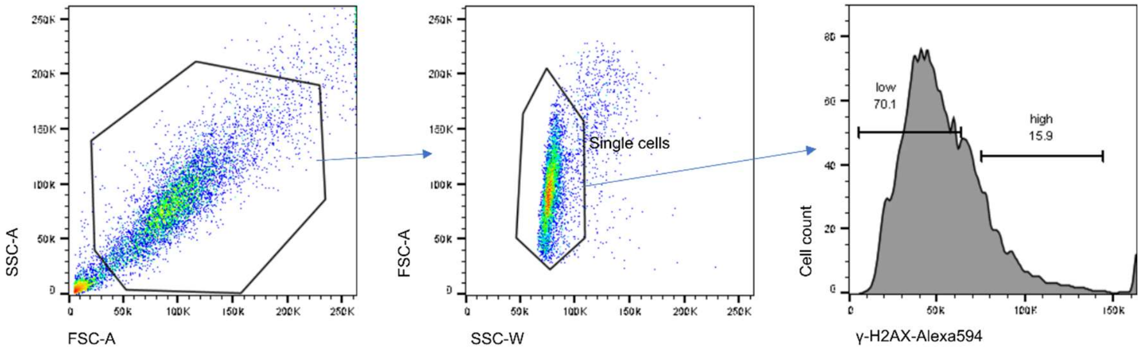

Figure S8

a. Flow cytometry gating strategy for Figure 3a-c and Figure S3a.

b. Flow cytometry gating strategy for Figure 3g.

c. Flow cytometry gating strategy for Figure S3i.

Figure S9 (page 26-50). Unprocessed images for western blotting and band depletion assay.

Figure 1g

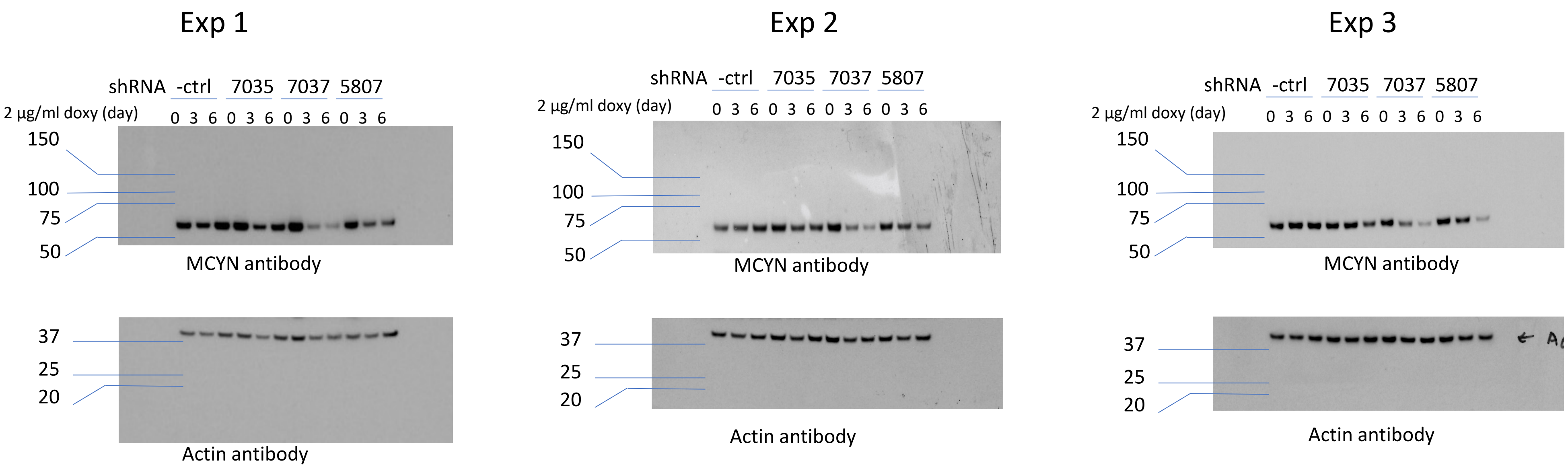
Exp 1
shRNA -ctrl 7035 7037 5807
2 µg/ml doxy (day) 0 3 6 0 3 6 0 3 6 0 3 6
150
100
75
50
MCYN antibody
37
25
20
Actin antibody
Exp 2
shRNA -ctrl 7035 7037 5807
2 µg/ml doxy (day) 0 3 6 0 3 6 0 3 6 0 3 6
150
100
75
50
MCYN antibody
37
25
20
Actin antibody
Exp 3
shRNA -ctrl 7035 7037 5807
2 µg/ml doxy (day) 0 3 6 0 3 6 0 3 6 0 3 6
150
100
75
50
MCYN antibody
37
25
20
Actin antibody

Figure 2k and S2g

Exp 1

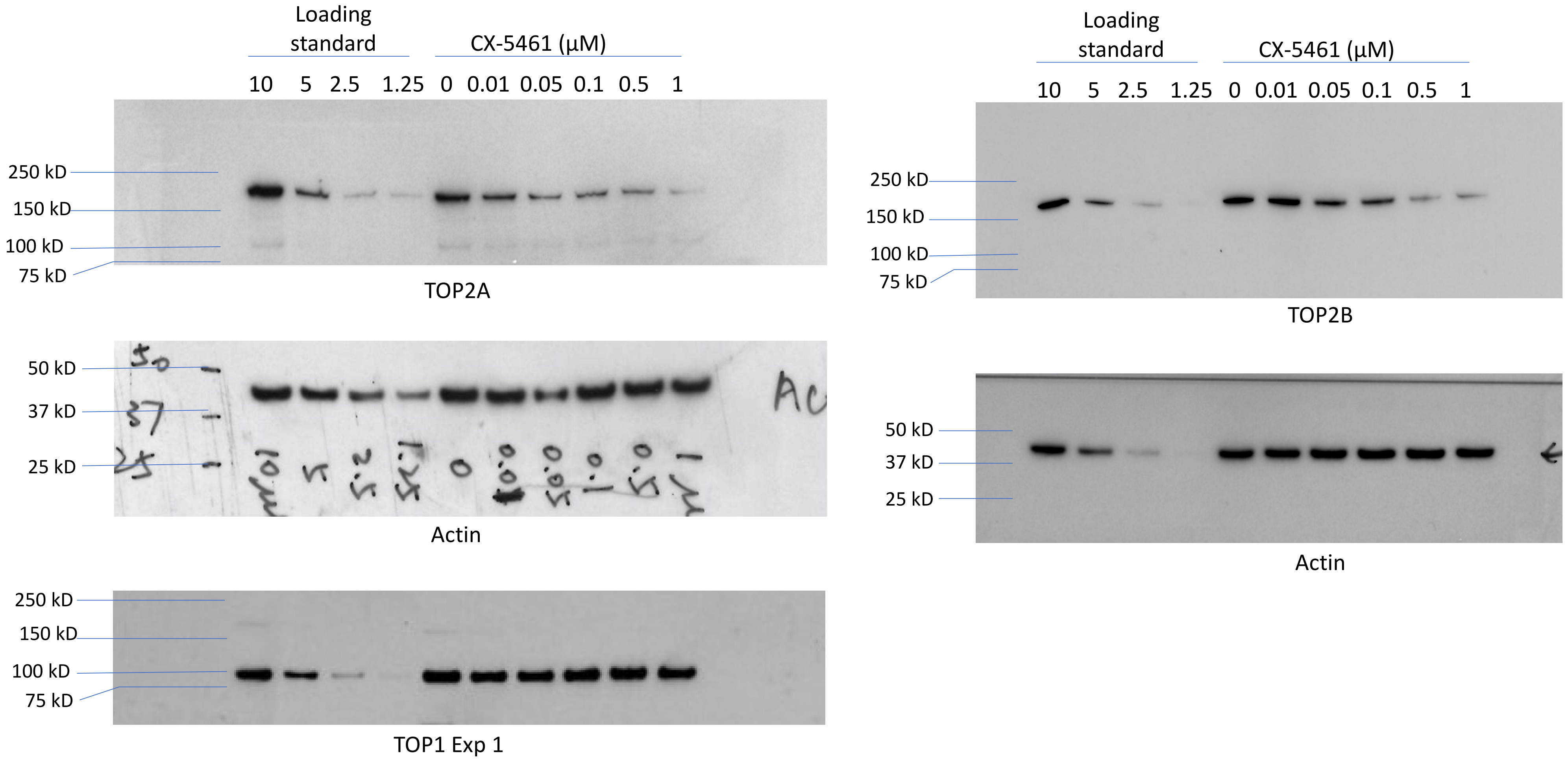

Figure 2k and S2g

Exp 2

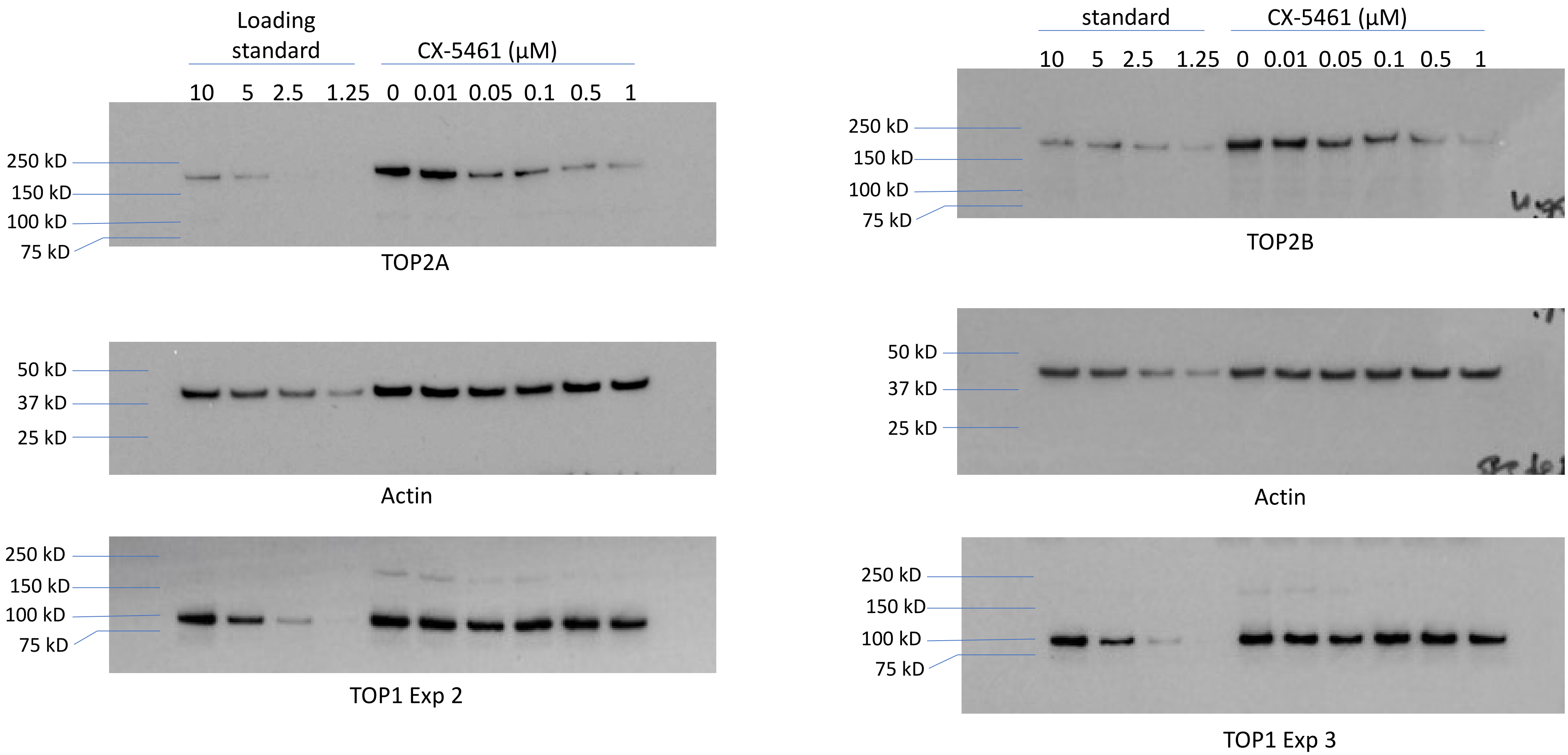

Figure 2k and S2g

Exp 3

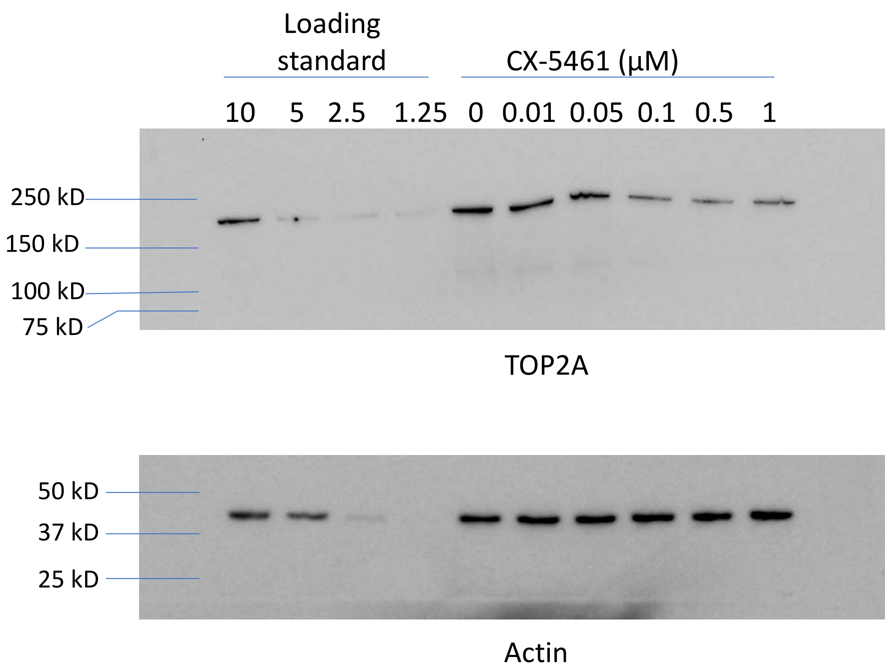

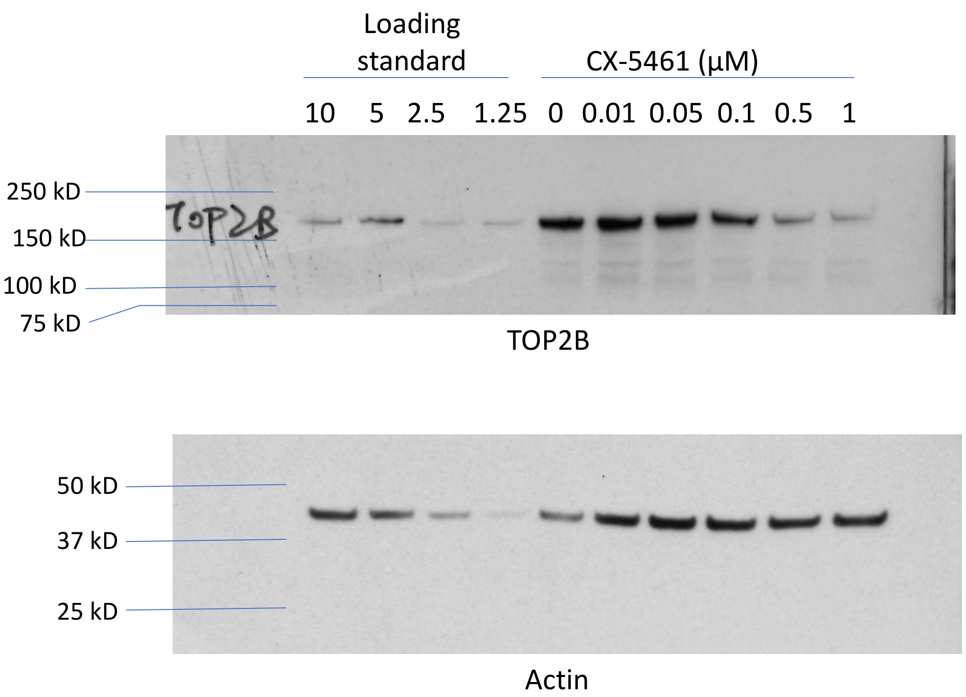

Figure 2l and S2g

Exp 1

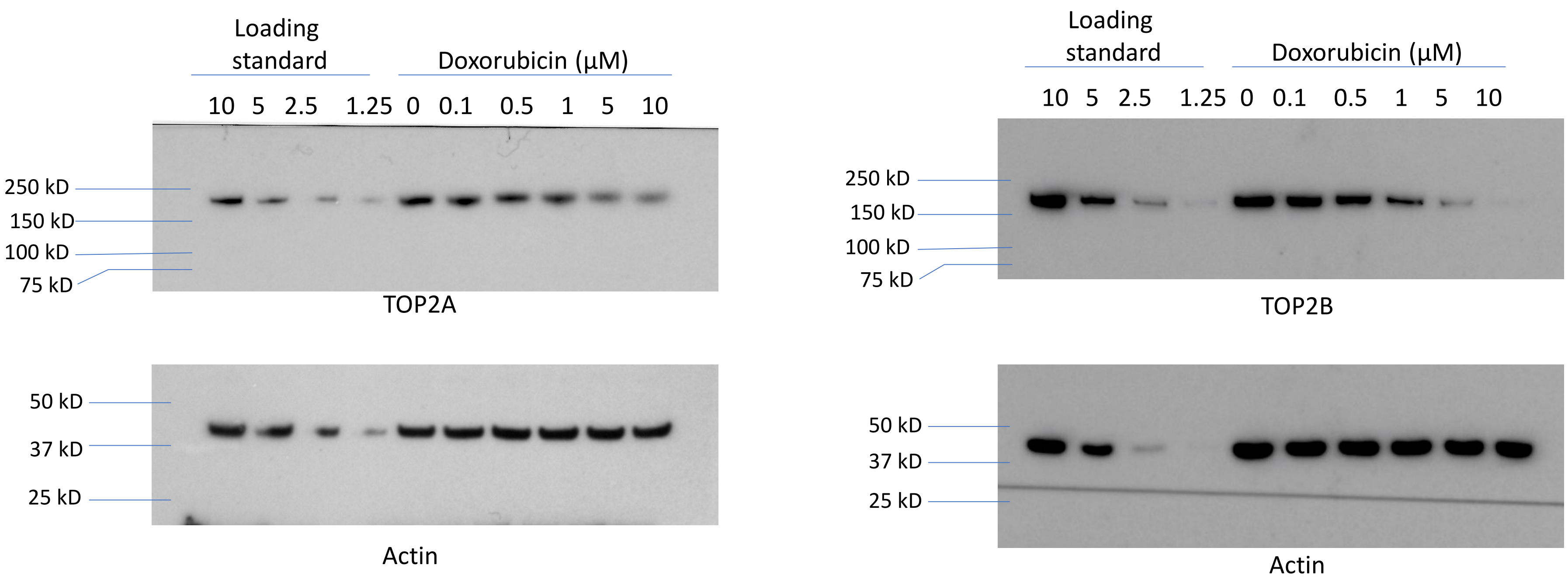

Figure 2l and S2g

Exp 2

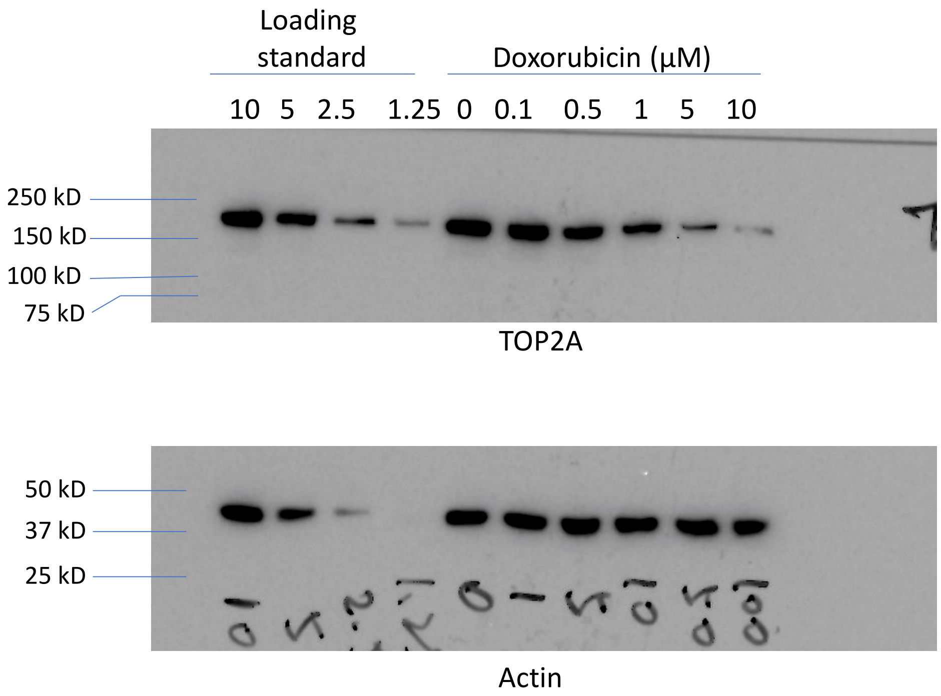

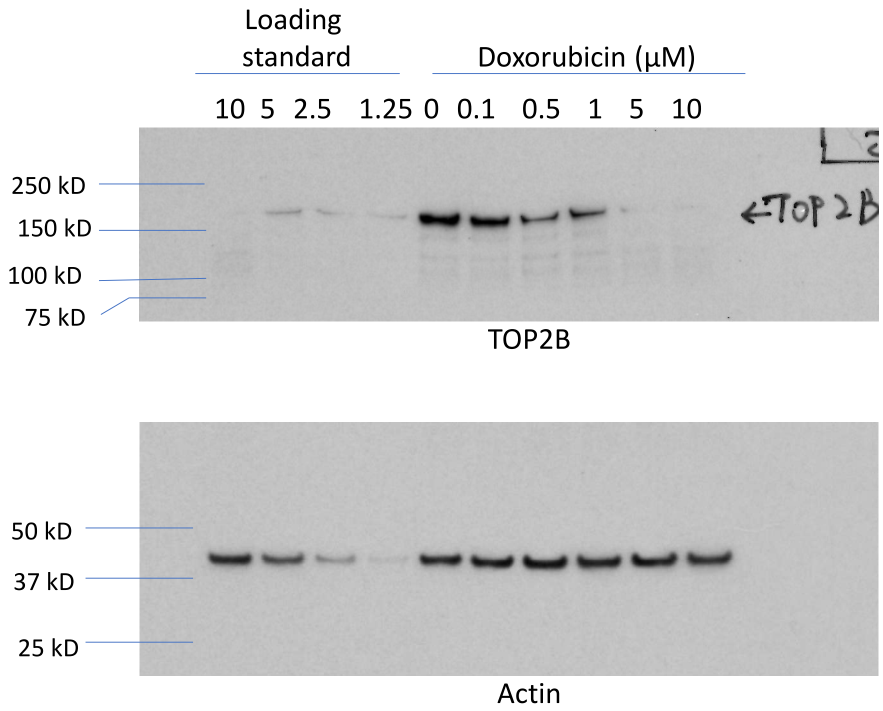

Figure 2l and S2g

Exp 3

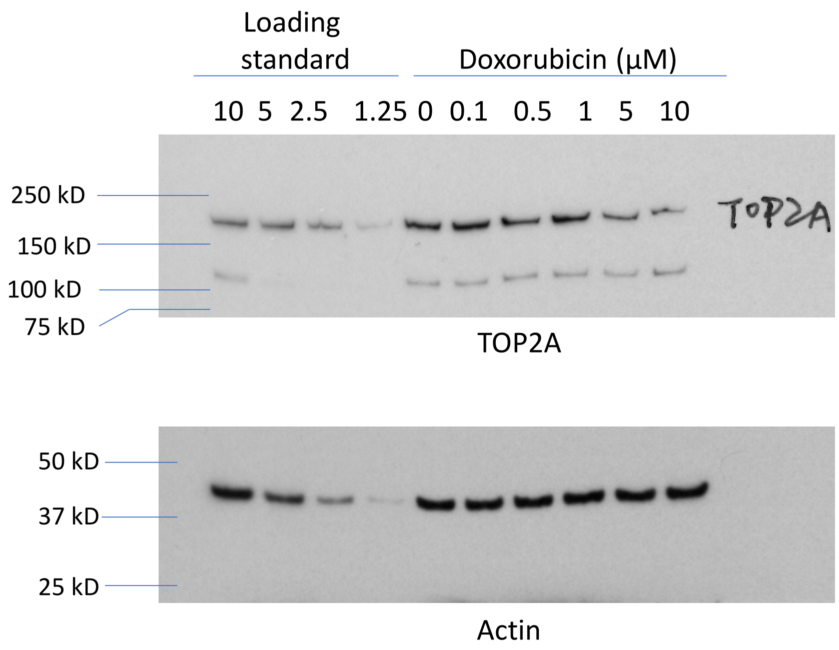

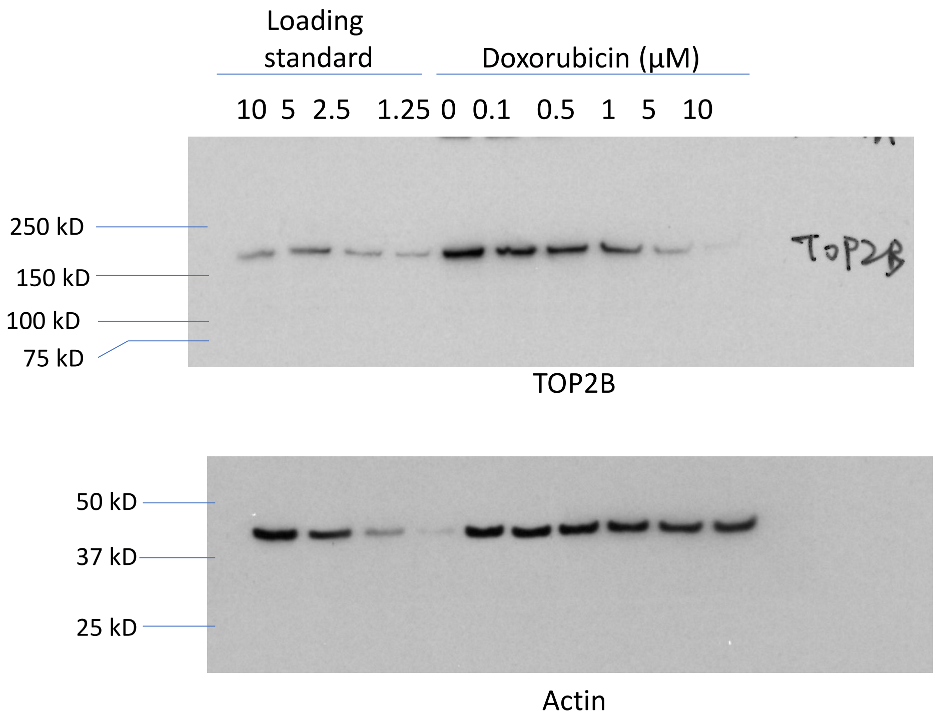

Figure 2m

Exp 1

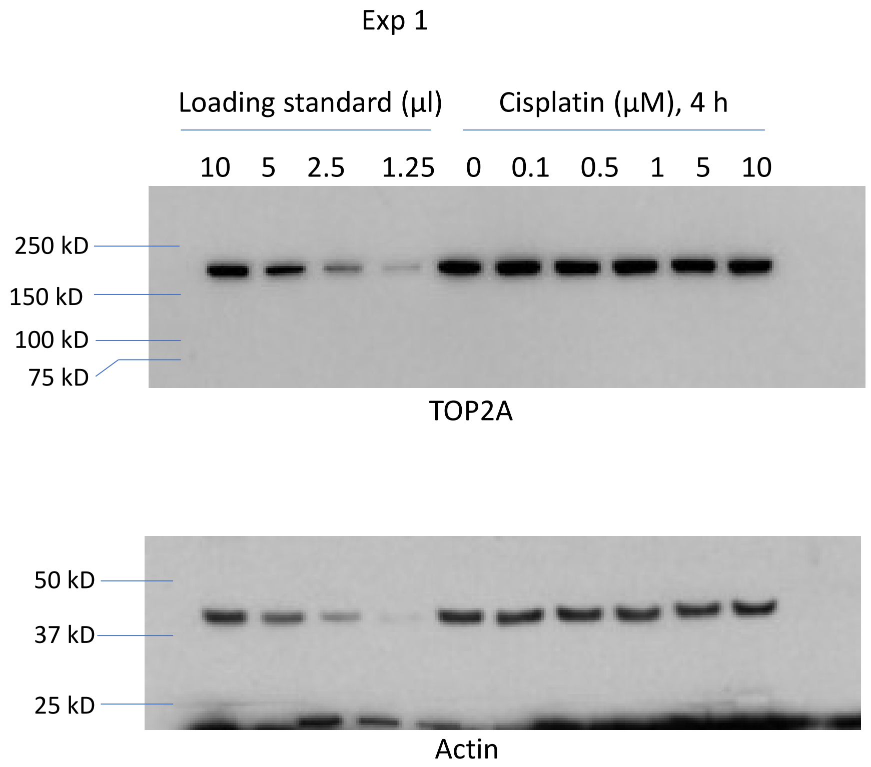

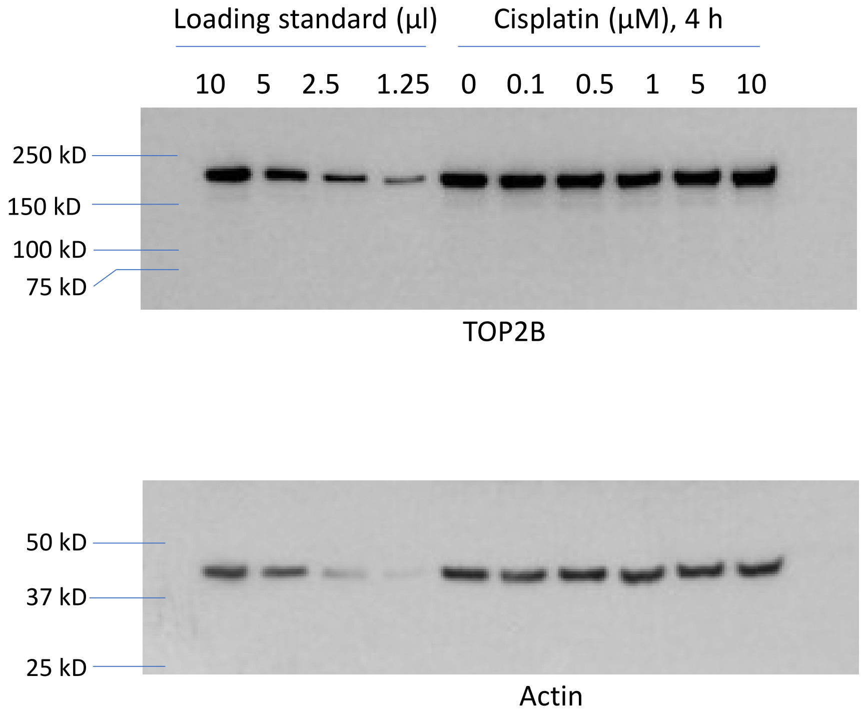

Figure 2m

Exp 2

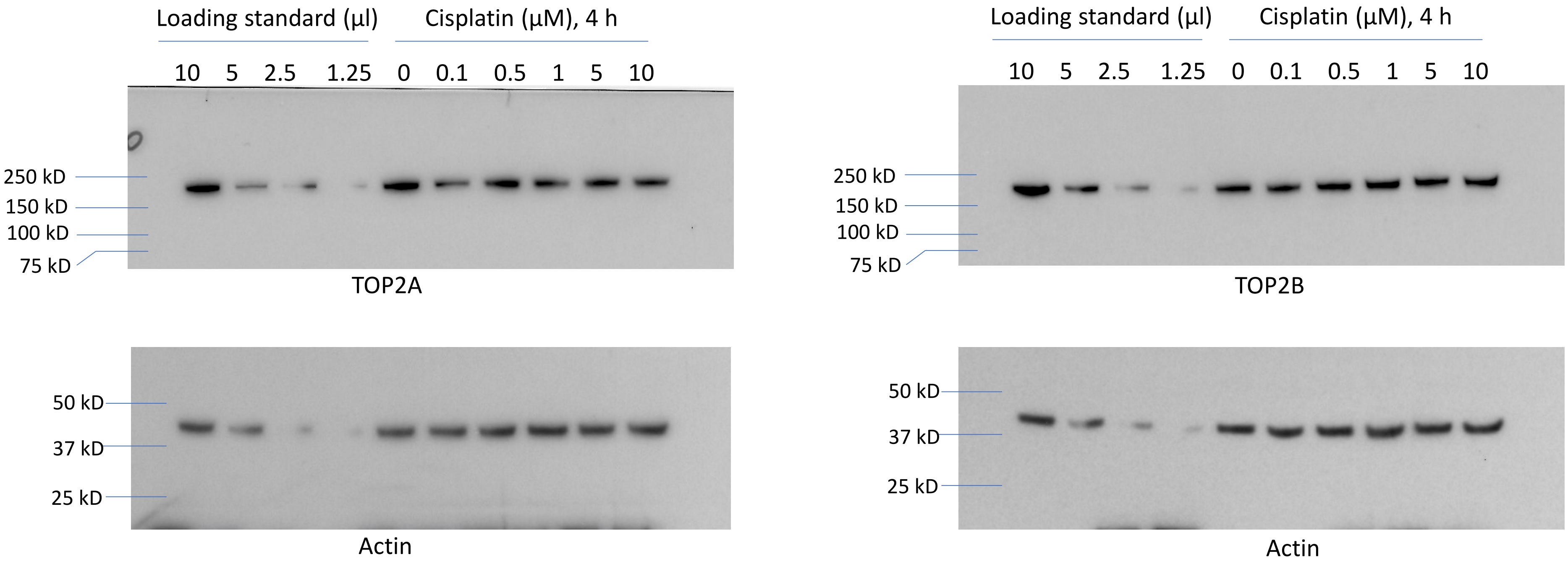

Figure 2m

Exp 3

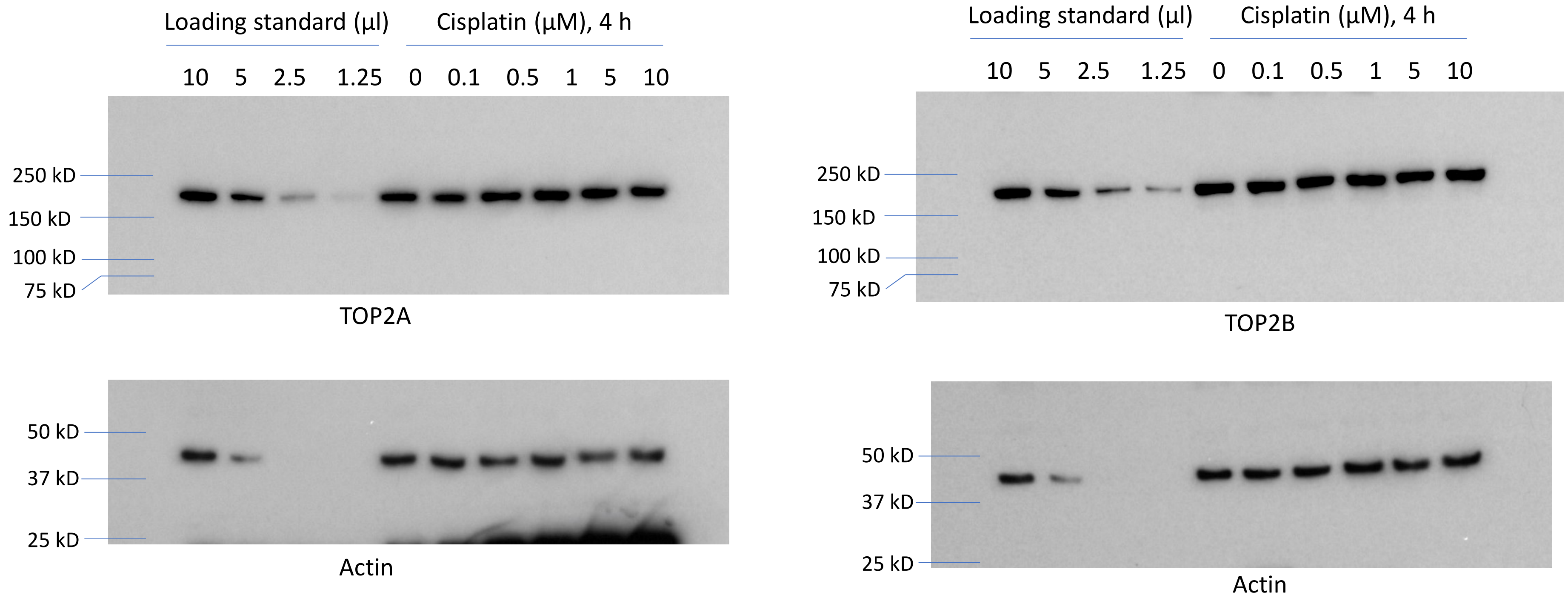

Figure 3d

Exp 1

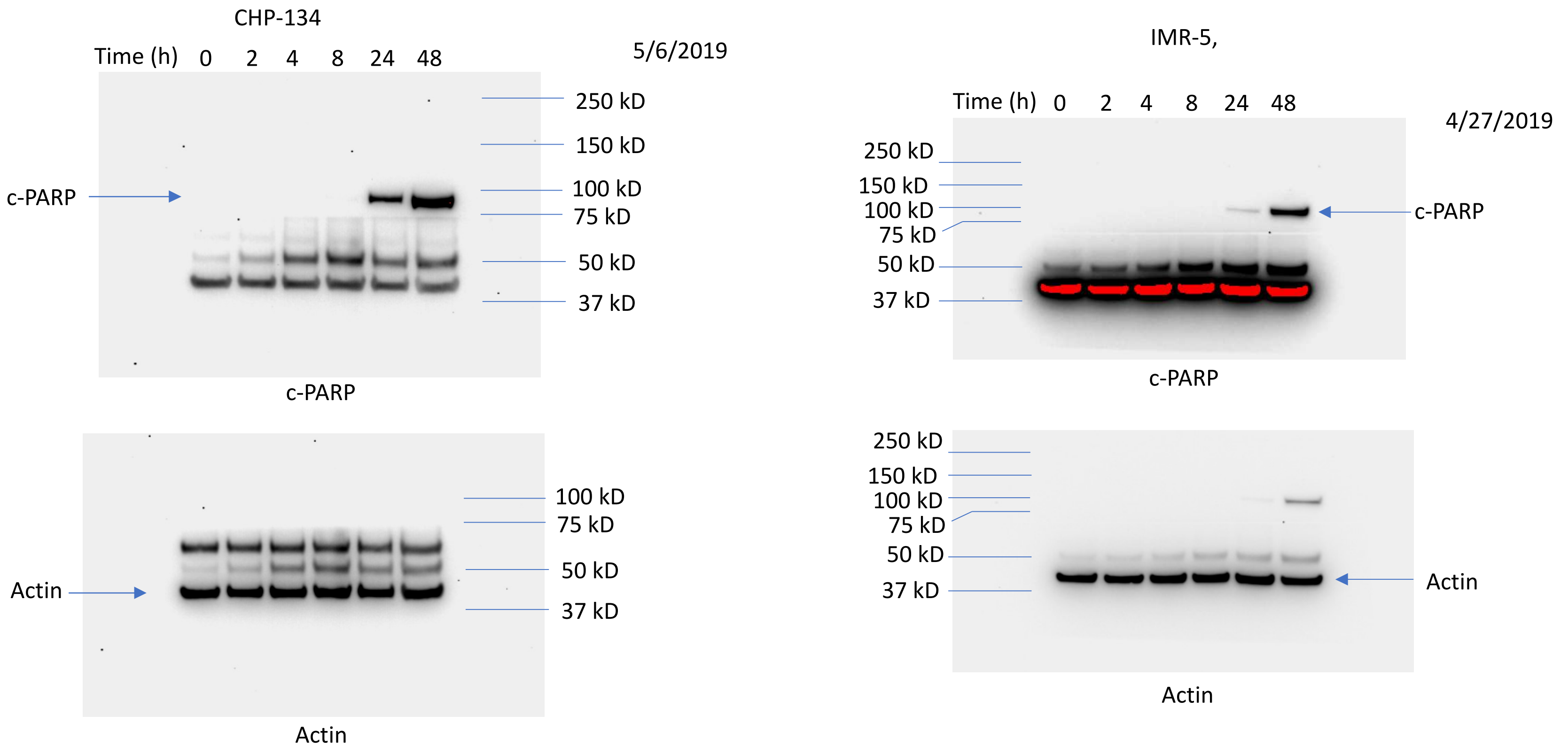

Figure 3d

Exp 2

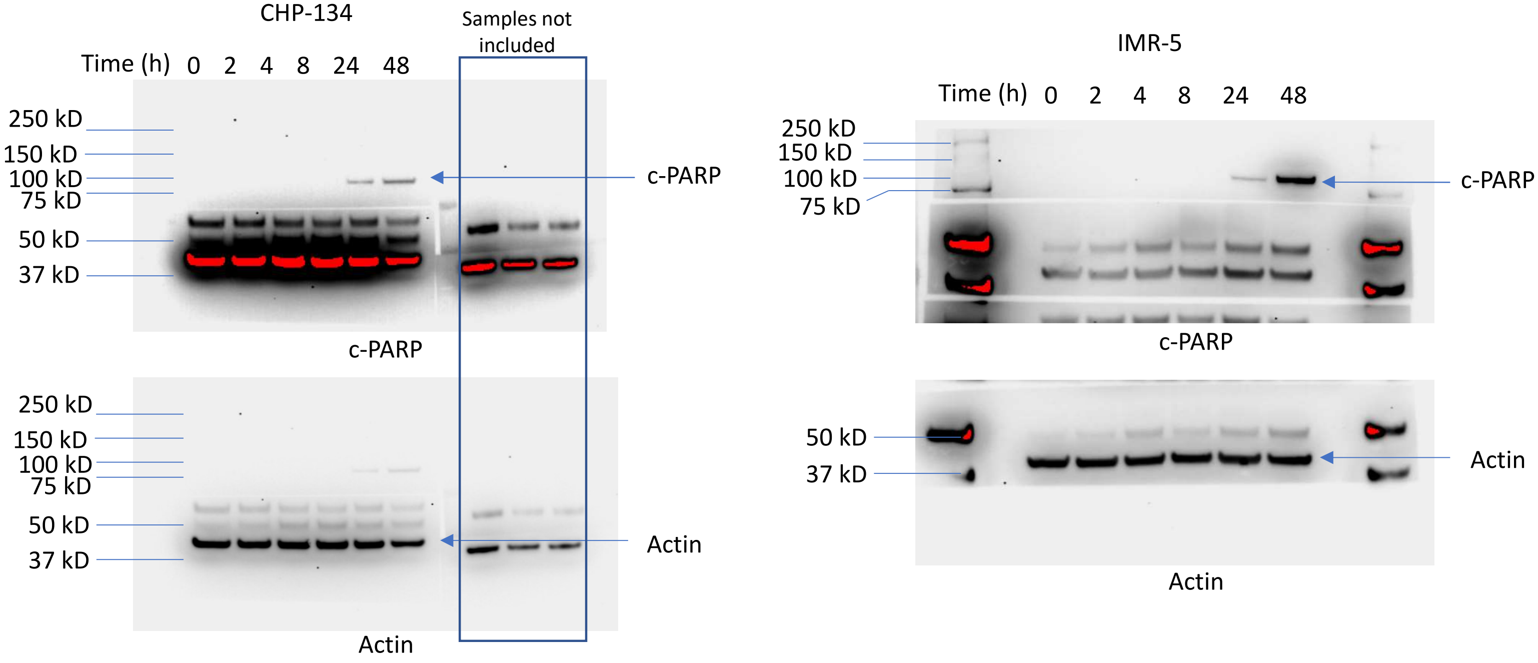

Figure 3d

Exp 3

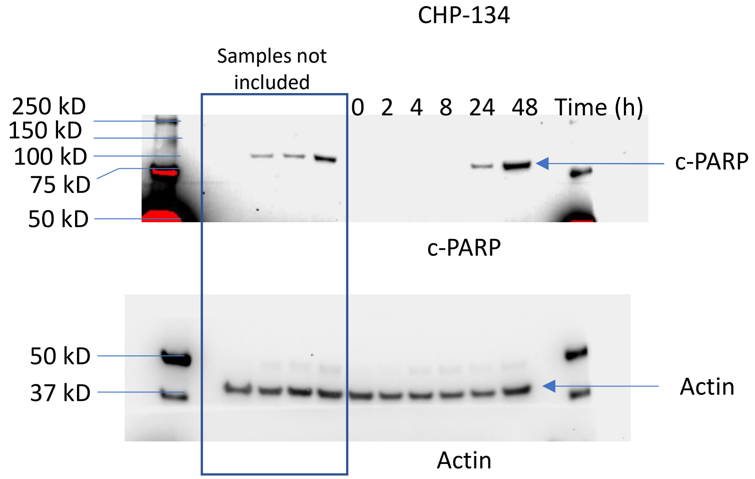

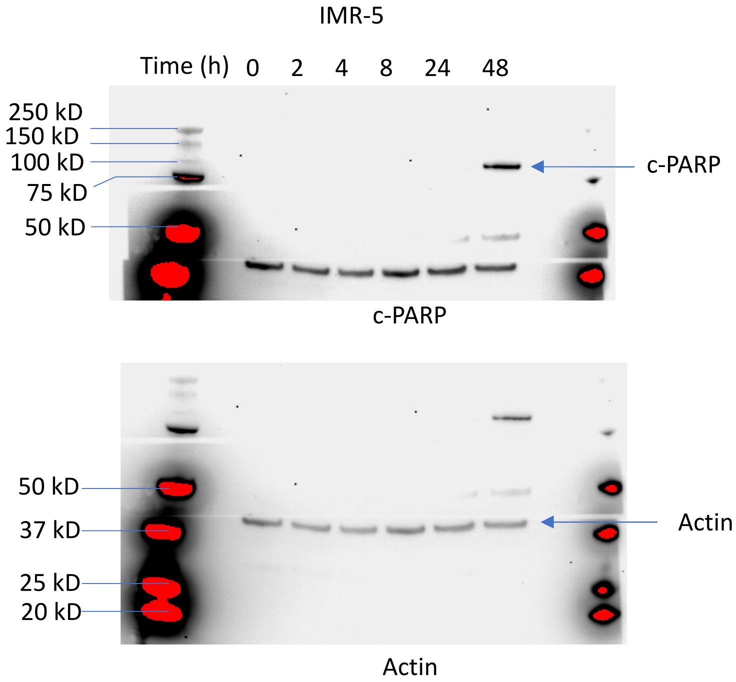

Figure 4e

Exp 1

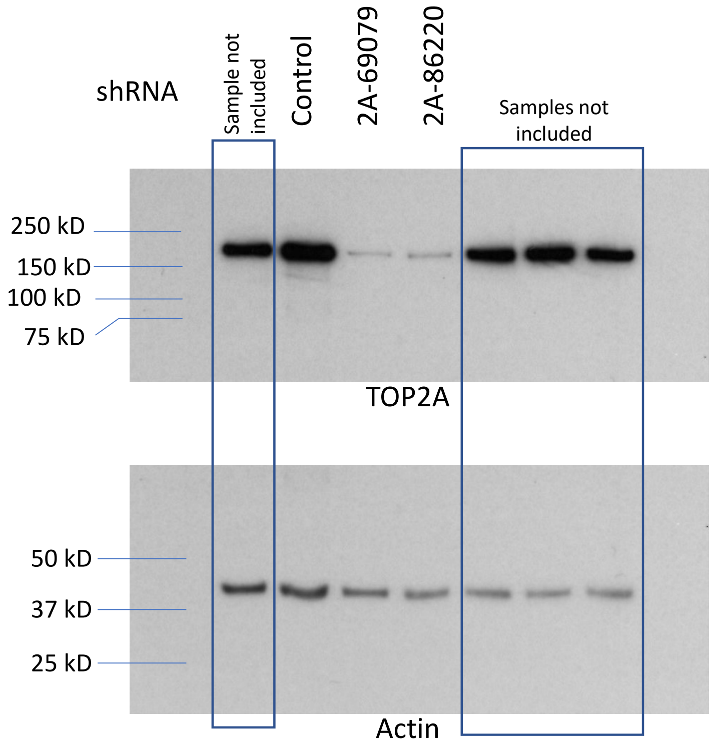

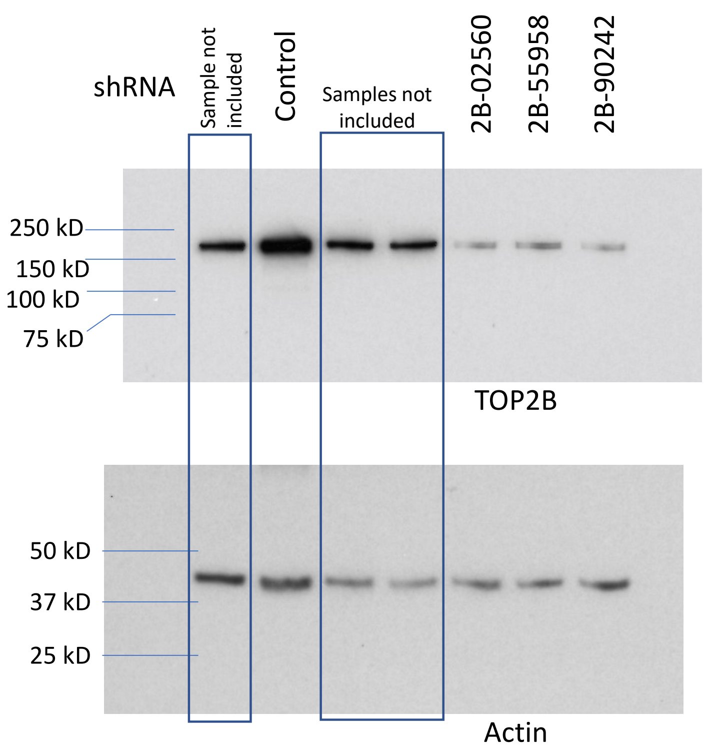

Figure 4e

Exp 2

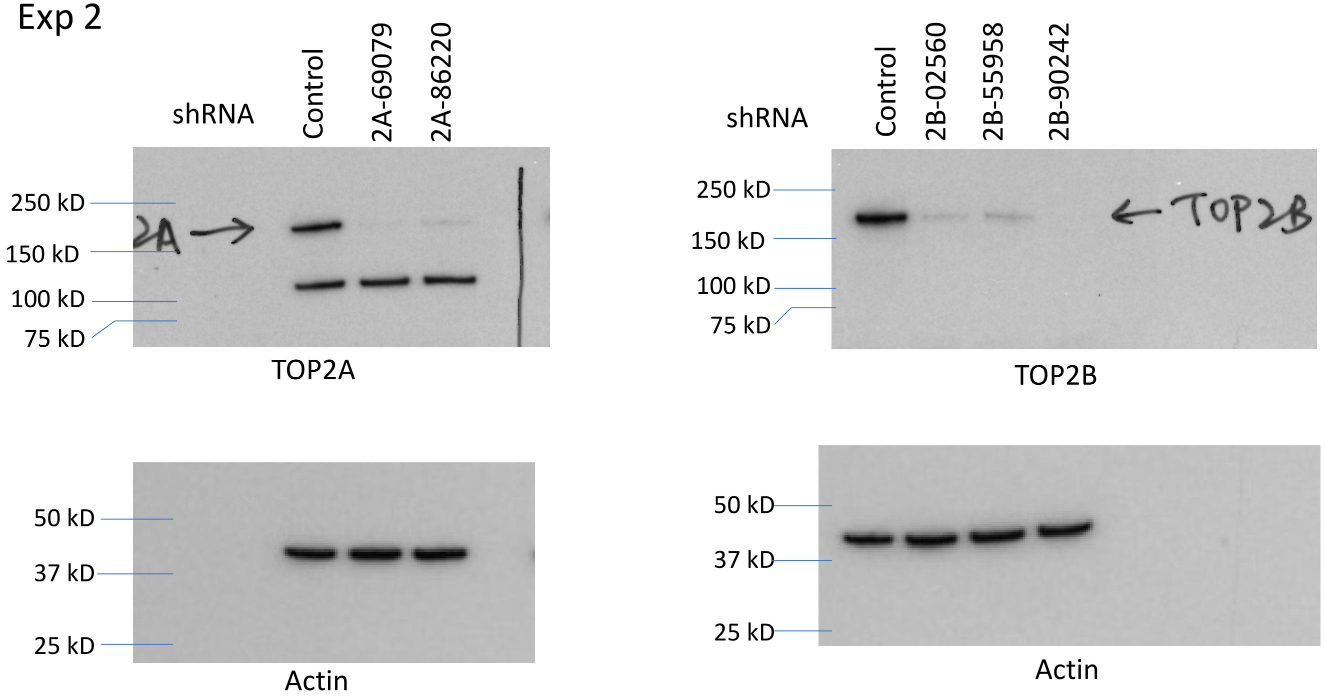

Figure 4e

Exp 3

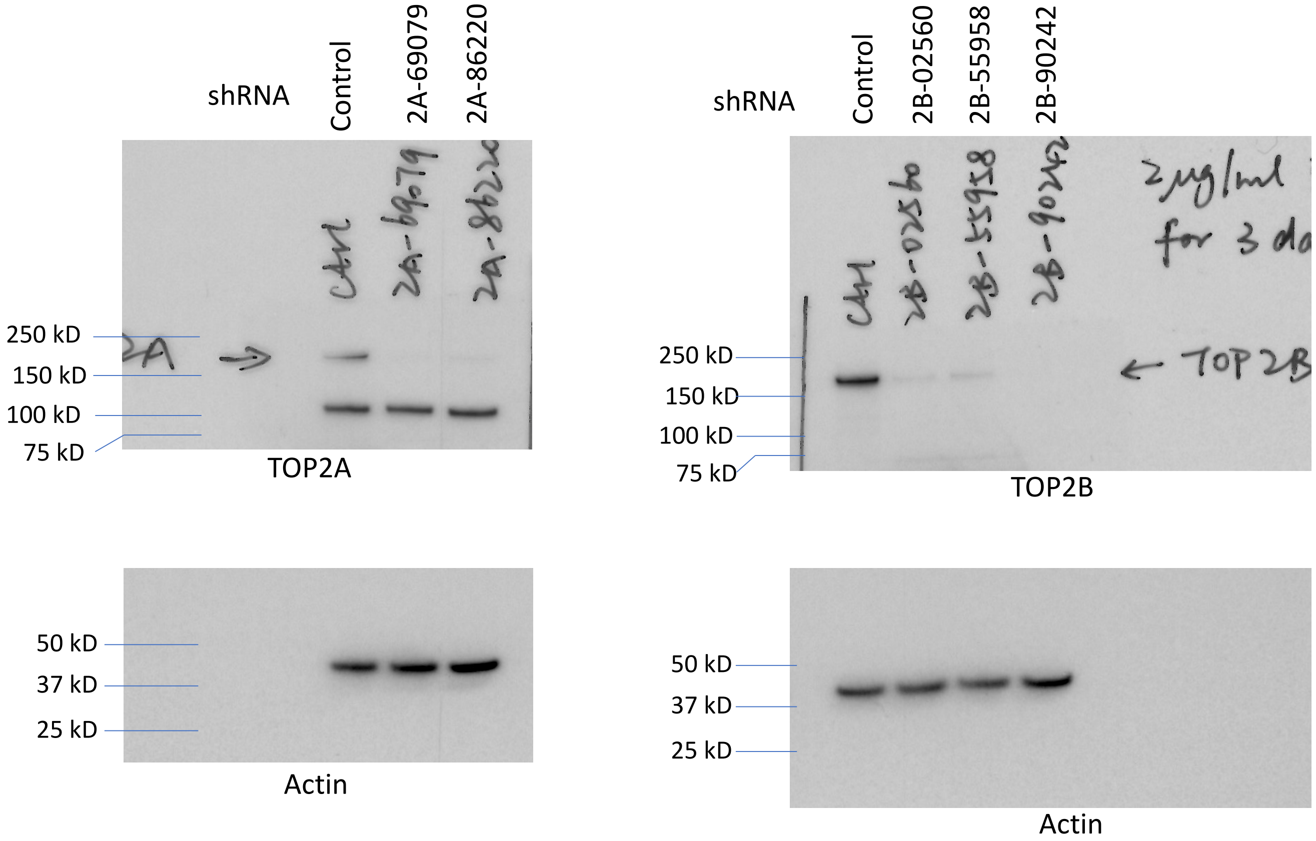

Figure 4h

Exp 1

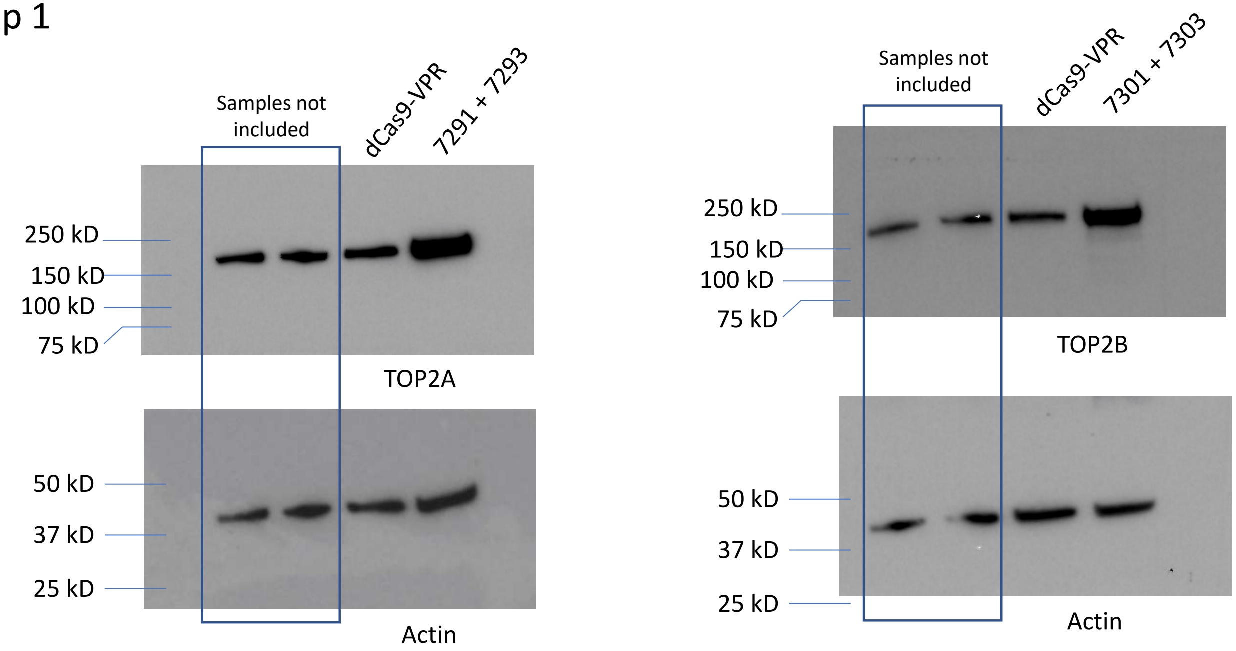

Figure 4h

Exp 2

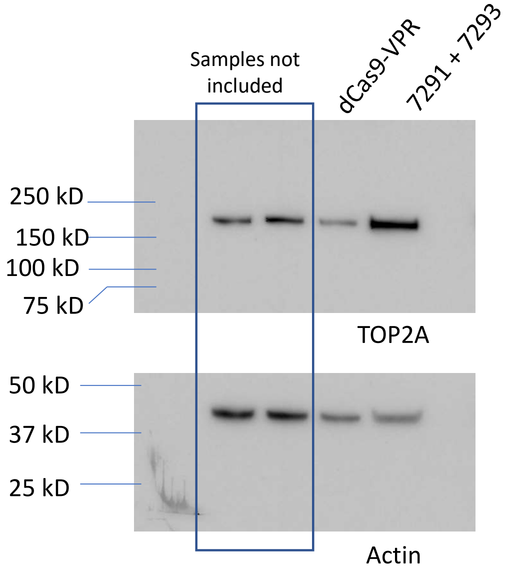

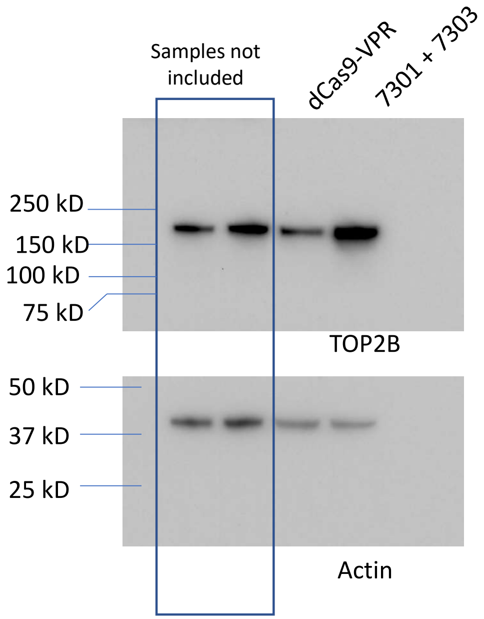

Figure 4h

Exp 3

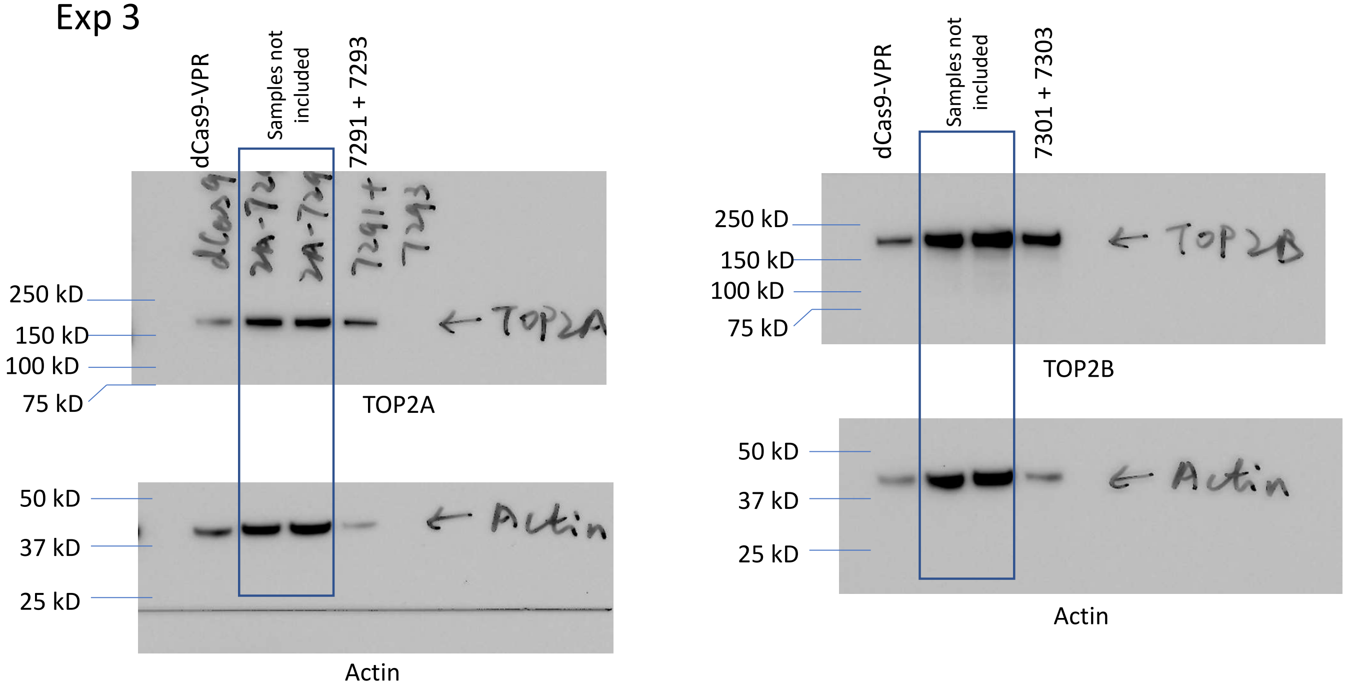

Figure S3g

Exp 1

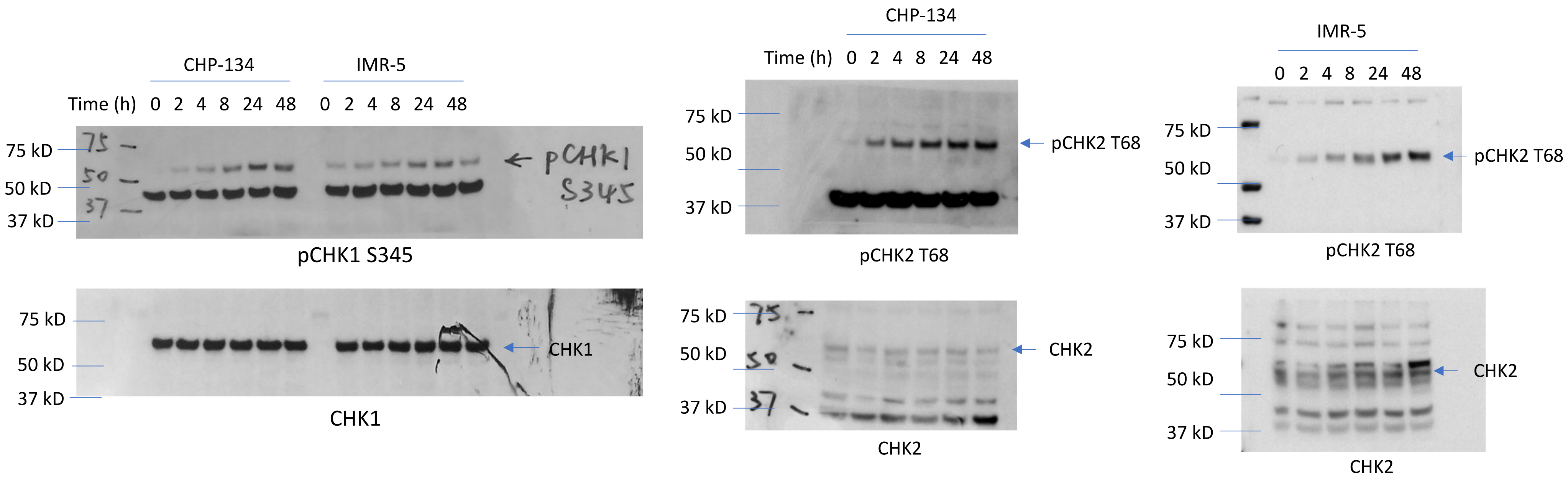

Figure S3g

Exp 2

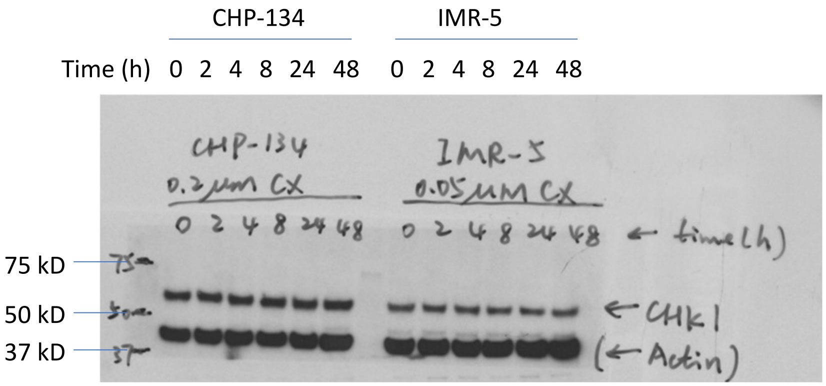

CHK1

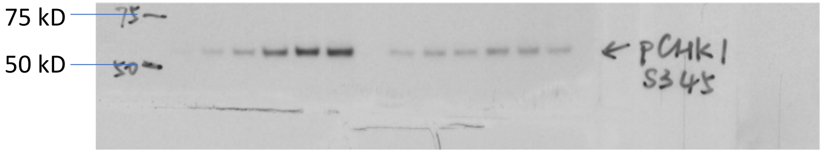

pCHK1 S345

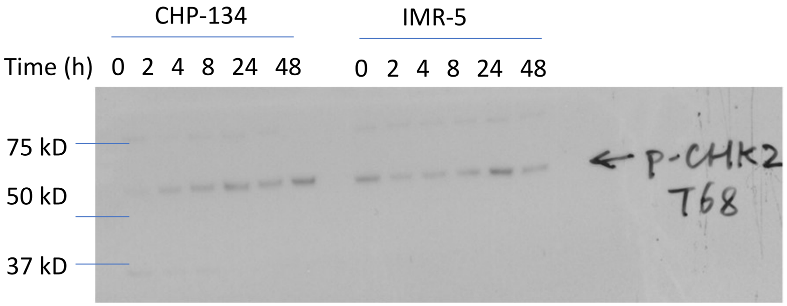

pCHK2 T68

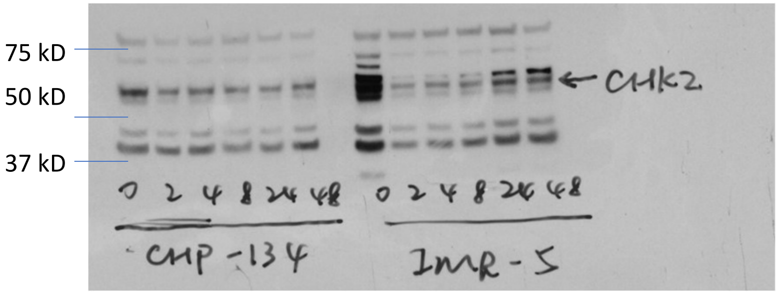

CHK2

Figure S3h

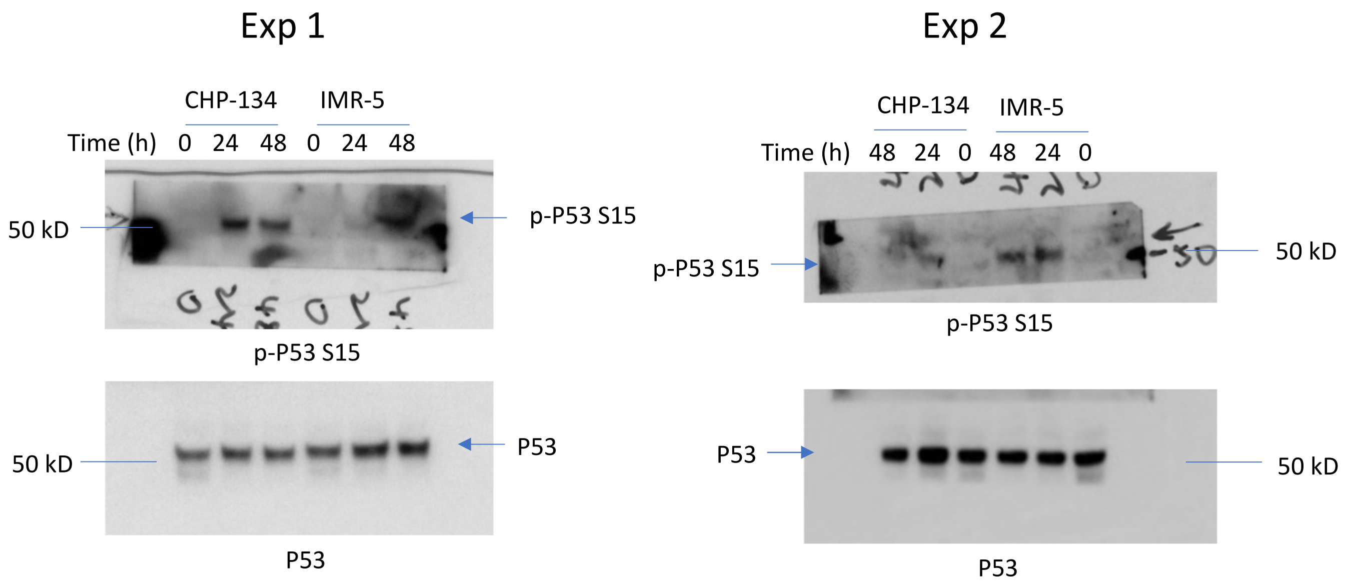
Exp 1
CHP-134
IMR-5
Time (h) 0 24 48 0 24 48
50 kD
p-P53 S15
p-P53 S15
50 kD
P53
P53
Exp 2
CHP-134
IMR-5
Time (h) 48 24 0 48 24 0
p-P53 S15
50 kD
p-P53 S15
P53
50 kD
P53

Figure S4c

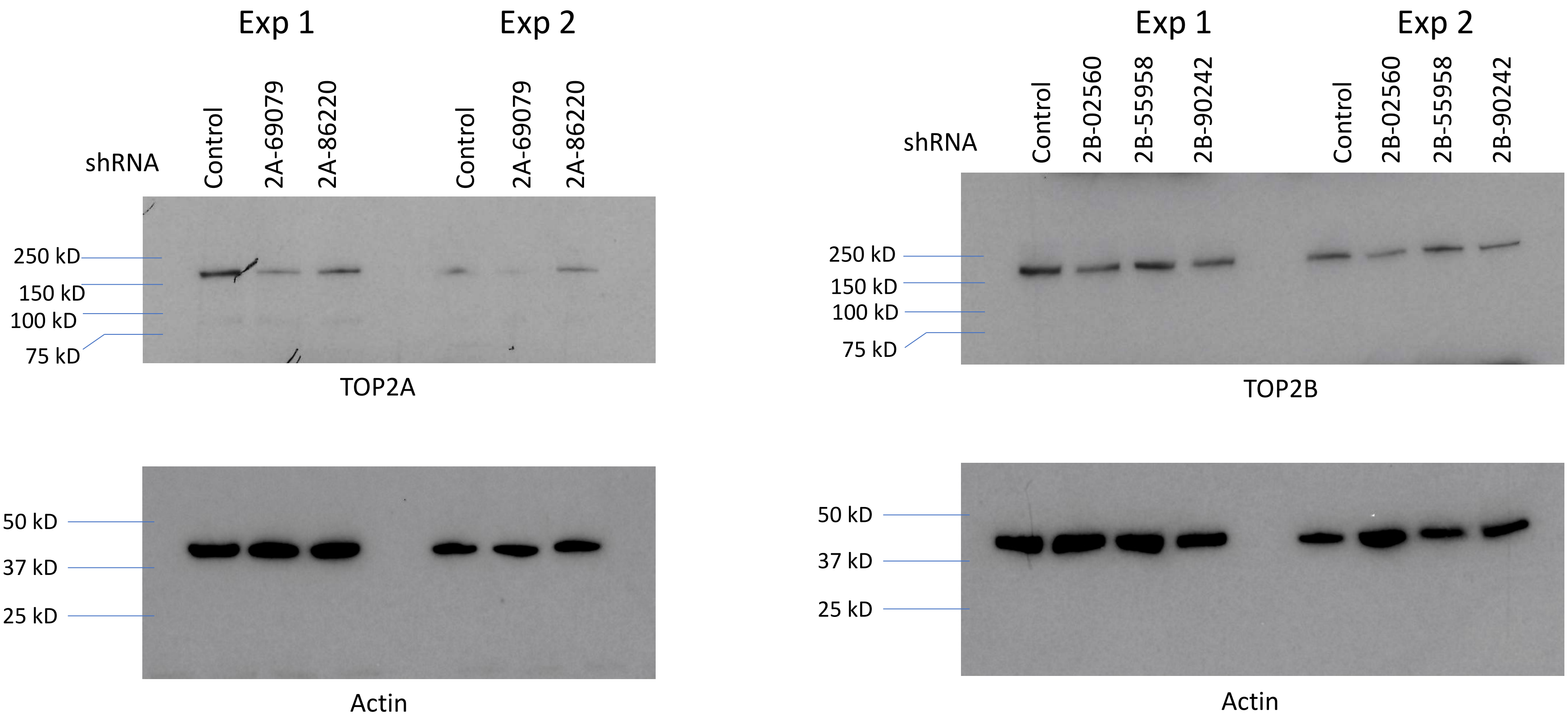

Exp 1
Exp 2
shRNA
Control
2A-69079
2A-86220
Control
2A-69079
2A-86220
250 kD
150 kD
100 kD
75 kD
TOP2A
50 kD
37 kD
25 kD
Actin
Exp 1
Exp 2
shRNA
Control
2B-02560
2B-55958
2B-90242
Control
2B-02560
2B-55958
2B-90242
250 kD
150 kD
100 kD
75 kD
TOP2B
50 kD
37 kD
25 kD
Actin

Figure S4c

Exp 3

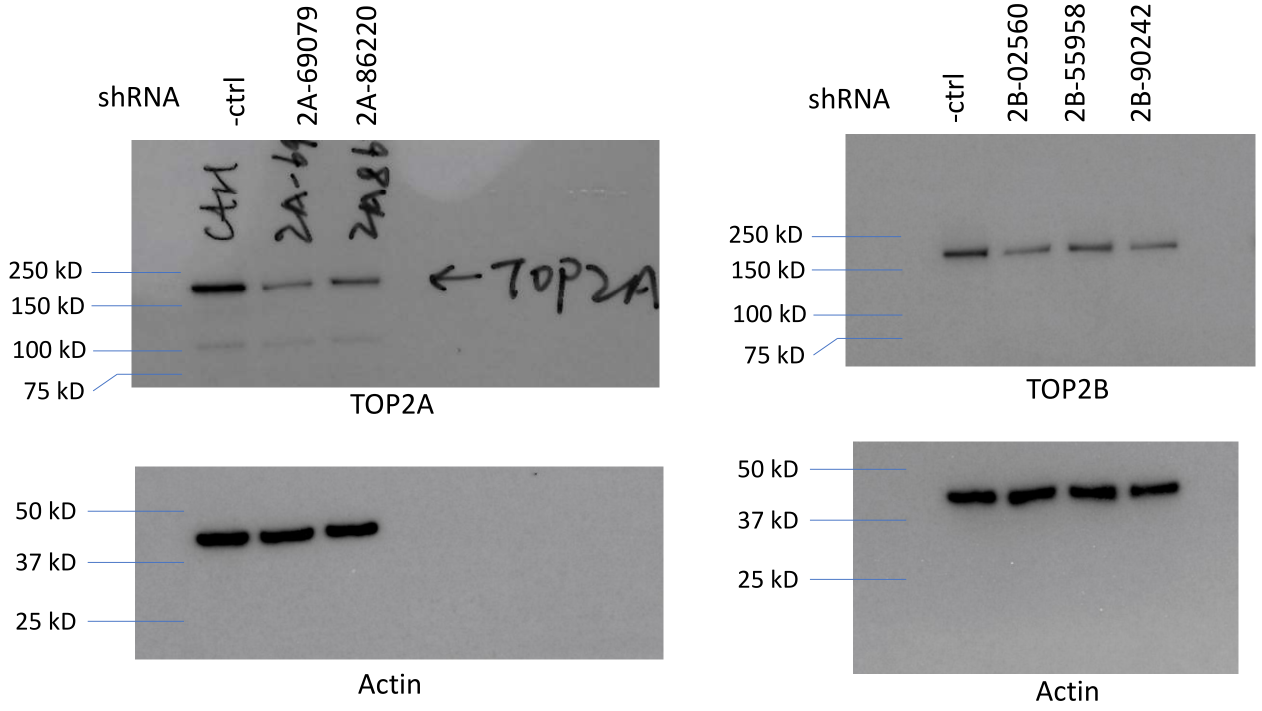

Figure S6n

Exp 1

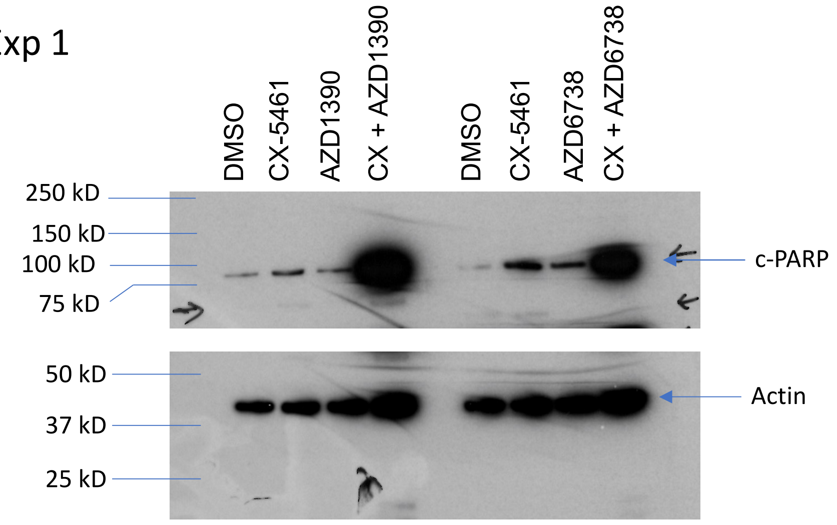

Exp 2

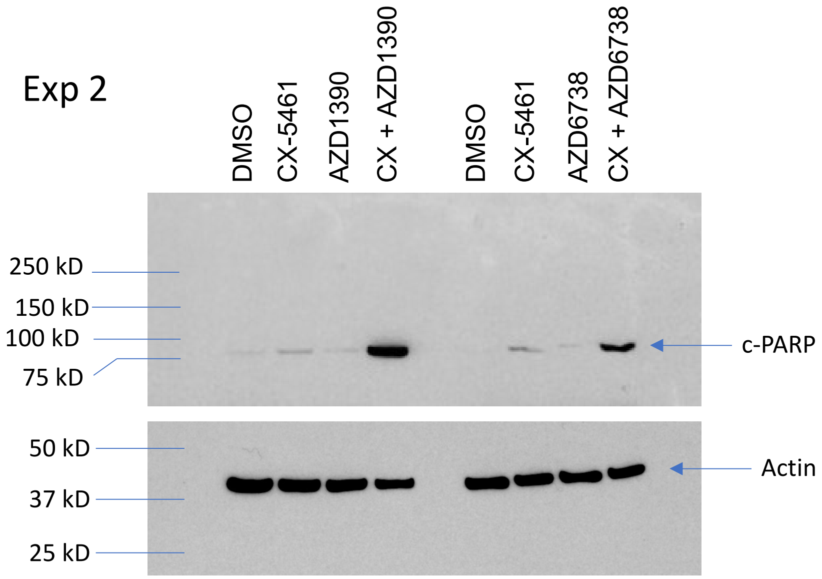

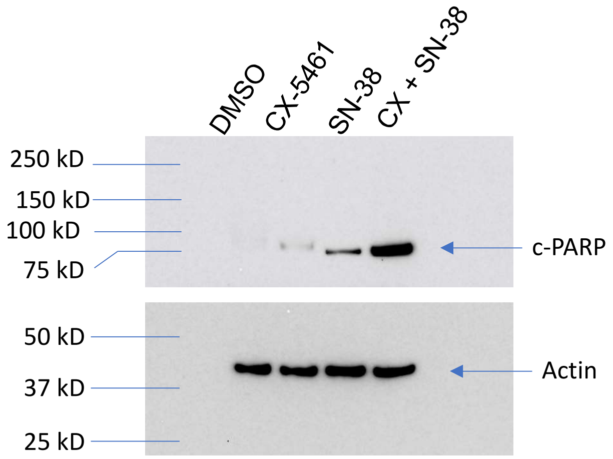

Supplementary Methods

Preclinical pharmacokinetic reports for Supplementary Figure 7a-c.

Preclinical pharmacokinetic report for Supplementary Figure 7a.

Quality Statement

This non-GLP study was conducted using sound scientific principles and established techniques in accordance with the relevant guidelines and standard operating procedures (SOPs) of the Preclinical Pharmacokinetic Shared Resource (P-PKSR) and St. Jude Children's Research Hospital (SJCRH), Memphis, TN, USA. This report accurately reflects the data obtained during the course of this study.

These results represent part of an early phase preclinical pharmacology program. This study has been conducted to provide preliminary insights into the pharmacokinetic (PK) properties of the compound(s) in the indicated preclinical model(s). This study and its results are not intended to provide a comprehensive PK evaluation of the compound(s). The applied bioanalytical method was validated/qualified to support this specific study and discovery-style sample analyses.

Substantial study-to-study and inter-animal variability in preclinical PK exists. Such variability depends upon the in vivo scientists' experience, variations in compound purity and formulation, animal strains, sex and age, and other situational fixed effects (i.e. husbandry conditions, chow constituents, presence or absence of disease, concomitant drugs). As such, the actual PK, plasma or tissue compound concentrations, or equivalent dose in other studies or preclinical models may vary significantly from that reported herein.

1.0 METHODS

1.1 In Vivo Pharmacokinetic (PK) Study

The plasma pharmacokinetic (PK) profile of Test Article CX-5461 as the dihydrochloride salt was evaluate in normal female Athymic nude mice (Charles River), approximately 12 weeks in age. CX-5461 2HCl (SJ000879909-4, MedChemExpress, HY-13323A, 31261) was dissolved in 10% Captisol (SBECD, Ligand Pharmaceuticals) in 25 mM NaH2PO4 (pH 5.95), for a 5 mg/kg free base equivalents dose as a 5 mL/kg intravenous tail vein injection. Two survival blood samples were obtained from each mouse via retro-orbital plexus using 50 µL Minivette POCT K3EDTA capillary devices (Sarstedt), and a third final sample by cardiac puncture. Samples were obtained at various times up to 24 hours post-dose, immediately processed to plasma, and stored at -80 °C until analysis. Remaining dosing solution was submitted for verification of potency, and chemical and physical stability during the study period.

1.2 Bioanalysis

Plasma samples were analyzed for CX-5461(SJ000879909-4, MedChemExpress, HY-13323A, 31261) with a qualified LC MS/MS assay. Plasma calibrators and quality controls were spiked with solutions, corrected for salt content, prepared in methanol. Plasma samples, 10 µL each, were protein precipitated with 100 µL of 0.1% formic acid in acetonitrile and 25 µL of 250 ng/mL alisertib (MedChemExpress, HY-10971, purity 99.43%) in methanol as an internal standard. A 2 µL aliquot of the extracted supernatant was injected onto a Shimadzu LC-20ADXR high performance liquid chromatography system via a Shimadzu SIL-20AC XR autosampler. The LC separation was performed using a Phenomenex Kinetex C18 (2.6 µm, 50 mm x 2.1 mm) column maintained at 40 °C with gradient elution at a flow rate of 0.25 mL/min. The binary mobile phase consisted of 0.1% formic acid in water-acetonitrile (90:10 v/v) in reservoir A and 0.1% formic acid in water-acetonitrile (1:99 v/v) in reservoir B. The initial mobile phase consisted of 10% B for 0.5 min with a linear increase to 80% B in 2 min. The column was then rinsed for 1 min at 80% B and then equilibrated at the initial conditions for 1.5 min for a total run time of 5 min. Under these conditions, the analyte and IS eluted at 2.71 and 3.68 min, respectively.

Analyte and IS were detected with tandem mass spectrometry using a SCIEX API 4000 in the positive ESI mode and the following mass transitions were monitored: CX-5461 514.2 -> 391.2, alisertib 519.2 -> 311.2. The method qualification and bioanalytical runs all passed acceptance criteria for non-GLP assay performance. A quadratic model ($1/X^2$ weighting) fit the calibrators across the 5 to 250 ng/mL range, with a correlation coefficient (R) of ≥ 0.9961. The lower limit of quantitation (LLOQ), defined as a peak area signal-to-noise ratio of 5 or greater verses a matrix blank with IS, was 5 ng/mL. Sample dilution integrity was confirmed. The intra-run precision and accuracy was ≤ 7.15% CV and 93.5% to 115%, respectively.

1.3 Pharmacokinetic (PK) Analysis

CX-5461 plasma Ct data were grouped by nominal time point, and the mean Ct values were subjected to noncompartmental analysis (NCA) using Phoenix WinNonlin 8.1 (Certara USA, Inc., Princeton, NJ). The IV Bolus model was applied, and area under the Ct curve (AUC) was estimated using the "linear up log down" method. The terminal phase was defined as at least three time points at the end of the Ct profile, and the elimination rate constant (Kel) was estimated using an unweighted log-linear regression of the terminal phase. The terminal elimination half-life (T1/2) was estimated as 0.693/Kel, and the AUC from time 0 to infinity (AUCinf) was estimated as the AUC to the last time point (AUClast) + Clast (predicted)/Kel. Other parameters estimated included observed maximum concentration (Cmax), time of Cmax (Tmax), concentration at the last observed time point (Clast), time of Clast (Tlast), back extrapolated initial concentration (C0), clearance (CL = Dose/AUCinf), volume of distribution at steady state (Vss), and terminal volume of distribution (Vz).

2.0 RESULTS

In the first 2 hours following IV tail vein injection, the CX-5461 plasma concentrations were highly variable, with the later time points showing low variability. Seven of the 9 mice studied showed rising post-dose concentrations upon repeated sampling: a disposition generally inconsistent with adequate IV dosing or sample collection. The mean Ct profile appeared nearly monophasic, with a suggestion of a subtle distribution and/or a possible saturable phase. The estimated terminal half-life using the last three observed time points was 3.7 hr. The total plasma clearance (CL) was low at 0.107 L/hr/kg or 1.78 mL/min/kg. The volume of distribution at steady state (Vss) was low-to-moderate at 0.626 L/kg, which approximates total body water. The total plasma AUCinf was 46900 hr*ug/L, which is 2.8-fold higher than the human AUClast at the maximum tolerated dose off 170 mg/m^2 IV [1]. Humans also exhibited a low CL of CX-5461 (0.25 L/hr/kg), but a much larger Vss (~17 L/kg), yielding a multi-phasic profile and a prolonged terminal half-life of ~50 hours. Compared with previously published mouse PK at an unknown IV dosage [2], our mice had higher CL and Vss values, and a shorter half-life by 2- to 3-fold. The formulation met specification (1.08 ± 0.0323 mg/mL) and was stable over the usage period of 4 days.

The plasma protein binding (pPB) of CX-5461 has not been reported in mice or humans, and as such a full clinically relevant dose (CRD) based upon unbound AUC or Cavg [3] cannot be proposed at this time. Given the vast discrepancies in Vss and terminal half-life between mice and humans, a further PK study of an extravascular dose (oral or IP) should be considered.

3.0 REFERENCES

1. Khot A, Brajanovski N, Cameron DP, Poortinga G, Sanij E, Lim J, Soong J, Hein N, Link E, Pearson RB, Hannan R, McArthur GA, Harrison SJ. RNA Polymerase 1 Transcription Inhibitor CX-5461 in Patients with Advanced Hematologic Malignancies: Results of a Phase I First in Human Study. Blood. 2017 Dec 7;130(Suppl 1):3835–3835.

2. Haddach M, Schwaebe MK, Michaux J, Nagasawa J, O'Brien SE, Whitten JP, Pierre F, Kerdoncuff P, Darjania L, Stansfield R, Drygin D, Anderes K, Proffitt C, Bliesath J, Siddiqui-Jain A, Omori M, Huser N, Rice WG, Ryckman DM. Discovery of CX-5461, the First Direct and Selective Inhibitor of RNA Polymerase I, for Cancer Therapeutics. ACS Med Chem Lett. 2012 Jul 12;3(7):602–6.

3. Spilker ME, Chen X, Visswanathan R, Vage C, Yamazaki S, Li G, Lucas J, Bradshaw-Pierce EL, Vicini P. Found in Translation: Maximizing the Clinical Relevance of Nonclinical Oncology Studies. Clin Cancer Res. 2017 Feb 15;23(4):1080–90.

4.0 TABLES, LISTINGS, AND FIGURES (TLFS)

Figure 4.1: Mean (SD) Ct Profile of CX-5461 by Group

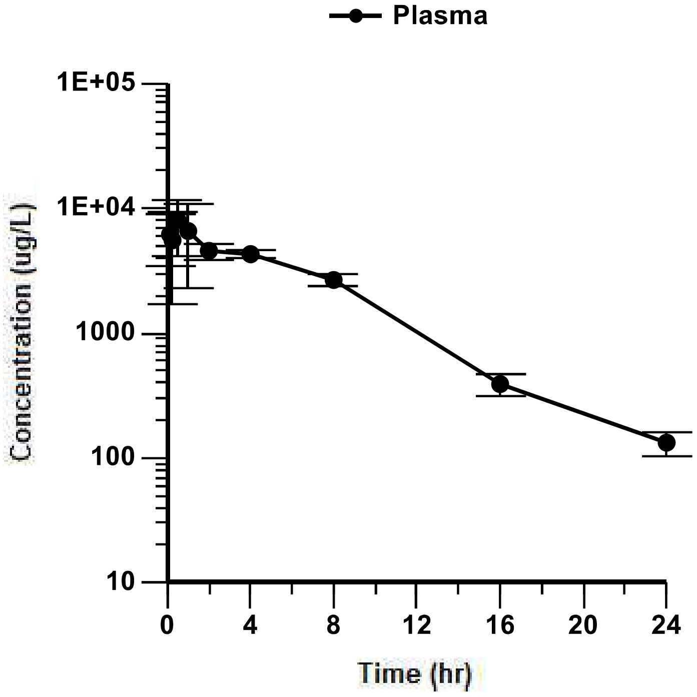

Table 4.1: NCA PK Parameter Estimates of CX-5461 by Group

		Analyte
		CX-5461
		Group
		Plasma
**Parameter**	**Units**	**Estimate**
Cmax	ug/L	7980
Tmax	hr	0.500
AUClast	hr*ug/L	46300
AUCinf	hr*ug/L	46900
Kel	1/hr	0.188
T1/2	hr	3.70

		Analyte
		CX-5461
		Group
		Plasma
**Parameter**	**Units**	**Estimate**
Clast	ug/L	133
Tlast	hr	24.0
C0	ug/L	6890
CL	L/hr/kg	0.107
Vss	L/kg	0.626
Vz	L/kg	0.569

Table 4.2: Summary Statistics of CX-5461 Ct Data by Group

		Analyte
		CX-5461
		Group
		Plasma
**Time (hr)**		**Concentration (ug/L)**
0.125	N	3
	Mean	6180
	SD	2740
	Min	3210
	Median	6710
	Max	8620
	CV%	44.4
	Geometric Mean	5710
	CV% Geometric Mean	54.9
0.250	N	3
	Mean	5540
	SD	3810
	Min	1170
	Median	7400
	Max	8070
	CV%	68.6

		Analyte
		CX-5461
		Group
		Plasma
**Time (hr)**		**Concentration (ug/L)**
	Geometric Mean	4110
	CV% Geometric Mean	152
0.500	N	3
	Mean	7980
	SD	3790
	Min	3850
	Median	8800
	Max	11300
	CV%	47.4
	Geometric Mean	7260
	CV% Geometric Mean	61.0
1.000	N	3
	Mean	6570
	SD	4310
	Min	2070
	Median	7010
	Max	10600
	CV%	65.5
	Geometric Mean	5360
	CV% Geometric Mean	103
2.000	N	3
	Mean	4570
	SD	656
	Min	4160
	Median	4210
	Max	5320
	CV%	14.4
	Geometric Mean	4540
	CV% Geometric Mean	13.9
4.000	N	3

		Analyte
		CX-5461
		Group
		Plasma
Time (hr)		Concentration (ug/L)
	Mean	4290
	SD	341
	Min	3910
	Median	4390
	Max	4570
	CV%	7.96
	Geometric Mean	4280
	CV% Geometric Mean	8.12
8.000	N	3
	Mean	2670
	SD	301
	Min	2330
	Median	2770
	Max	2900
	CV%	11.3
	Geometric Mean	2650
	CV% Geometric Mean	11.7
16.000	N	3
	Mean	390
	SD	77.1
	Min	344
	Median	348
	Max	479
	CV%	19.7
	Geometric Mean	386
	CV% Geometric Mean	19.0
24.000	N	3
	Mean	133
	SD	30.7
	Min	97.2

		Analyte
		CX-5461
		Group
		Plasma
Time (hr)		Concentration (ug/L)
	Median	149
	Max	152
	CV%	23.2
	Geometric Mean	130
	CV% Geometric Mean	25.6

Table 4.3: CX-5461 Ct Data Listings by Subject, Analyte, Group, and Time

Subject	Analyte	Group	Time (hr)	Concentration (ug/L)
M1	CX-5461	Plasma	0.13	3211.50
M1	CX-5461	Plasma	0.50	3852.50
M1	CX-5461	Plasma	16.00	348.29
M2	CX-5461	Plasma	0.13	8623.90
M2	CX-5461	Plasma	0.50	8796.30
M2	CX-5461	Plasma	16.00	479.41
M3	CX-5461	Plasma	0.13	6706.00
M3	CX-5461	Plasma	0.50	11292.00
M3	CX-5461	Plasma	16.00	343.57
M4	CX-5461	Plasma	0.25	1166.30
M4	CX-5461	Plasma	1.00	2065.80
M4	CX-5461	Plasma	24.00	151.76
M5	CX-5461	Plasma	0.25	8066.90
M5	CX-5461	Plasma	1.00	7010.10
M5	CX-5461	Plasma	24.00	97.16
M6	CX-5461	Plasma	0.25	7395.70
M6	CX-5461	Plasma	1.00	10647.00
M6	CX-5461	Plasma	24.00	148.87
M7	CX-5461	Plasma	2.00	4213.50
M7	CX-5461	Plasma	4.00	4393.20
M7	CX-5461	Plasma	8.00	2766.00

Subject	Analyte	Group	Time (hr)	Concentration (ug/L)
M8	CX-5461	Plasma	2.00	5323.10
M8	CX-5461	Plasma	4.00	3908.50
M8	CX-5461	Plasma	8.00	2327.30
M9	CX-5461	Plasma	2.00	4161.50
M9	CX-5461	Plasma	4.00	4567.50
M9	CX-5461	Plasma	8.00	2904.60

Table 4.4: CX-5461 Ct Summary (Mean, SD, N) by Group

Variable	Units	Analyte	Group	Time (hr)	Mean (ug/L)	SD (ug/L)	N
Concentration	ug/L	CX-5461	Plasma	0.13	6180.47	2744.20	3.00
Concentration	ug/L	CX-5461	Plasma	0.25	5542.97	3805.13	3.00
Concentration	ug/L	CX-5461	Plasma	0.50	7980.27	3786.29	3.00
Concentration	ug/L	CX-5461	Plasma	1.00	6574.30	4307.17	3.00
Concentration	ug/L	CX-5461	Plasma	2.00	4566.03	656.15	3.00
Concentration	ug/L	CX-5461	Plasma	4.00	4289.73	341.47	3.00
Concentration	ug/L	CX-5461	Plasma	8.00	2665.97	301.37	3.00
Concentration	ug/L	CX-5461	Plasma	16.00	390.42	77.10	3.00
Concentration	ug/L	CX-5461	Plasma	24.00	132.60	30.72	3.00

Preclinical pharmacokinetic report for Supplementary Figure 7b.

Quality Statement

This non-GLP study was conducted using sound scientific principles and established techniques in accordance with the relevant guidelines and standard operating procedures (SOPs) of the Preclinical Pharmacokinetic Shared Resource (P-PKSR) and St. Jude Children's Research Hospital (SJCRH), Memphis, TN, USA. This report accurately reflects the data obtained during the course of this study.

These results represent part of an early phase preclinical pharmacology program. This study has been conducted to provide preliminary insights into the pharmacokinetic (PK) properties of the compound(s) in the indicated preclinical model(s). This study and its results are not intended to provide a comprehensive PK evaluation of the compound(s). The applied bioanalytical method was validated/qualified to support this specific study and discovery-style sample analyses.

Substantial study-to-study and inter-animal variability in preclinical PK exists. Such variability depends upon the in vivo scientists' experience, variations in compound purity and formulation, animal strains, sex and age, and other situational fixed effects (i.e. husbandry conditions, chow constituents, presence or absence of disease, concomitant drugs). As such, the actual PK, plasma or tissue compound concentrations, or equivalent dose in other studies or preclinical models may vary significantly from that reported herein.

1.0 METHODS

1.1 In Vivo Pharmacokinetic (PK) Study

The plasma pharmacokinetic (PK) profile of CX-5461 as the dihydrochloride salt was evaluate in normal female Athymic nude mice (Charles River), approximately 12 weeks in age. CX-5461 2HCL (SJ000879909-4, MedChemExpress, HY-13323A, 31261) was dissolved in 10% Captisol (SBECD, Ligand Pharmaceuticals) in 25 mM NaH2PO4 (pH 5.95), for a 25 mg/kg free base equivalents dose as a 10 mL/kg intraperitoneal injection. Two survival blood samples were obtained from each mouse via retro-orbital plexus using 50 µL Minivette POCT K3EDTA capillary devices (Sarstedt), and a third final sample by cardiac puncture. Samples were obtained at various times up to 24 hours post-dose, immediately processed to plasma, and stored at -80 °C until analysis. Remaining dosing solution was submitted for verification of potency, and chemical and physical stability during the study period.

1.2 Bioanalysis

Plasma samples were analyzed for CX-5461(SJ000879909-4, MedChemExpress, HY-13323A, 31261) with a qualified LC MS/MS assay. Plasma calibrators and quality controls were spiked with solutions, corrected for salt content, prepared in methanol. Plasma samples, 10 µL each, were protein precipitated with 100 µL of 0.1% formic acid in acetonitrile and 25 µL of 250 ng/mL alisertib (MedChemExpress, HY-10971, purity 99.43%) in methanol as an internal standard. A 2 µL aliquot of the extracted supernatant was injected onto a Shimadzu LC-20ADXR high performance liquid chromatography system via a Shimadzu SIL-20AC XR autosampler. The LC separation was performed using a Phenomenex Kinetex C18 (2.6 µm, 50 mm x 2.1 mm) column maintained at 40 °C with gradient elution at a flow rate of 0.25 mL/min. The binary mobile phase consisted of 0.1% formic acid in water-acetonitrile (90:10 v/v) in reservoir A and 0.1% formic acid in acetonitrile in reservoir B. The initial mobile phase consisted of 10% B for 0.5 min with a linear increase to 80% B in 2 min. The column was then rinsed for 1 min at 80% B and then equilibrated at the initial conditions for 1.5 min for a total run time of 5 min. Under these conditions, the analyte and IS eluted at 2.71 and 3.68 min, respectively.

Analyte and IS were detected with tandem mass spectrometry using a SCIEX API 4000 in the positive ESI mode and the following mass transitions were monitored: CX-5461 514.2 -> 391.2, alisertib 519.2 -> 311.2. The method qualification and bioanalytical runs all passed acceptance criteria for non-GLP assay performance. A quadratic model ($1/X^2$ weighting) fit the calibrators across the 5 to 250 ng/mL range, with a correlation coefficient (R) of ≥ 0.9961. The lower limit of quantitation (LLOQ), defined as a peak area signal-to-noise ratio of 5 or greater verses a matrix blank with IS, was 5 ng/mL. Sample dilution integrity was confirmed. The intra-run precision and accuracy was ≤ 7.15% CV and 93.5% to 115%, respectively.

1.3 Pharmacokinetic (PK) Analysis

CX-5461 plasma Ct data were grouped by nominal time point, and the mean Ct values were subjected to noncompartmental analysis (NCA) using Phoenix WinNonlin 8.1 (Certara USA, Inc., Princeton, NJ). The extravascular model was applied, and area under the Ct curve (AUC) values were estimated using the "linear up log down" method. The terminal phase was defined as at least three time points at the end of the Ct profile, and the elimination rate constant (Kel) was estimated using an unweighted log-linear regression of the terminal phase. The terminal elimination half-life (T1/2) was estimated as 0.693/Kel, and the AUC from time 0 to infinity (AUCinf) was estimated as the AUC to the last time point (AUClast) + Clast (predicted)/Kel. Other parameters estimated included observed maximum concentration (Cmax), time of Cmax (Tmax), concentration at the last observed time point (Clast), time of Clast (Tlast), apparent clearance (CL/F = Dose/AUCinf), and apparent terminal volume of distribution (Vz/F).

2.0 RESULTS

The plasma PK of CX-5461 after IP injection showed extremely high variability, particularly between mice. Mouse group 3, which was sampled at 2, 4, and 8 hours post-dose, showed lower exposure and slow

appearance of compound in the plasma compared with the other groups of mice (**Attached File 5.6**). This may have been an artifact of the unbalanced batched sampling scheme. This phenomenon may also artificially inflate the overall terminal half-life estimate. Compared with the previous plasma PK study at 5 mg/kg IV, the exposures were approximately 3.3-fold higher. The Clast value of this current study is 21-fold higher than from the previous IV study. This may suggest solubility limited absorption and/or a saturable process producing nonlinear PK. The remaining formulation met specification (2.33 ± 0.0508 mg/mL) and was stable for 6 days.

In vitro plasma protein binding of CX-5461 was assessed using rapid equilibrium dialysis at 10 µM by CBT ATC, yielding fractions unbound in plasma (Fu,p) of 0.0449 and 0.0413 for mice and humans, respectively. These results suggests no significant difference between mouse and human plasma binding under the tested conditions.

At the recommended Phase II dose (RP2D) of 170 mg/m^2 IV as a 1 hour infusion every 3 weeks, adults with hematological malignancies exhibited a total plasma AUCinf of 17943 hr-ug/L [1], with a rapid distribution half-life of ~2 hr and terminal half-life of ~50 hr. The majority of the AUC (~90%) was represented by the terminal phase. The terminal half-life appeared to increase with dose, which the investigators attributed to suspected enterohepatic recirculation. The overall plasma Cavg, which included the infusion and distribution phases, over the first 168 hours post-dose was ~100 ug/L. Additional dose ranging of infusions on D1 and D8 of a 4-week schedule is ongoing in a separate Phase I study [2]; however, the RP2D has yet to be reported. This study has also described "nonproportional" increases in Cmax and AUC with dose level suggestive of nonlinear PK.

Given the variability between and within mice, and evidence of potential nonlinear PK in mice and humans, a precise PK-derived clinically relevant dose (CRD) for CX-5461 cannot be derived at the time of this report. A mouse low and multiple dose regimen maintaining the plasma Cavg in the 100 ug/L range for 5-14 days may constitute a reasonable CRD.

3.0 REFERENCES

1. Khot A, Brajanovski N, Cameron DP, Hein N, Maclachlan KH, Sanij E, Lim J, Soong J, Link E, Blombery P, Thompson ER, Fellowes A, Sheppard KE, McArthur GA, Pearson RB, Hannan RD, Poortinga G, Harrison SJ. First-in-Human RNA Polymerase I Transcription Inhibitor CX-5461 in Patients with Advanced Hematological Cancers: Results of a Phase I Dose Escalation Study. Cancer Discov. 2019 Jan 1;CD-18-1455.

2. Hilton J, Cescon DW, Bedard P, Ritter H, Tu D, Soong J, Gelmon K, Aparicio S, Seymour L. 44OCCTG IND.231: A phase 1 trial evaluating CX-5461 in patients with advanced solid tumors. Ann Oncol [Internet]. 2018 Mar 1 [cited 2019 May 30];29(suppl_3). Available from: https://academic.oup.com/annonc/article/29/suppl_3/mdy048.003/4917515

4.0 TABLES, LISTINGS, AND FIGURES (TLFS)

Figure 4.1: Mean (SD) Ct Profile of CX-5461 by Group

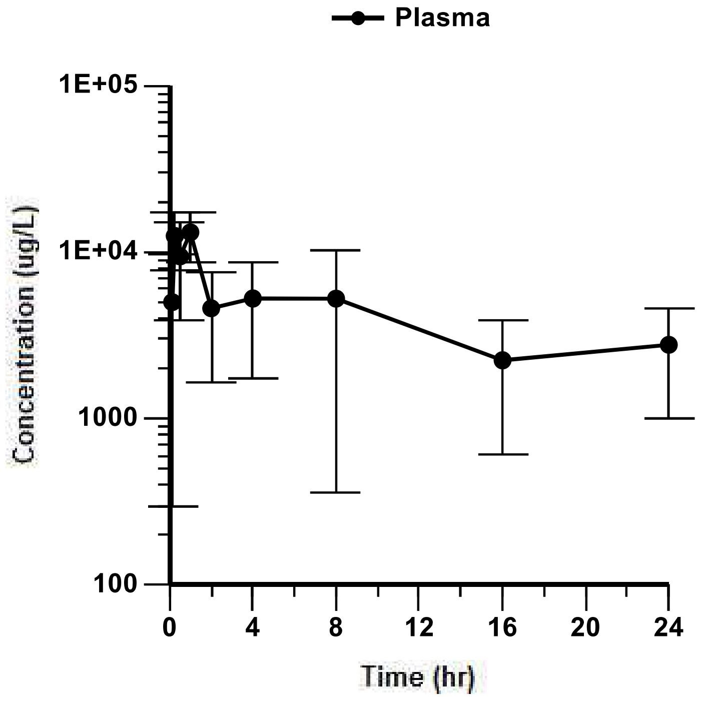

Table 4.1: NCA PK Parameter Estimates of CX-5461 by Group

		Analyte
		CX-5461
		Group
		Plasma
Parameter	Units	Estimate
Cmax	ug/L	13200
Tmax	hr	1.00
AUClast	hr*ug/L	97200
AUCinf	hr*ug/L	155000
Kel	1/hr	0.0399
T1/2	hr	17.4

		Analyte
		CX-5461
		Group
		Plasma
Parameter	Units	Estimate
CL/F	L/hr/kg	0.161
Vz/F	L/kg	4.03
Clast	ug/L	2780
Tlast	hr	24.0

Table 4.2: Full Summary Statistics of CX-5461 Ct Data by Group

		Analyte
		CX-5461
		Group
		Plasma
Time (hr)		Concentration (ug/L)
0.125	N	3
	Mean	5010
	SD	4720
	Min	1530
	Median	3120
	Max	10400
	CV%	94.1
	Geometric Mean	3680
	CV% Geometric Mean	124
0.250	N	3
	Mean	12500
	SD	4820
	Min	7110
	Median	14000
	Max	16400
	CV%	38.5
	Geometric Mean	11800
	CV% Geometric Mean	46.6

CX-5461 Screening Plasma PK (SPPK)

		Analyte
		CX-5461
		Group
		Plasma
Time (hr)		Concentration (ug/L)
0.500	N	3
	Mean	9430
	SD	5550
	Min	5810
	Median	6670
	Max	15800
	CV%	58.8
	Geometric Mean	8490
	CV% Geometric Mean	58.5
1.000	N	3
	Mean	13200
	SD	4400
	Min	9060
	Median	12600
	Max	17800
	CV%	33.4
	Geometric Mean	12700
	CV% Geometric Mean	34.8
2.000	N	3
	Mean	4600
	SD	2930
	Min	2330
	Median	3540
	Max	7910
	CV%	63.8
	Geometric Mean	4030
	CV% Geometric Mean	68.5
4.000	N	3
	Mean	5270
	SD	3530

		Analyte
		CX-5461
		Group
		Plasma
Time (hr)		Concentration (ug/L)
	Min	2780
	Median	3720
	Max	9310
	CV%	67.0
	Geometric Mean	4580
	CV% Geometric Mean	70.0
8.000	N	3
	Mean	5260
	SD	4900
	Min	1900
	Median	3000
	Max	10900
	CV%	93.1
	Geometric Mean	3960
	CV% Geometric Mean	113
16.000	N	3
	Mean	2240
	SD	1630
	Min	996
	Median	1640
	Max	4080
	CV%	72.6
	Geometric Mean	1880
	CV% Geometric Mean	81.7
24.000	N	3
	Mean	2780
	SD	1790
	Min	719
	Median	3670
	Max	3940

		Analyte
		CX-5461
		Group
		Plasma
Time (hr)		Concentration (ug/L)
	CV%	64.3
	Geometric Mean	2180
	CV% Geometric Mean	123

Table 4.3: CX-5461 Ct Data Listings by Subject, Analyte, Group, and Time

Subject	Analyte	Group	Time (hr)	Concentration (ug/L)
M1	CX-5461	Plasma	0.13	1532.90
M1	CX-5461	Plasma	0.50	15813.00
M1	CX-5461	Plasma	16.00	4080.90
M2	CX-5461	Plasma	0.13	3122.30
M2	CX-5461	Plasma	0.50	5807.90
M2	CX-5461	Plasma	16.00	996.31
M3	CX-5461	Plasma	0.13	10382.00
M3	CX-5461	Plasma	0.50	6665.90
M3	CX-5461	Plasma	16.00	1643.30
M4	CX-5461	Plasma	0.25	14026.00
M4	CX-5461	Plasma	1.00	12600.00
M4	CX-5461	Plasma	24.00	3667.40
M5	CX-5461	Plasma	0.25	16386.00
M5	CX-5461	Plasma	1.00	17803.00
M5	CX-5461	Plasma	24.00	3939.30
M6	CX-5461	Plasma	0.25	7114.50
M6	CX-5461	Plasma	1.00	9059.60
M6	CX-5461	Plasma	24.00	719.11
M7	CX-5461	Plasma	2.00	7910.40
M7	CX-5461	Plasma	4.00	9313.70
M7	CX-5461	Plasma	8.00	10879.00
M8	CX-5461	Plasma	2.00	2332.50
M8	CX-5461	Plasma	4.00	2776.40

Subject	Analyte	Group	Time (hr)	Concentration (ug/L)
M8	CX-5461	Plasma	8.00	1896.90
M9	CX-5461	Plasma	2.00	3544.60
M9	CX-5461	Plasma	4.00	3721.80
M9	CX-5461	Plasma	8.00	3000.70

Table 4.4: CX-5461 Ct Summary (Mean, SD, N) by Group

Variable	Units	Analyte	Group	Time (hr)	Mean (ug/L)	SD (ug/L)	N
Concentration	ug/L	CX-5461	Plasma	0.13	5012.40	4717.63	3.00
Concentration	ug/L	CX-5461	Plasma	0.25	12508.83	4818.35	3.00
Concentration	ug/L	CX-5461	Plasma	0.50	9428.93	5545.38	3.00
Concentration	ug/L	CX-5461	Plasma	1.00	13154.20	4397.97	3.00
Concentration	ug/L	CX-5461	Plasma	2.00	4595.83	2933.78	3.00
Concentration	ug/L	CX-5461	Plasma	4.00	5270.63	3533.16	3.00
Concentration	ug/L	CX-5461	Plasma	8.00	5258.87	4898.37	3.00
Concentration	ug/L	CX-5461	Plasma	16.00	2240.17	1626.61	3.00
Concentration	ug/L	CX-5461	Plasma	24.00	2775.27	1785.87	3.00

Preclinical pharmacokinetic report for Supplementary Figure 7c.

Quality Statement

This non-GLP study was conducted using sound scientific principles and established techniques in accordance with the relevant guidelines and standard operating procedures (SOPs) of the Preclinical Pharmacokinetic Shared Resource (P-PKSR) and St. Jude Children's Research Hospital (SJCRH), Memphis, TN, USA. This report accurately reflects the data obtained during the course of this study.

These results represent part of an early phase preclinical pharmacology program. This study has been conducted to provide preliminary insights into the pharmacokinetic (PK) properties of the compound(s) in the indicated preclinical model(s). This study and its results are not intended to provide a comprehensive PK evaluation of the compound(s). The applied bioanalytical method was validated/qualified to support this specific study and discovery-style sample analyses.

Substantial study-to-study and inter-animal variability in preclinical PK exists. Such variability depends upon the in vivo scientists' experience, variations in compound purity and formulation, animal strains, sex and age, and other situational fixed effects (i.e. husbandry conditions, chow constituents, presence or absence of disease, concomitant drugs). As such, the actual PK, plasma or tissue compound concentrations, or equivalent dose in other studies or preclinical models may vary significantly from that reported herein.

1.0 METHODS

1.1 In Vivo Pharmacokinetic (PK) Study

The plasma and tumor pharmacokinetic (PK) profile of CX-5461 as the dihydrochloride salt was evaluate in female Athymic nude mice (Charles River), approximately 12 weeks in age, bearing MAST3 neuroblastoma orthotopic xenografts. CX-5461 2HCl (SJ000879909-4, MedChemExpress, HY-13323A, 31261) was dissolved in 10% Captisol (SBECD, Ligand Pharmaceuticals) in 25 mM NaH2PO4 (pH 5.95), for a 25 mg/kg free base equivalents dose as a 10 mL/kg intraperitoneal injection. Terminal blood samples, under IP Avertin (tribromoethanol) anesthesia, were obtained at various times up to 24 hours post-dose, immediately processed to plasma, and stored at -80 °C until analysis. Following terminal bleeds, animals were perfused with PBS to flush blood from the vasculature. Tumors were then extracted, rinsed with PBS as necessary, and then placed in appropriately labeled microcentrifuge tubes in a cooler on dry ice. Tissue samples were then transferred to a -80°C freezer as soon as possible. Remaining dosing solution was submitted for verification of potency, and chemical and physical stability during the study period.

1.2 Bioanalysis

Tumor samples were weighed in 15 mL TEENPREP Lysing matrix D (MP Biomedicals, Santa Ana, CA), diluted with a 1:5 volume of ultrapure water, and homogenized using a FastPrep-24 system (MP Biomedicals, Santa Ana, CA) for seven cycles of 1 min vibration at 6.5 M/S speed, with 5 min in ice bath between each cycle to prevent over-heating. The homogenates were then stored at -80 °C until analysis. Plasma and tumor samples were analyzed for CX-5461(SJ000879909-4, MedChemExpress, HY-13323A, 31261) with a qualified LC MS/MS assay.

Plasma calibrators and quality controls were spiked with solutions, corrected for salt content, prepared in methanol. Plasma samples, 10 µL each, were protein precipitated with 100 µL of 0.1% formic acid in acetonitrile and 25 µL of 250 ng/mL alisertib (MedChemExpress, HY-10971, purity 99.43%) in methanol as an internal standard. A 2 µL aliquot of the extracted supernatant was injected onto a Shimadzu LC-20ADXR high performance liquid chromatography system via a Shimadzu SIL-20AC XR autosampler. The LC separation was performed using a Phenomenex Kinetex C18 (2.6 µm, 50 mm x 2.1 mm) column maintained at 40 °C with gradient elution at a flow rate of 0.25 mL/min. The binary mobile phase consisted of 0.1% formic acid in water-acetonitrile (90:10 v/v) in reservoir A and 0.1% formic acid in acetonitrile in reservoir B. The initial mobile phase consisted of 10% B for 0.5 min with a linear increase to 80% B in 2 min. The column was then rinsed for 1 min at 80% B and then equilibrated at the initial conditions for 1.5 min for a total run time of 5 min. Under these conditions, the analyte and IS eluted at 2.71 and 3.68 min, respectively.

Analyte and IS were detected with tandem mass spectrometry using a SCIEX API 4000 in the positive ESI mode and the following mass transitions were monitored: CX-5461 514.2 -> 391.2, alisertib 519.2 -> 311.2. The method qualification and bioanalytical runs all passed acceptance criteria for non-GLP assay performance. A quadratic model ($1/X^2$ weighting) fit the calibrators across the 5 to 250 ng/mL range, with a correlation coefficient (R) of ≥ 0.9961. The lower limit of quantitation (LLOQ), defined as a peak area signal-to-noise ratio of 5 or greater verses a matrix blank with IS, was 5 ng/mL. Sample dilution integrity was confirmed. The intra-run precision and accuracy was ≤ 7.15% CV and 93.5% to 115%, respectively.

1.3 Pharmacokinetic (PK) Analysis

CX-5461 plasma and tumor Ct data were grouped by matrix and nominal time point, and the mean Ct values were subjected to noncompartmental analysis (NCA) using Phoenix WinNonlin 8.1 (Certara USA, Inc., Princeton, NJ). The extravascular model was applied, and area under the Ct curve (AUC) values were estimated using the "linear up log down" method. The terminal phase was defined as at least three time points at the end of the Ct profile, and the elimination rate constant (Kel) was estimated using an

unweighted log-linear regression of the terminal phase. The terminal elimination half-life (T1/2) was estimated as 0.693/Kel, and the AUC from time 0 to infinity (AUCinf) was estimated as the AUC to the last time point (AUClast) + Clast (predicted)/Kel. Other parameters estimated included observed maximum concentration (Cmax), time of Cmax (Tmax), concentration at the last observed time point (Clast), time of Clast (Tlast), apparent clearance (CL/F = Dose/AUCinf), and apparent terminal volume of distribution (Vz/F). The apparent plasma-to-tumor partition coefficient (Kp,inf) was estimated as the ratio of the AUCinf in tissue to AUCinf plasma, whereas Kp,last was similarly estimated using AUClast values.

2.0 RESULTS

The plasma and MAST3 neuroblastoma PK of CX-5461 were highly variable, with no discernable terminal phase for plasma. As such, all reported terminal parameters should be interpreted with caution. The plasma PK was rather distinct from the previous plasma only PK study, in that current observations showed a very flat profile with high concentrations and no apparent elimination phase. The absorption from the IP space appeared to be slow and variable, and the possibility of a saturable effect on absorption or metabolism / elimination cannot be ruled out. The plasma concentrations generally rose from 16 to 24 hours, which may suggest enterohepatic recirculation. CX-5461 tumor concentrations were slightly higher than unity compared with plasma (Kp,last = 1.16), with earlier time points showing lower concentration ratios, suggesting a slow permeation to tumor. The remaining formulation was found to be in specification.

At the recommended Phase II dose (RP2D) of CX-5461 of 170 mg/m^2 IV as a 1 hour infusion every 3 weeks, adults with hematological malignancies exhibited a total plasma AUCinf of 17943 hr-ug/L [1], with a rapid distribution half-life of ~2 hr and terminal half-life of ~50 hr. The majority of the AUC (~90%) was represented by the terminal phase. The terminal half-life appeared to increase with dose, which the investigators attributed to suspected enterohepatic recirculation. The overall plasma Cavg, which included the infusion and distribution phases, over the first 168 hours post-dose was ~100 ug/L. Additional dose ranging of infusions on D1 and D8 of a 4-week schedule is ongoing in a separate Phase I study [2]; however, the RP2D has yet to be reported. The major toxicity to date has been photosensitivity. This study has also described "nonproportional" increases in Cmax and AUC with dose level suggestive of nonlinear PK. Previous data from CBT ATC has shown plasma protein binding of CX-5461 in mice and humans to be similar (RPT.159452-1677129).

Given the difficulties and variable PK CX-5461 has exhibited in our mouse studies, recommendation of a PK-based clinically relevant dose (CRD) is difficult. This is made more challenging since human PK of CX-5461 is so qualitatively different. CX-5461 has exhibited characteristics of nonlinear and non-dose proportional PK in our mouse studies as well as in human studies, which makes extrapolation of dose-exposure relationships unreliable.

With these caveats, and with the weak assumption of linear and proportional PK, a CX-5461 dosage of 2.5 mg/kg (range 1.25 – 5 mg/kg) IP QD x 5d would likely provide a plasma Cavg comparable to the standard RP2D dose. To recapitulate the D1,D8 schedule, a second QD x 5d course would be needed starting on D8. Of note, typical mouse CX-5461 doses in the literature have ranged from 12 – 50 mg/kg PO or IP, from once weekly to a continuous 5-on, 2-off schedule [3–7].

3.0 REFERENCES

1. Khot A, Brajanovski N, Cameron DP, Hein N, Maclachlan KH, Sanij E, Lim J, Soong J, Link E, Blombery P, Thompson ER, Fellowes A, Sheppard KE, McArthur GA, Pearson RB, Hannan RD, Poortinga G, Harrison SJ. First-in-Human RNA Polymerase I Transcription Inhibitor CX-5461 in Patients with Advanced Hematological Cancers: Results of a Phase I Dose Escalation Study. Cancer Discov. 2019 Jan 1;CD-18-1455.

2. Hilton J, Cescon DW, Bedard P, Ritter H, Tu D, Soong J, Gelmon K, Aparicio S, Seymour L. 44OCCTG IND.231: A phase 1 trial evaluating CX-5461 in patients with advanced solid tumors. Ann Oncol [Internet]. 2018 Mar 1 [cited 2019 May 30];29(suppl_3). Available from: https://academic.oup.com/annonc/article/29/suppl_3/mdy048.003/4917515

3. Drygin D, Lin A, Bliesath J, Ho CB, O'Brien SE, Proffitt C, Omori M, Haddach M, Schwaebe MK, Siddiqui-Jain A, Streiner N, Quin JE, Sanij E, Bywater MJ, Hannan RD, Ryckman D, Anderes K, Rice WG. Targeting RNA Polymerase I with an Oral Small Molecule CX-5461 Inhibits Ribosomal RNA Synthesis and Solid Tumor Growth. Cancer Res. 2011 Feb 15;71(4):1418–30.

4. Haddach M, Schwaebe MK, Michaux J, Nagasawa J, O'Brien SE, Whitten JP, Pierre F, Kerdoncuff P, Darjania L, Stansfield R, Drygin D, Anderes K, Proffitt C, Bliesath J, Siddiqui-Jain A, Omori M, Huser N, Rice WG, Ryckman DM. Discovery of CX-5461, the First Direct and Selective Inhibitor of RNA Polymerase I, for Cancer Therapeutics. ACS Med Chem Lett. 2012 Jul 12;3(7):602–6.

5. Li L, Li Y, Zhao J, Fan S, Wang L, Li X. CX-5461 induces autophagy and inhibits tumor growth via mammalian target of rapamycin-related signaling pathways in osteosarcoma. OncoTargets Ther. 2016 Sep 29;9:5985–97.

6. Maclachlan KH, Cuddihy A, Hein N, Cullinane C, Harrison SJ, Hannan R, Poortinga G. Novel Combination Therapies with the RNA Polymerase I Inhibitor CX-5461 Significantly Improve Efficacy in Multiple Myeloma. Blood. 2017 Dec 7;130(Suppl 1):1805–1805.

7. Xu H, Di Antonio M, McKinney S, Mathew V, Ho B, O'Neil NJ, Santos ND, Silvester J, Wei V, Garcia J, Kabeer F, Lai D, Soriano P, Banáth J, Chiu DS, Yap D, Le DD, Ye FB, Zhang A, Thu K, Soong J, Lin S, Tsai AHC, Osako T, Algara T, Saunders DN, Wong J, Xian J, Bally MB, Brenton JD, Brown GW, Shah SP, Cescon D, Mak TW, Caldas C, Stirling PC, Hieter P, Balasubramanian S, Aparicio S. CX-5461 is a DNA G-quadruplex stabilizer with selective lethality in BRCA1/2 deficient tumours. Nat Commun. 2017 Feb 17;8:14432.

4.0 TABLES, LISTINGS, AND FIGURES (TLFS)

Figure 4.1: Mean (SD) Ct Profile of CX-5461 by Group

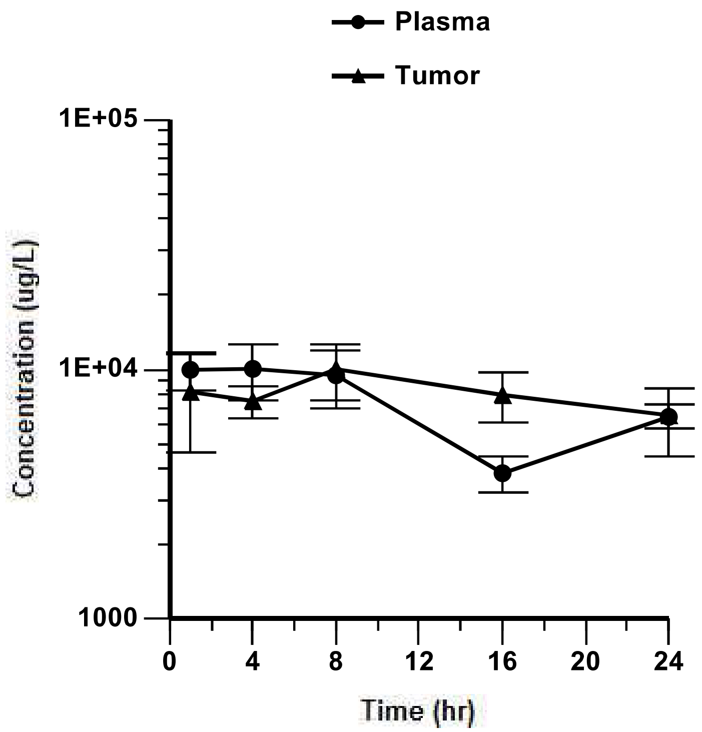

Table 4.1: NCA PK Parameter Estimates of CX-5461 by Group

		Analyte	
		CX-5461	
		Group	
		Plasma	Tumor
Parameter	Units	Estimate	
Cmax	ug/L	10000	10000
Tmax	hr	4.00	8.00
AUClast	hr*ug/L	165000	191000
AUCinf	hr*ug/L	376000	435000
Kel	1/hr	0.0241	0.0266
T1/2	hr	28.8	26.0

		Analyte	
		CX-5461	
		Group	
		Plasma	Tumor
Parameter	Units	Estimate	
CL/F	L/hr/kg	0.0664	0.0575
Vz/F	L/kg	2.76	2.16
Clast	ug/L	6450	6550
Tlast	hr	24.0	24.0
Kp,inf	-	-	1.16
Kp,last	-	-	1.16

Table 4.2: Full Summary Statistics of CX-5461 Ct Data by Group

		Analyte	
		CX-5461	
		Group	
		Plasma	Tumor
Time (hr)		Concentration (ug/L)	
1.000	N	3	3
	Mean	9960	8130
	SD	1780	3440
	Min	8140	5960
	Median	10000	6330
	Max	11700	12100
	CV%	17.9	42.3
	Geometric Mean	9850	7700
	CV% Geometric Mean	18.3	40.8
4.000	N	3	3
	Mean	10000	7470
	SD	2510	1060
	Min	7180	6830
	Median	11100	6890
	Max	11900	8690
	CV%	25.0	14.2

		Analyte	
		CX-5461	
		Group	
		Plasma	Tumor
Time (hr)		Concentration (ug/L)	
	Geometric Mean	9810	7420
	CV% Geometric Mean	27.8	13.7
8.000	N	3	3
	Mean	9480	10000
	SD	2520	2500
	Min	7570	7280
	Median	8520	10600
	Max	12300	12200
	CV%	26.6	24.9
	Geometric Mean	9270	9800
	CV% Geometric Mean	25.9	27.1
16.000	N	3	3
	Mean	3840	7900
	SD	656	1750
	Min	3100	5990
	Median	4100	8300
	Max	4330	9410
	CV%	17.1	22.1
	Geometric Mean	3800	7760
	CV% Geometric Mean	18.1	23.7
24.000	N	3	3
	Mean	6450	6550
	SD	1940	756
	Min	4240	6010
	Median	7250	6210
	Max	7850	7410
	CV%	30.1	11.5
	Geometric Mean	6230	6520
	CV% Geometric Mean	34.5	11.3

Table 4.3: CX-5461 Ct Data Listings by Subject, Analyte, Group, and Time

Subject	Analyte	Group	Time (hr)	Concentration (ug/L)
M1	CX-5461	Plasma	1.00	8138.10
M1	CX-5461	Tumor	1.00	6331.10
M2	CX-5461	Plasma	1.00	11690.00
M2	CX-5461	Tumor	1.00	5961.30
M3	CX-5461	Plasma	1.00	10040.00
M3	CX-5461	Tumor	1.00	12090.00
M4	CX-5461	Plasma	4.00	11103.00
M4	CX-5461	Tumor	4.00	8686.60
M5	CX-5461	Plasma	4.00	11851.00
M5	CX-5461	Tumor	4.00	6825.70
M6	CX-5461	Plasma	4.00	7178.30
M6	CX-5461	Tumor	4.00	6885.10
M7	CX-5461	Plasma	8.00	8518.50
M7	CX-5461	Tumor	8.00	7279.30
M8	CX-5461	Plasma	8.00	12335.00
M8	CX-5461	Tumor	8.00	10624.00
M9	CX-5461	Plasma	8.00	7574.30
M9	CX-5461	Tumor	8.00	12165.00
M10	CX-5461	Plasma	16.00	4099.10
M10	CX-5461	Tumor	16.00	9413.70
M11	CX-5461	Plasma	16.00	3095.60
M11	CX-5461	Tumor	16.00	5988.80
M12	CX-5461	Plasma	16.00	4329.00
M12	CX-5461	Tumor	16.00	8297.00
M13	CX-5461	Plasma	24.00	4237.00
M13	CX-5461	Tumor	24.00	7409.90
M14	CX-5461	Plasma	24.00	7249.40
M14	CX-5461	Tumor	24.00	6212.30
M15	CX-5461	Plasma	24.00	7854.90
M15	CX-5461	Tumor	24.00	6013.00

Table 4.4: CX-5461 Ct Summary (Mean, SD, N) by Group

Variable	Units	Analyte	Group	Time (hr)	Mean (ug/L)	SD (ug/L)	N
Concentration	ug/L	CX-5461	Plasma	1.00	9956.03	1777.44	3.00
Concentration	ug/L	CX-5461	Plasma	4.00	10044.10	2509.88	3.00
Concentration	ug/L	CX-5461	Plasma	8.00	9475.93	2520.63	3.00
Concentration	ug/L	CX-5461	Plasma	16.00	3841.23	655.89	3.00
Concentration	ug/L	CX-5461	Plasma	24.00	6447.10	1937.80	3.00
Concentration	ug/L	CX-5461	Tumor	1.00	8127.47	3436.63	3.00
Concentration	ug/L	CX-5461	Tumor	4.00	7465.80	1057.66	3.00
Concentration	ug/L	CX-5461	Tumor	8.00	10022.77	2497.72	3.00
Concentration	ug/L	CX-5461	Tumor	16.00	7899.83	1746.65	3.00
Concentration	ug/L	CX-5461	Tumor	24.00	6545.07	755.57	3.00
