# Supplementary material for: The chemotherapeutic CX-5461 primarily targets TOP2B and exhibits selective activity in high-risk neuroblastoma
Source: Nat Commun. 2021 Nov 9;12:6468. doi: 10.1038/s41467-021-26640-x (PMC8578635; doi:10.1038/s41467-021-26640-x)
Supplement: Supplementary file 2 — Description of Additional Supplementary Files [file 41467_2021_26640_MOESM2_ESM.docx]

**Description of Additional Supplementary Files**

Title: Supplementary Data 1

Description: All compounds screened in the GDSC ranked by their selectivity for neuroblastoma cell lines, as defined by our Selectivity Score, which is defined by the scaled (between 0 and 1) rank of the IC50 of the median neuroblastoma cell line. Annotations in columns "Target" and "Target.Pathway" were provided by GDSC.

Title: Supplementary Data 2

Description: Results of analysis of association of TOP2B and TOP2A expression with clinical and genomics features using the dataset assembled by Brady et al. A positive direction of effect means that feature increased expression of that gene. E.g. a positive effect for 3p_del on TOP2B expression would mean that deletion of the 3 arm of chromosome 3 increased expression of TOP2B. A negative effect would indicate that it decreased the expression of TOP2B.

Title: Supplementary Data 3

Description: Results of our first mouse study in the SJNBL046_X, MYCN-amplified Neuroblastoma mouse. US, Ultrasound;

Title: Supplementary Data 4

Description: Results of our first mouse study in the SJNBL047443_X, ATRX Mutant Neuroblastoma mouse

Title: Supplementary Data 5

Description: P-values calculated from a log-rank test for comparisons of all survival curves in the first mouse study, for the MYCN amplified SJNBL046 PDX tumors.

Title: Supplementary Data 6

Description: P-values calculated from a log-rank test for comparisons of all survival curves in the first mouse study, for the ATRX mutant SJNBL047443 PDX tumors.

Title: Supplementary Data 7

Description: Results of our 2nd mouse study in the SJNBL046_X, MYCN-amplified Neuroblastoma mouse

Title: Supplementary Data 8

Description: Results of our 2nd mouse study in the SJNBL047443_X, ATRX Mutant Neuroblastoma mouse model.

Title: Supplementary Data 9

Description: P-values calculated from a log-rank test for comparisons of all survival curves in the second mouse study, for the MYCN amplified SJNBL046 PDX tumors.

Title: Supplementary Data 10

Description: P-values calculated from a log-rank test for comparisons of all survival curves in the second mouse study, for the ATRX mutant SJNBL047443 PDX tumors.

Title: Supplementary Data 11

Description: Primers for quantitative real-time RT-PCR.
