# Supplementary material for: The chemotherapeutic CX-5461 primarily targets TOP2B and exhibits selective activity in high-risk neuroblastoma
Source: Nat Commun. 2021 Nov 9;12:6468. doi: 10.1038/s41467-021-26640-x (PMC8578635; doi:10.1038/s41467-021-26640-x)
Supplement: Supplementary file 14 — Reporting Summary [file 41467_2021_26640_MOESM14_ESM.pdf]

nature portfolio

Corresponding author(s): Paul Geeleher
Last updated by author(s): 2021/9/16

Reporting Summary

Nature Portfolio wishes to improve the reproducibility of the work that we publish. This form provides structure for consistency and transparency in reporting. For further information on Nature Portfolio policies, see our Editorial Policies and the Editorial Policy Checklist.

Statistics

For all statistical analyses, confirm that the following items are present in the figure legend, table legend, main text, or Methods section.

n/a	Confirmed	
☐	☒	The exact sample size (*n*) for each experimental group/condition, given as a discrete number and unit of measurement
☐	☒	A statement on whether measurements were taken from distinct samples or whether the same sample was measured repeatedly
☐	☒	The statistical test(s) used AND whether they are one- or two-sided<br>*Only common tests should be described solely by name; describe more complex techniques in the Methods section.*
☐	☒	A description of all covariates tested
☐	☒	A description of any assumptions or corrections, such as tests of normality and adjustment for multiple comparisons
☐	☒	A full description of the statistical parameters including central tendency (e.g. means) or other basic estimates (e.g. regression coefficient) AND variation (e.g. standard deviation) or associated estimates of uncertainty (e.g. confidence intervals)
☐	☒	For null hypothesis testing, the test statistic (e.g. *F*, *t*, *r*) with confidence intervals, effect sizes, degrees of freedom and *P* value noted<br>*Give P values as exact values whenever suitable.*
☒	☐	For Bayesian analysis, information on the choice of priors and Markov chain Monte Carlo settings
☒	☐	For hierarchical and complex designs, identification of the appropriate level for tests and full reporting of outcomes
☐	☒	Estimates of effect sizes (e.g. Cohen's *d*, Pearson's *r*), indicating how they were calculated

Our web collection on statistics for biologists contains articles on many of the points above.

Software and code

Policy information about availability of computer code

Data collection	No such software was used for data collection.
Data analysis	No such software was used for data analysis.

For manuscripts utilizing custom algorithms or software that are central to the research but not yet described in published literature, software must be made available to editors and reviewers. We strongly encourage code deposition in a community repository (e.g. GitHub). See the Nature Portfolio guidelines for submitting code & software for further information.

Data

Policy information about availability of data

All manuscripts must include a data availability statement. This statement should provide the following information, where applicable:

- Accession codes, unique identifiers, or web links for publicly available datasets
- A description of any restrictions on data availability
- For clinical datasets or third party data, please ensure that the statement adheres to our policy

The main data generated in this study are included in the article and its Supplementary files. The source data for Figs. 1-4, Fig. 7, Supplementary Figs. 1-4 and Supplementary Figs. 6-7 are provided in Source Data file. The publicly available data used in this study are obtained from GDSC, PRISM, TCGA, TARGET and GTEx. Further information of the data sources is stated in figure legends and Methods. Details of additional data that support the findings of this study will be available upon reasonable request.

Field-specific reporting

Please select the one below that is the best fit for your research. If you are not sure, read the appropriate sections before making your selection.

- [x] Life sciences
- [ ] Behavioural & social sciences
- [ ] Ecological, evolutionary & environmental sciences

For a reference copy of the document with all sections, see nature.com/documents/nr-reporting-summary-flat.pdf

Life sciences study design

All studies must disclose on these points even when the disclosure is negative.

Sample size	No sample size calculations were performed.
Data exclusions	No data were excluded.
Replication	Number of replication with similar results are stated in figure legends and Methods section.
Randomization	Allocation was at random.
Blinding	Investigators were not blinded.

Reporting for specific materials, systems and methods

We require information from authors about some types of materials, experimental systems and methods used in many studies. Here, indicate whether each material, system or method listed is relevant to your study. If you are not sure if a list item applies to your research, read the appropriate section before selecting a response.

Materials & experimental systems

n/a	Involved in the study	
[ ]	[x]	Antibodies
[ ]	[x]	Eukaryotic cell lines
[x]	[ ]	Palaeontology and archaeology
[ ]	[x]	Animals and other organisms
[x]	[ ]	Human research participants
[x]	[ ]	Clinical data
[x]	[ ]	Dual use research of concern

Methods

n/a	Involved in the study	
[x]	[ ]	ChIP-seq
[ ]	[x]	Flow cytometry
[x]	[ ]	MRI-based neuroimaging

Antibodies

Antibodies used	β-Actin (Sigma, #A1978; 1:20,000 dilution), cleaved PARP (Cells Signaling, #5625; 1:1,000 dilution), TOP1 (Santa Cruz, #sc-271285; 1:200 dilution), TOP2A (Santa Cruz, #sc-365916; 1:200 dilution), TOP2B (BD Biosciences, #611492; 1:2,000 dilution), P53 (Santa Cruz, #sc-126; 1:200 dilution), phospho-P53 S15 (Cell signaling, #9284; 1:1,000 dilution), pCHK1 S345 (Cell Signaling, #2348; 1:1,000 dilution), CHK1 (Santa Cruz, #sc-8408; 1:200 dilution), pCHK2 T68 (Cell Signaling, #2661; 1:1,000 dilution), CHK2 (Santa Cruz, #sc-17747; 1:100 dilution), HRP-linked anti-rabbit IgG (Cell Signaling, #7074; 1:2,000 dilution), HRP-linked anti-mouse IgG (Cell Signaling, #7076; 1:2,000 dilution), γ-H2AX (Cell Signaling, #9718; Millipore, # 05-636; 1:500 dilution), 53BP1 (Abcam, ab36823; 1:500 dilution), RPA (Millipore, # MABE285; 1:500 dilution), Alexa fluor 488 conjugated goat anti-mouse IgG (Thermo Fisher, #A-11001; 1:1,000 dilution), Alexa fluor® 594 conjugate anti-rabbit IgG (Cell Signaling, 8889; 1:1,000 dilution).
Validation	All these antibodies have been validated for western blotting in human cells (information can be found on the vendor's websites): β-Actin (Sigma, #A1978), cleaved PARP (Cells Signaling, #5625), TOP1 (Santa Cruz, #sc-271285), TOP2A (Santa Cruz, #sc-365916), TOP2B (BD Biosciences, #611492), P53 (Santa Cruz, #sc-126), phospho-P53 S15 (Cell signaling, #9284), pCHK1 S345 (Cell Signaling, #2348; 1:1,000 dilution), CHK1 (Santa Cruz, #sc-8408; 1:200 dilution), pCHK2 T68 (Cell Signaling, #2661), CHK2 (Santa Cruz, #sc-17747), HRP-linked anti-rabbit IgG (Cell Signaling, #7074), HRP-linked anti-mouse IgG (Cell Signaling, #7076).<br>All these antibodies have been validated for immunofluorescence in human cells (information can be found on the vendor's websites): γ-H2AX (Cell Signaling, #9718; Millipore, # 05-636), 53BP1 (Abcam, ab36823), RPA (Millipore, # MABE285), Alexa fluor 488 conjugated goat anti-mouse IgG (Thermo Fisher, #A-11001), Alexa fluor® 594 conjugate anti-rabbit IgG (Cell Signaling, 8889).<br>These antibodies have been validated for flow cytometry on the vendor's websites: γ-H2AX (Cell Signaling, #9718), Alexa fluor® 594 conjugate anti-rabbit IgG (Cell Signaling, 8889).

Eukaryotic cell lines

Policy information about cell lines

Cell line source(s)	CHP-134, KELLY, and SK-N-SH cells were purchased from Sigma (MilliporeSigma, USA). BE(2)-M17, SK-N_FI, RS4-11, H2452, and 293T cells were purchased from ATCC. IMR-5 cell line was a gift from the Cellular Screening Center at the University of Chicago.
Authentication	The cell lines were not authenticated.
Mycoplasma contamination	All cell lines were tested negative for mycoplasma using the MycoAlert mycoplasma detection kit (Lonza, LT07-118).
Commonly misidentified lines (See ICLAC register)	No commercially misidentified cell lines were used in this study.

Animals and other organisms

Policy information about studies involving animals; ARRIVE guidelines recommended for reporting animal research

Laboratory animals	Female athymic nude mice (Charles River strain code 553).
Wild animals	No wild animals were used in this study.
Field-collected samples	No field-collected samples were used in this study.
Ethics oversight	The study protocol was approved by the Institutional Animal Care and Use Committee at St. Jude Children's Research Hospital.

Note that full information on the approval of the study protocol must also be provided in the manuscript.

Flow Cytometry

Plots

Confirm that:

- [x] The axis labels state the marker and fluorochrome used (e.g. CD4-FITC).
- [x] The axis scales are clearly visible. Include numbers along axes only for bottom left plot of group (a 'group' is an analysis of identical markers).
- [x] All plots are contour plots with outliers or pseudocolor plots.
- [x] A numerical value for number of cells or percentage (with statistics) is provided.

Methodology

Sample preparation	For apoptosis, cells were treated in 6-well plates. After incubation for the time needed, cell culture medium and cells were collected and washed in cold phosphate-buffered saline (PBS). The cells were then resuspended in annexin-binding buffer and stained with Alexa Fluor 488 conjugated annexin V (Thermo Fisher, catalog# A13201) and 1 µg/ml Propidium iodide (PI) for flow cytometry analysis. For cell cycle analysis, cells were fixed with cold 70% ethanol in water. After 2× washes in PBS, cells were treated with 100 µg/ml RNase A and stained with 50 µg/ml PI for flow cytometry analysis. For γ-H2AX staining, cells were fixed with 4% formaldehyde and permeabilized with 90% methanol, then stained with γ-H2AX (Cell Signaling, #9718) and Alexa fluor® 594 conjugate anti-rabbit IgG (Cell Signaling, 8889) for flow cytometry.
Instrument	BD LSRFortess X-20
Software	BD FACSDiva and FlowJo
Cell population abundance	10,000 events were collected for each replicate. Single cells were used for analysis. Single cells were defined by cell size using FSC-A and SSC-W. Details are included in Supplementary figure 8.
Gating strategy	Detailed gating strategies are included in Supplementary figure 8.

- [x] Tick this box to confirm that a figure exemplifying the gating strategy is provided in the Supplementary Information.
